# Catalytic asymmetric Friedel–Crafts alkylation of unprotected indoles with nitroalkenes using a novel chiral Yb(OTf)_3_–pybox complex

**DOI:** 10.1038/s41598-023-41921-9

**Published:** 2023-09-07

**Authors:** Babak Karimi, Ehsan Jafari, Fariborz Mansouri, Mina Tavakolian

**Affiliations:** 1 Department of Chemistry, Institute for Advanced Studies in Basic Sciences (IASBS), Prof. Sobouti Boulevard, PO-Box 45195-1159, Zanjan, 45137-66731 Iran; 2 Research Center for Basic Sciences & Modern Technologies (RBST), Institute for Advanced Studies in Basic Sciences (IASBS), Prof. Sobouti Boulevard, Zanjan, 45137-66731 Iran

**Keywords:** Asymmetric catalysis, Catalyst synthesis, Synthetic chemistry methodology

## Abstract

Chiral chloro-indeno pybox has served as a new ligand for the Yb(OTf)_3_-catalyzed asymmetric Friedel–Crafts alkylation reaction of indoles with nitroalkenes. The tunable nature of pybox ligands enables the rational design of catalysts for optimal performance in terms of both activity and stereoselectivity in a Friedel–Crafts-type reaction. Good to excellent yields and enantioselectivities were obtained for a relatively wide range of substrates, including sterically hindered compounds, under optimized reaction conditions.

## Introduction

The enantioselective Freidel–Crafts (FC) addition of heteroaromatic compounds to nitroalkenes is an important reaction that enables the formation of valuable synthetic building blocks and has become a powerful tool in organic synthesis^1–7^. Indole-containing motifs are always considered to be appealing synthetic molecules due to their prevalence in numerous bioactive substances and pharmaceutical compounds^8–11^. In particular, nitroalkenes are highly desirable Michael acceptors because the nitro group can be readily converted into a range of different functionalities including tryptamines, *β*-carbolines, or indole alkaloids^12^. Over the past decades, many efforts have been made to develop asymmetric FC reactions of indole derivatives with nitroalkenes using both metal-based chiral complexes^13–27^ and organocatalysts^28–39^.

**Scheme 1:**
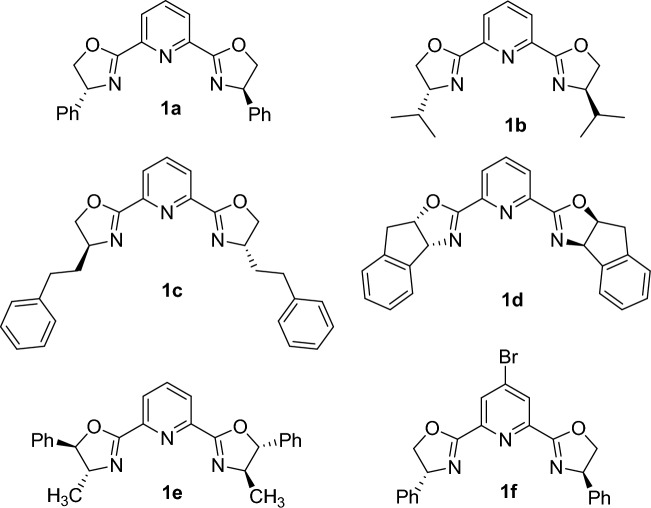
Structure of various pybox ligands.

Since the first report by Nishiyama in 1989, tridentate (-2,6-bis[(4*S*)-R-2-oxazolin-2-yl]pyridine ligands (pybox) have emerged as versatile chiral ligands in asymmetric synthesis (Scheme 1)^40^.

The pybox ligands can simultaneously coordinate to the metal center via two nitrogen atoms of the two oxazolines and one pyridine ring to provide highly robust C_2_-chiral complexes. Moreover, the electronic properties of these complexes can be easily adjusted by replacing the hydrogen atom at the 4-position of the pyridine moiety in the pybox skeletons. This provides a way to tune the catalytic activity and, more specifically, their enantioselectivity in a particular asymmetric transformation^41–44^.

Taking into consideration this criterion, a range of metal/pybox complexes have been prepared and used in diverse asymmetric reactions so far^45–58^. However, despite the higher performance of Ytterbium/pybox complexes compared to other metal/pybox complexes in asymmetric catalytic reactions, the use of these complexes has been limited to only a few asymmetric reactions^59–66^. Furthermore, while there have been numerous reports on the utilization of chiral pybox/metal complexes as effective catalysts in different types of asymmetric transformations, to the best of our knowledge, there is currently no practical procedure available for the use of chiral pybox/metal complexes in the asymmetric FC type reaction of unprotected indole and nitroalkene derivatives. In recent years, we have demonstrated the utility of various chiral pybox/Yb(OTf)_3_ complexes as efficient and highly enantioselective catalysts in several beneficial asymmetric transformations including Strecker and Mannich reactions^63–66^. During these studies, we found that chiral induction could be greatly influenced by the structure and electronic features of the pybox ligand used to form ligand/metal complexes. Accordingly, here we wish to report a new class of functionalized pybox ligand that can catalyze the asymmetric FC reaction of indole and nitroalkene derivatives.

## Results and discussion

The main impetus for the present study is to investigate whether the electronic-structural synergism in a novel pybox ligand can amplify the catalytic performance (both enantioselectivity and activity) of pybox-Yb(OTf)_3_ in the asymmetric FC type reaction of indole and nitroalkenes. To test this hypothesis, Indole **3a** was initially chosen to react with trans-*β*-nitrostyrene **2a** as a representative substrate in order to determine the optimal reaction conditions (Table 1). In this way, the impact of various parameters such as the nature of ligand and metal, solvent, temperature and ligand to metal ratio were investigated. The results of these investigations are summarized in Table 1. Initially, we examined the asymmetric Friedel–Crafts alkylation of indole **3a** with *β*-nitrostyrene **2a** using in-situ complexes generated from Yb(OTf)_3_ and the desired ligands **1a–f** at room temperature (Scheme 1). Preliminary studies revealed that among various pybox ligands were applied (Table 1, entries 1–6), ligand **1d** in combination with Yb(OTf)_3_ was the best choice, resulting in the corresponding FC adduct in 81% and 44% enantiomeric excess (Table 1, entry 4). In a same manner, the impact of a series of metal triflates such as Sc(OTf)_3_, Zn(OTf)_2_, Cu(OTf)_2_ and In(OTf)_3_ were sequentially examined in the reaction between indole **3a** with *β*-nitrostyrene **2a**. The results showed that these metal triflates led to lower enantioselectivity under otherwise identical reaction conditions compared to the same reaction using Yb(OTf)_3_ (Table 1, entries 7–10). The screening of solvents showed that CH_2_Cl_2_ (DCM) was the best solvent, resulting in to superior enantioselectivity and yield of the FC adduct compared to the solvents such as THF, toluene and CH_3_CN, (Table 1, entry 4 vs. entries 11–13). Based on our previous experiences^63–66^, we envisioned that higher enantioselectivity and reactivity might also be achieved by using a suitable protic additive. Accordingly, in the next stage, we tested various protic additives such as methanol and hexafloroisopropanol (HFIP). It was found that the enantioselectivity of the final product decreased in the presence of these additives, although the yield of the product slightly improved in these conditions (Table 1, entries 14–16). In the following experiments, the effect of varying temperature on the reaction in the absence of protic additive was investigated.

**Table 1:** Optimization of the reaction conditions for the asymmetric FC reaction of nitroalkane 2a and indole 3a.


Entry	Ligand/metal	Solvent/additive	T (°C)	Yield (%)^a,b^	ee (%)^c,d^
1	**1a**/Yb(OTf)_3_	DCM	rt	61	27
2	**1b**/Yb(OTf)_3_	DCM	rt	65	20
3	**1c**/Yb(OTf)_3_	DCM	rt	79	13
4	**1d**/Yb(OTf)_3_	DCM	rt	81	44
5	**1e**/Yb(OTf)_3_	DCM	rt	24	5
6	**1f**/Yb(OTf)_3_	DCM	rt	80	30
7	**1d**/Sc(OTf)_3_	DCM	rt	30	10
8	**1d**/Zn(OTf)_2_	DCM	rt	20	5
9	**1d**/Cu(OTf)_2_	DCM	rt	25	3
10	**1d**/In(OTf)_3_	DCM	rt	40	10
11	**1d**/Yb(OTf)_3_	THF	rt	51	37
12	**1d**/Yb(OTf)_3_	PhCH_3_	rt	42	20
13	**1d**/Yb(OTf)_3_	CH_3_CN	rt	45	30
14	**1d**/Yb(OTf)_3_	DCM/MeOH (1 equiv.)	rt	80	27
15	**1d**/Yb(OTf)_3_	DCM/HFIP (1 equiv.)	rt	90	15
16	**1d**/Yb(OTf)_3_	DCM/Et_3_N (1 equiv.)	rt	N.R	N.D
17	**1d**/Yb(OTf)_3_	DCM	− 20	57	48
18	**1d**/Yb(OTf)_3_	DCM	− 50	43	20
19	**1d**/Yb(OTf)_3_	DCM/MeOH (1 equiv.)	− 20	81	60
20	**1d**/Yb(OTf)_3_	DCM/MeOH (1 equiv.)	− 30	52	60
21	**1d**/Yb(OTf)_3_	DCM/MeOH (1 equiv.)	− 40	40	40
22	**1d**/Yb(OTf)_3_	DCM/MeOH (1.5 equiv.)	− 20	82	45
23^e^	**1d**/Yb(OTf)_3_	DCM/MeOH (1 equiv.)	− 20	83	50
24f.	**1d**/Yb(OTf)_3_	DCM/MeOH (1 equiv.)	− 20	46	50

^a^The isolated yield. ^b^Conditions: 1 mmol nitrostyrene **2a**, 1.1 mmol indole **3a**. ^c^The ee of products were determined by HPLC [Chiralpak AD-H, 90–10 n-hexane/*i*-PrOH, 0.5 mL/min]. ^d^Absolute configuration was determined to be *R* according to the literature data. ^e^The molar ratio of ligand: metal = 15:10 (mol%). ^f^The molar ratio of ligand: metal = 5:5(mol%).

It was found that the enantioselectivity was amplified by reducing the reaction temperature to − 20 °C, although there was a noticeable decline in chemical yield (Table 1, entry 17). Further decreasing the reaction temperature (to − 50 °C) resulted in significantly lower enantioselectivity and yield (Table 1, entry 18). In the next stage, we decided to investigate the combined effect of lower temperature and protic additive on the reaction outcome, using the specified reaction conditions. Among different reaction temperatures tested, ranging from − 20 to − 40 °C, in the presence of 1 equivalent MeOH (Table 1, entries 19–21), our catalyst derived from Yb(OTf)_3_ and **1d** exhibited the highest yield (81%) and enantioselectivities (60% ee) at − 20 °C (Table 1, entry 19).

Then, the impact of MeOH concentration as an additive in the reaction under otherwise the same conditions was explored. As can be seen in entry 22 of Table 1, increasing the amount of MeOH to 1.5 equivalent (with respect to substrates) resulted in lower yield and enantioselectivity. Therefore, 1 equivalent of MeOH was used for the subsequent studies. To gain a comprehensive understanding of the role of methanol (MeOH) in our reaction process, it is crucial to undertake a meticulous investigation into the exact alterations in catalyst structure during the progression of the reaction. We are currently engaged in efforts to gain mechanistic insights into the impact of MeOH as a protic additive in our transformation, with the aim of determining its precise involvement and role in enhancing the reaction outcomes. Previous studies conducted by various research groups have successfully undertaken mechanistic investigations on this subject matter^67,68^. We will be back to the role of MeOH in the manuscript when we briefly examine the proposed mechanism of the process.

Next, the molar ratio of ligand to metal was investigated in the next step. It was observed that a 1:1 ratio of ligand to metal was more efficient in achieving desirable enantioselectivity compared to 1.5 to 1, as demonstrated in Table 1 (entry 23 vs. entry 19). Additionally, it was observed that decreasing the loading of both ligand and metal to 5 mol%, resulted in a detrimental impact on both yield and ee (Table 1, Entry 24). Finally, the conditions specified in entry 19 of Table 1 were chosen as the optimal conditions for the subsequent studies.

We have previously shown that the pybox ligand, which has an electron withdrawing bromine group at the 4-position of the pyridine moiety, in combination with Yb(OTf)_3_, can remarkably enhance both the enantioselectivity and yield of the desired products in the asymmetric Strecker reaction of imines with TMSCN (affording up to 95% yield and 97% ee)^63^. Encouraged by this result, we hypothesize that incorporating an electron-withdrawing group such as bromo or chloro at the 4-position of **1d** ligand might have a similar effect on the asymmetric FC reaction of nitroalkene **2a** with indole **3a**. Our initial investigations, using 4-Br-pybox bearing ligand phenyl group (**1f**) that we had developed previously, in the reaction of nitroalkene **2a** with indole **3a** under similar conditions, only resulted in a slight improvement in the enantioselectivity (Table 1, entry 6).

**Scheme 2:**
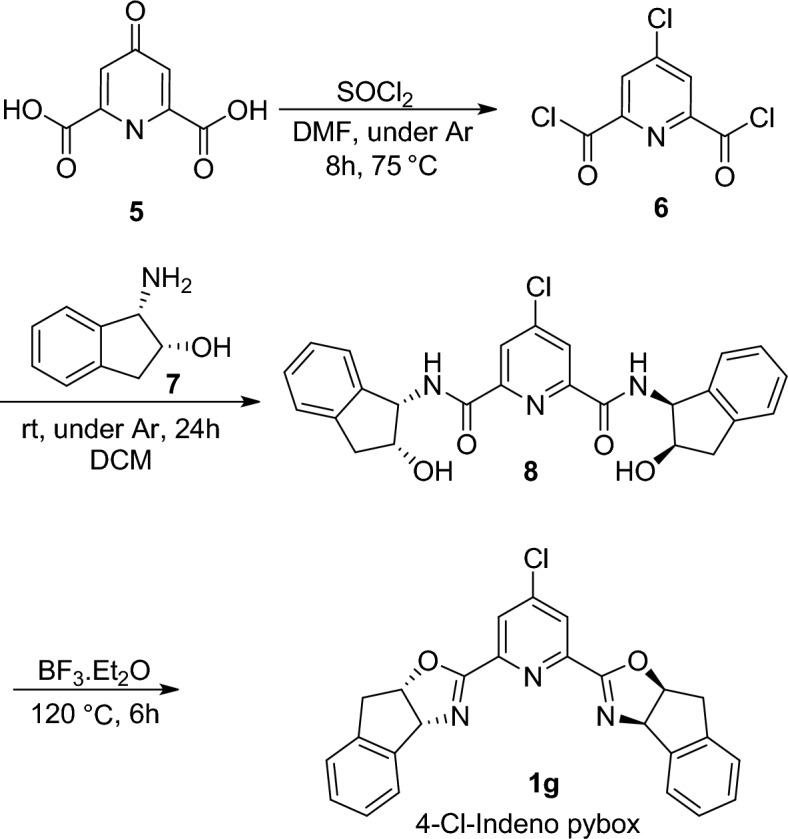
Synthesis of Cl-indeno pybox **1g**.

Based on this result and considering the fact that indeno-pybox (**1d**) demonstrated superior results in the model FC reaction under optimized experiments (Table 1), we next turned our attention to preparing a corresponding indeno-pybox ligand containing a chlorine atom at the 4-position of pyridine (**1g**). According to the previously reported protocol for the synthesis of indeno-pybox ligands^69,70^, we embarked on preparing a new pybox ligand with a chlorine group as a withdrawing group on the pyridine moiety, as outlined in Scheme 2. In this experiment, chelidamic acid **5**, which is commercially available, was allowed to react with thionyl chloride in the presence of dimethyl formamide (DMF) under an Ar atmosphere. This reaction resulted in the formation of 4-chloropyridine-2,6-diacyl chloride **6**. Subsequently, (1*S*,2*R*)-aminoindanol **7** was added to a solution of **6** in anhydrous DCM at room temperature, affording the corresponding bisamide **8**. This compound was finally converted to the desired 4-chloro indeno-pybox **1g** after cyclization in a sealed tube in the presence of BF_3_**.**Et_2_O as a catalyst (Scheme 2).

**Scheme 3:**
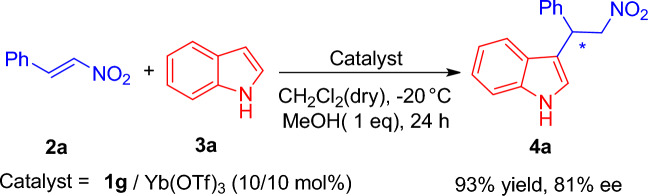
The asymmetric FC reaction of nitroalkene 2a with indole 3a using Cl-indeno pybox **1g**.

With this new ligand available, we proceeded to investigate the asymmetric FC reaction of trans-*β*-nitrostyrene **2a** with indole **3a** using the conditions outlined in entry 19. Gratifyingly, we observed a substantial improvement in both the enantioselectivity and the yield of product **4a**, reaching 81% and 93%, respectively (Scheme 3).

Encouraged by this promising data, we then tested the scope of the present asymmetric Friedel–Crafts alkylation protocol through catalysis by 4-Cl-indeno pybox **1g**/Yb(OTf)_3_ (Table 2).

**Table 2:** The asymmetric Friedel–Crafts alkylation of nitroelkenes 2 with indole derivatives 3 using Cl-indeno pybox **1g**/Yb(OTf)_3_.


Entry	R^1^	R^2^	Product	Yield (%)^a,b^	ee (%)^c,d^
1	H	Ph	**4a**	93	81
2	H	4-ClC_6_H_4_	**4b**	95	87
3	H	3-ClC_6_H_4_	**4c**	92	83
4	H	2-ClC_6_H_4_	**4d**	93	89
5	H	3-BrC_6_H_4_	**4e**	96	86
6	H	2-BrC_6_H_4_	**4f**	96	83
7	H	3-NO_2_C_6_H_4_	**4g**	98	84
8	H	2-NO_2_C_6_H_4_	**4h**	98	91
9	H	4-MeC_6_H_4_	**4i**	91	83
10	H	4-MeOC_6_H_4_	**4j**	88	85
11	H	3-MeOC_6_H_4_	**4k**	90	84
12	H	1-naphtyl	**4l**	92	82
13	H	2-Furyl	**4m**	87	84
14	Cl	Ph	**4n**	88	81
15	Cl	3-BrC_6_H_4_	**4o**	89	81
16	Cl	2-NO_2_C_6_H_4_	**4p**	91	83
17	Cl	4-MeOC_6_H_4_	**4q**	84	79
18	OMe	Ph	**4r**	95	84
19	OMe	3-BrC_6_H_4_	**4s**	95	89
20	OMe	2-NO_2_C_6_H_4_	**4t**	98	88
21	OMe	4-MeOC_6_H_4_	**4u**	91	83

^a^The isolated yield. ^b^Conditions: 1 mmol nitroalkene, 1.1 mmol indole. ^c^The ee of products were determined by HPLC [Chiralpak AD-H, 90–10 n-hexane/*i*-PrOH, 0.5 mL/min]. ^d^Absolute configuration was determined to be *R* according to the literature data. (See Supporting Information file for experimental details)

As shown in Table 2, regardless of the steric hindrance or electronic properties of aryl substituents, aromatic nitroalkenes smoothly reacted with unprotected indole derivatives and furnished the corresponding FC adducts in excellent yields and high enantioselectivities (Table 2). It was noteworthy that several catalytic systems experienced lower enantioselectivities due to steric hindrance caused by *ortho* substitution of the phenyl group in aromatic nitroalkenes^71,72^. On the contrary, in this system, the enantioselectivity increased instead of decreased when the *ortho*-substituted substrate was used (Table 2, entries 4, 6 and 8). Furthermore, heteroaromatic nitroalkenes, which typically suppress the performance of metal catalysts, also served as good substrates in our system, yielding very positive outcomes (Table 2, entry 13). In addition, the substituent effect of indole was also investigated (Table 2, entries 14–21). The electronic properties of the substituent at the 5-position of indole had little effect on enantioselectivities, but slightly lower yields were observed when an electron-withdrawing group was employed (Table 2, entries 14–17).

**Scheme 4:**
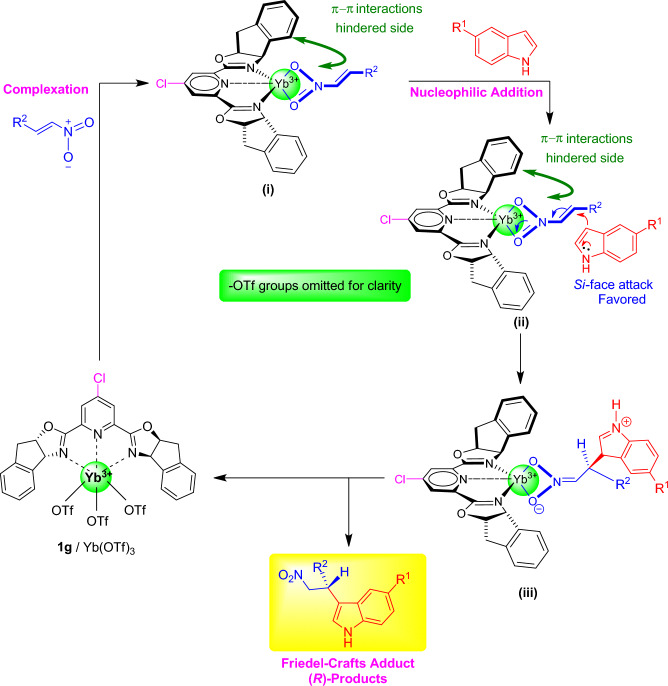
Proposed reaction pathway for the Friedel–Crafts alkylation of indoles and nitroalkenes using Yb(OTf)_3_/Cl-indeno pybox **1g** catalyst.

Although a mechanistic investigation was not conducted in this study, a plausible reaction pathway for the Friedel–Crafts alkylation of indoles and nitroalkenes using our developed Yb(OTf)_3_/Cl-indeno pybox **1g** catalyst is proposed as described in Scheme 4. Based on the proposed mechanism, *β*-nitrostyrene is initially activated through complexation with the Yb(OTf)_3_/Cl-indeno pybox **1g** catalyst, forming intermediate (**i**). This intermediate undergoes a nucleophilic addition of indole from the more favored *Si-*face of *β*-nitrostyrene via transition state (**ii**) to yield intermediate (**iii**). After an H-transfer step, and dissociation of FC adduct with high *R-*enantioselectivity**,** the catalyst Yb(OTf)_3_/Cl-indeno pybox **1g** was regenerated and re-entered into the next cycle of asymmetric Friedel–Crafts reaction. At this stage, one possible explanation about the potential role of MeOH is that it may facilitate the final step of the reaction by either rapidly exchanging protons within adduct (**iii**) or activating indole in transition state (**ii**) through hydrogen bonding.

## Conclusions

In conclusion, a highly enantioselective Friedel–Crafts alkylation of unprotected indole and nitroalkene derivatives has been successfully developed using a Yb(OTf)_3_/Cl-indeno pybox 1 g catalyst system. The reaction exhibited favorable performance when applied to a range of indoles and nitroalkenes, specifically emphasizing the successful reaction with sterically hindered aromatic nitroalkenes possessing ortho substitution. The reaction resulted in the formation of the desired products, achieving high yields of up to 98% and enantioselectivities of up to 91% ee. It is noteworthy that this study presents the first example of an asymmetric Friedel–Crafts reaction involving unprotected indole derivatives using Yb(OTf)_3_ as a catalyst, in conjunction with novel chloro-functionalized indeno-pybox ligands.

## Methods

### General information

^1^H-NMR spectra were recorded on commercial instruments (400 and 600 MHz). Chemical shifts were reported in ppm from tetramethylsilane with the solvent resonance as the internal standard (CDCl_3_: δ = 7.26). Spectra are reported as follows: chemical shift (= ppm), multiplicity (s = singlet, d = doublet, t = triplet, q = quartet, m = multiplet), coupling constants (Hz), integration. ^13^C-NMR spectra were collected on commercial instruments (100 MHz) with complete proton decoupling. The enantiomeric excesses were determined by HPLC analysis on CHIRALPAK-AD and DAICELIA.M columns. Optical rotations were measured on a commercial polarimeter and reported as follows: [α]_D_^T^ (c = g/100 mL, solvent). Reagents obtained from commercial sources were used without further purification. Ligand **3a–f** and indole **2** was purchased from Aldirch and Merck Inc, respectively. Methylene chloride was freshly distilled from calcium hydride under nitrogen atmosphere.

### General procedure for the preparation of Indeno pybox 1g

In a dry round-bottom flask equipped with a reflux condenser, thionyl chloride (1 ml) and commercially available chelidamic acid **5** (182 mg, 28.8 mml) was added in the presence of dimethyl formamide (1 drop) under Ar atmosphere. The reaction mixture was stirred for 8 h at reflux condition and after cooling to room temperature, the excess amount of thionyl chloride evaporated and the product **6** used for the next step immediately. In continuation, to a solution of **6** (119 mg, 0.5 mmol) was added (1*S*,2*R*)-Aminoindanol **7** (150 mg, 1 mmol) in dichloromethane (1 ml) and the reaction mixture was stirred rapidly at room temperature for 4 h. After completion the reaction, the product **8** was obtained after purification with column chromatography on silica gel (*n*-hexane/ethylacetate = 20:80). Finally, a solution of bis(amide) **8** (333 mg) in BF_3_.Et_2_O (2.6 g) was heated in a sealed tube to 120 °C (the mixture became homogeneous at 75 °C) for 6 h. After completion of the reaction, the solution was allowed to cool, diluted with dichloromethane, and poured into ice-cold 2N NaOH. The phases were separated, and the crude product was obtained after removal of solvent. The pure product **1g** was obtained after column chromatography on silica gel (*n*-hexane/ethylacetate = 60:40).

**Compound 8:** The crude material was purified by flash chromatography on silica gel (Ethyl acetate/Hexane, 80:20) to afford the product in 87% yield as colorless solid. ^**1**^**H NMR (600 MHz, CDCl**_**3**_**):** δ 2.86–2.82 (2H, m), 3.12–3.03 (2H, m), 4.48 (2H, br.s), 5.35–5.31 (4 H, m), 7.19–7.12 (8H, m), 8.29 (2H, s), 8.97–8.95 (2H, s); ^**13**^**C NMR (150 MHz, CDCl**_**3**_**):** δ 40.5, 57.6, 72.2, 124.6, 125.0, 125.3, 126.8, 127.9, 141.2, 142.0, 146.6, 151.2, 162.7.

**Compound 1g:** The crude material was purified by flash chromatography on silica gel (Ethyl acetate/Hexane, 40:60) to afford the product in 95% yield as white solid. mp 268–269 °C; ^**1**^**H NMR (600 MHz, CDCl**_**3**_**):** δ 3.56–3.44 (4H, m), 4.79 (2H, m), 5.55 (2H, m), 7.41–7.39 (6H, m), 7.49 (2H, m), 8.12 (2H, s); ^**13**^**C NMR (150 MHz, CDCl**_**3**_**):** δ 39.6, 79.0, 84.3, 125.4, 125.5, 126.8, 127.6, 128.8, 139.5, 141.2, 146.6, 151.2, 163.0; Anal. Calcd for C_25_H_18_N_3_O_2_Cl: C, 70.19; H, 4.20; N, 9.82. Found: C, 70.3; H, 5.06; N, 9.62; MS; *m*/*z*: 427 (*M*)+; IR 1667, 1522 cm^−1^.

### General procedure for the preparation of Ytterbium(III) triflate complex

A 2-dram oven-dried vial was charged with a stirbar, Yb(OTf)_3_ (30 mg, 0.048 mmol), and the corresponding pybox ligand (20.5 mg, 0.048 mmol) in a dry box. The vial was capped with a septum and removed from the dry box. Dichloromethane (1.0 mL) was added to the vial under an atmosphere of dry Ar. The resulting mixture was stirred vigorously at room temperature for 1 h until the reaction became homogeneous.

### General catalytic procedure

To the resulting complex solution, 0.5 mmol of nitroalkene was added and the mixture cooled to − 18 °C. At this temperature then indole derivatives (0.55 mmol) was added and then methanol (0.5 mmol, 16 mg) was injected in one portion. After 24 h, the reaction mixture was purified by silica gel flash chromatography (SiO_2_, hexane /Ethyl acetate; 100/40) to afford the desired product.

**Compound 4a:** The crude material was purified by flash chromatography on silica gel (Ethyl acetate/Hexane, 40:100) to afford the product in 93% yield as colorless oil. The chromatographed material was determined to be of 81% ee by chiral HPLC analysis [DAICELIA.M, 90–10 *n*-heptane/*i*PrOH, 0.7 mL/min, t_r_ (major) = 22.8 min, t_r_ (minor) = 25.3 min]; [α] _D_^20^ = -6.66 (c = 0.75 in CHCl_3_). ^**1**^**H NMR (600 MHz, CDCl**_**3**_**):** 8.08 (br s, 1H), δ 7.37–7.04 (m, 10H), 5.19 (t, *J* = 6 Hz, 1H), 5.09–5.05 (m, 1H), 4.96–4.93 (m, 1H); ^**13**^**C NMR (150 MHz, CDCl**_**3**_**):** δ 139.3, 137.2, 128.9, 127.7, 127.2, 124.1, 122.9, 121.4, 120.2, 118.9, 114.3, 111.3, 79.5, 41.2.

**Compound 4b:** The crude material was purified by flash chromatography on silica gel (Ethyl acetate/Hexane, 40:100) to afford the product in 95% yield as colorless oil. The chromatographed material was determined to be of 87% ee by chiral HPLC analysis [Chiralpak AD-H, 90–10 *n*-hexane/*i*PrOH, 0.5 mL/min, t_r_ (major) = 26.5 min, t_r_ (minor) = 36.2 min]; [α] _D_^20^ =  + 7.4 (c = 1.2 in CHCl_3_). ^**1**^**H NMR (600 MHz, CDCl**_**3**_**):** δ 8.20 (br s, 1H), 7.39 (d, *J* = 6 Hz, 1H), 7.35 (d, *J* = 6 Hz, 1H), 7.29–7.25 (m, 4H), 7.20 (t, *J* = 6 Hz, 1H), 7.08(t, *J* = 12 Hz, 1H), 7.00(s, 1H), 5.16 (t, *J* = 18 Hz, 1H), 5.06–5.03 (m, 1H), 4.92–4.88 (m, 1H); ^**13**^**C NMR (150 MHz, CDCl**_**3**_**):** δ 137.8, 136.3, 133.0, 129.7, 129.2, 125.7, 123.1, 121.6, 120.2, 118.9, 113.5, 11.6, 78.8, 41.4. **IR (KBr, cm**^**−1**^**):** 3418, 3056, 1720, 1549, 1421, 1250, 741.

**Compound 4c:** The crude material was purified by flash chromatography on silica gel (Ethyl acetate/Hexane, 40:100) to afford the product in 92% yield as colorless oil. The chromatographed material was determined to be of 83% ee by chiral HPLC analysis [Chiralpak AD-H, 90–10 *n*-hexane/*i*PrOH, 0.5 mL/min, t_r_ (major) = 20.1 min, t_r_ (minor) = 24.6 min]; [α] _D_^20^ =  + 19.5 (c = 0.78 in CHCl_3_). ^**1**^**H NMR (600 MHz, CDCl**_**3**_**):** δ 8.38 (br s, 1H), 7.56 (d, *J* = 8 Hz, 1H), 7.45 (d, *J* = 8 Hz, 1H), 7.41–7.38 (m, 2H), 7.34 (s, 1H), 7.29–7.27 (m, 2H), 7.17–7.07 (m, 2H), 5.19 (t, *J* = 8.4 Hz, 1H), 5.10–5.05 (m, 1H), 4.97–4.92 (m, 1H); ^**13**^**C NMR (150 MHz, CDCl**_**3**_**):** δ 137.0, 135.2, 130.6, 128.4, 128.3, 126.4, 125.0, 124.4, 123.3, 122.0, 120.5, 119.2, 114.1, 112.0, 79.6, 41.6.

**Compound 4d:** The crude material was purified by flash chromatography on silica gel (Ethyl acetate/Hexane, 40:100) to afford the product in 93% yield as colorless oil. The chromatographed material was determined to be of 89% ee by chiral HPLC analysis [Chiralpak AD-H, 90–10 *n*-hexane/*i*PrOH, 0.5 mL/min, t_r_ (major) = 53.2 min, t_r_ (minor) = 60.7 min]; [α] _D_^20^ =  + 35.1 (c = 0.85 in CHCl_3_). ^**1**^**H NMR (600 MHz, CDCl**_**3**_**):** δ 8.09 (br s, 1H), 7.45–7.43 (m, 2H), 7.34 (d, *J* = 6.3 Hz, 1H), 7.21–7.09 (m, 6H), 5.75 (t, *J* = 6 Hz, 1H), 5.02–4.95 (m, 2H); ^**13**^**C NMR (150 MHz, CDCl**_**3**_**):** δ 136.4, 136.2, 133.7, 130.0, 128.7, 127.0, 126.2, 122.6, 122.5, 121.7, 120.1, 119.0, 113.3, 111.2, 77.8, 37.9. **IR (KBr, cm**^**−1**^**):** 3417, 3059, 2916, 1723, 1549, 1427, 1247, 738.

**Compound 4e:** The crude material was purified by flash chromatography on silica gel (Ethyl acetate/Hexane, 40:100) to afford the product in 96% yield as colorless oil. The chromatographed material was determined to be of 86% ee by chiral HPLC analysis [Chiralpak AD-H, 90–10 *n*-hexane/*i*PrOH, 0.5 mL/min, t_r_ (major) = 21.0 min, t_r_ (minor) = 23.6 min]; [α] _D_^20^ = -9.5 (c = 1.1 in CHCl_3_). ^**1**^**H NMR (600 MHz, CDCl**_**3**_**):** δ 8.25 (br s, 1H), 7.50 (s, 1H), 7.47–7.38 (m, 3H), 7.30 (d, *J* = 8.2 Hz, 1H), 7.25–7.05 (m, 4H), 5.21 (t, *J* = 8 Hz, 1H), 5.10–5.05 (m, 1H), 4.97–4.91 (m, 1H); ^**13**^**C NMR (150 MHz, CDCl**_**3**_**):** δ 142.1, 136.9, 131.37, 131.32, 130.9, 126.9, 126.4, 123.5, 123.3, 122.1, 120.6, 119.2, 114.1, 112.0, 79.6, 41.6.

**Compound 4f:** The crude material was purified by flash chromatography on silica gel (Ethyl acetate/Hexane, 40:100) to afford the product in 96% yield as colorless oil. The chromatographed material was determined to be of 83% ee by chiral HPLC analysis [Chiralpak AD-H, 90–10 *n*-hexane/*i*PrOH, 0.5 mL/min, t_r_ (major) = 18.1 min, t_r_ (minor) = 19.8 min]; [α] _D_^20^ =  + 7.6 (c = 0.97 in CHCl_3_). ^**1**^**H NMR (600 MHz, CDCl**_**3**_**):** δ 8.10 (br s, 1H), 7.62 (d, *J* = 6 Hz, 1H), 7.43 (d, J = 6.1 Hz, 1H), 7.34 (d, J = 12 Hz, 1H), 7.20–7.05 (m, 6H), 5.75–5.72 (m, 1H), 5.0–4.96 (m, 2H); ^**13**^**C NMR (150 MHz, CDCl**_**3**_**):** δ 138.06, 136.5, 133.3, 129.2, 129.1, 127.8, 126.1, 124.5, 123.8, 122.8, 121.9, 120.0, 119.3, 119.0, 113.4, 111.3, 77.88, 40.6. **IR (KBr, cm**^**−1**^**):** 3417, 3058, 1719, 1549, 1427, 1251, 739.

**Compound 4g:** The crude material was purified by flash chromatography on silica gel (Ethyl acetate/Hexane, 40:100) to afford the product in 98% yield as colorless oil. The chromatographed material was determined to be of 84% ee by chiral HPLC analysis [Chiralpak AD-H, 90–10 *n*-hexane/*i*PrOH, 0.5 mL/min, t_r_ (major) = 43.8 min, t_r_ (minor) = 58.2 min]; [α] _D_^20^ =  + 18.5 (c = 0.96 in CHCl_3_). ^**1**^**H NMR (400 MHz, CDCl**_**3**_**):** δ 8.20 (t, *J* = 6 Hz, 1H), 8.18 (br s, 1H), 8.13 (d, *J* = 12 Hz, 1H), 7.71 (d, *J* = 18 Hz, 1H), 7.52 (t, *J* = 6 Hz, 1H), 7.40–7.38 (m, 2H), 7.23(d, *J* = 6 Hz, 1H), 7.11–7.08 (m, 2H), 5.30 (t, *J* = 18 Hz, 1H), 5.13–5.10 (m, 1H), 5.02–4.98 (m, 1H); ^**13**^**C NMR (100 MHz, CDCl**_**3**_**):** δ 149.7, 148.7, 141.6, 134.0, 131.5, 130.0, 127.0, 123.7, 122.6, 121.1, 119.9, 118.4, 112.9, 111.3, 78.8, 41.4. **IR (KBr, cm**^**−1**^**):** 3417, 3064, 1719, 1528, 1424, 1257, 740.

**Compound 4h:** The crude material was purified by flash chromatography on silica gel (Ethyl acetate/Hexane, 40:100) to afford the product in 98% yield as colorless oil. The chromatographed material was determined to be of 91% ee by chiral HPLC analysis [Chiralpak AD-H, 90–10 *n*-hexane/*i*PrOH, 0.5 mL/min, t_r_ (major) = 18.8 min, t_r_ (minor) = 30.5 min]; [α] _D_^20^ =  + 12.8 (c = 0.75 in CHCl_3_). ^**1**^**H NMR (600 MHz, CDCl**_**3**_**):** δ 8.17 (br s, 1H), 7.90 (d, *J* = 12 Hz, 1H), 7.49 (d, *J* = 8 Hz, 1H), 7.44 (d, *J* = 8 Hz, 1H), 7.40 (t, *J* = 9 Hz, 1H), 7.34 (d, *J* = 8 Hz, 1H), 7.30 (d, *J* = 5.2 Hz, 1H), 7.18 (d, *J* = 6, 1H), 7.13 (s, 1H), 7.04 (t, *J* = 12 Hz, 1H), 5.87 (t, *J* = 12 Hz, 1H), 5.14–5.06 (m, 2H); ^**13**^**C NMR (150 MHz, CDCl**_**3**_**):** δ 149.7, 136.3, 133.8, 132.7, 129.7, 128.5, 127.2, 126.2, 125.2, 122.9, 120.2, 114.6, 112.6, 111.8, 78.5, 36.3. **IR (KBr, cm**^**−1**^**):** 3418, 3062, 1722, 1550, 1423, 1250, 740.

**Compound 4i:** The crude material was purified by flash chromatography on silica gel (Ethyl acetate/Hexane, 40:100) to afford the product in 91% yield as colorless oil. The chromatographed material was determined to be of 83% ee by chiral HPLC analysis [Chiralpak AD-H, 90–10 *n*-hexane/*i*PrOH, 0.5 mL/min, t_r_ (major) = 23.08 min, t_r_ (minor) = 26.96 min]; [α] _D_^20^ =  + 6.9 (c = 1.2 in CHCl_3_). ^**1**^**H NMR (600 MHz, CDCl**_**3**_**):** δ 8.20 (br s, 1H), 7.49 (d, *J* = 8 Hz, 1H), 7.38 (d, *J* = 8 Hz, 1H),7.29–7.20 (m, 3H), 7.17–7.15 (m, 2H), 7.11 (d, *J* = 16 Hz, 1H), 7.045 (d, *J* = 2 Hz, 1H), 5.19 (t, *J* = 8 Hz, 1H), 5.10–5.06 (m, 1H), 4.98–4.92 (m, 1H) 2.34 (s, 3H); ^**13**^**C NMR (150 MHz, CDCl**_**3**_**):** δ 137.7, 137.0, 136.6, 130.1, 128.1, 126.6, 123.1, 122.0, 120.4, 119.4, 115.0, 111.8, 80.1, 41.7, 25.8.

**Compound 4j:** The crude material was purified by flash chromatography on silica gel (Ethyl acetate/Hexane, 40:100) to afford the product in 88% yield as colorless oil. The chromatographed material was determined to be of 85% ee by chiral HPLC analysis [Chiralpak AD-H, 90–10 n-hexane/iPrOH, 0.5 mL/min, t_r_ (major) = 73.6 min, t_r_ (minor) = 80.1 min]; [α] _D_^20^ =  + 16.1 (c = 0.64 in CHCl_3_). ^**1**^**H NMR (600 MHz, CDCl**_**3**_**):** δ 8.05 (br s, 1H), 7.43 (d, *J* = 6 Hz, 1H), 7.35 (d, *J* = 9.2 Hz, 1H), 7.25–7.22 (m, 2H), 7.20–7.06 (m, 2H), 7.00 (d, *J* = 2.0 Hz, 1H), 6.85–6.83 (m, 2H), 5.13 (t, *J* = 6 Hz, 1H), 5.06–5.02 (m, 1H), 4.91–4.87 (m, 1H), 3.77 (s, 3H); ^**13**^**C NMR (150 MHz, CDCl**_**3**_**):** δ 158.8, 136.5, 131.2, 128.3, 125.7, 122.6, 121.4, 120.4, 119.7, 115.1, 114.3, 111.1, 79.5, 55.0, 40.9. **IR (KBr, cm**^**−1**^**):** 3377, 3052, 1727, 1547, 1422, 1241, 741.

**Compound 4k:** The crude material was purified by flash chromatography on silica gel (Ethyl acetate/Hexane, 40:100) to afford the product in 90% yield as colorless oil. The chromatographed material was determined to be of 84% ee by chiral HPLC analysis [Chiralpak AD-H, 90–10 *n*-hexane/*i*PrOH, 0.5 mL/min, t_r_ (major) = 28.4 min, t_r_ (minor) = 30.8 min]; [α] _D_^20^ = -9.5 (c = 1.0 in CHCl_3_). ^**1**^**H NMR (600 MHz, CDCl**_**3**_**):** δ 8.08 (br s, 1H), 7.488 (d, *J* = 7.6 Hz, 1H), 7.35 (d, *J* = 8.4 Hz, 1H), 7.26–7.17 (m, 2H), 7.18 (t, *J* = 9 Hz, 1H), 7.08 (t, *J* = 6 Hz, 1H), 7.02 (d, *J* = 5.2 Hz, 1H), 6.94 (d, *J* = 6, 1H), 6.81–6.79 (m, 1H), 5.16 (t, *J* = 12 Hz, 1H), 5.06–5.03 (m, 1H), 4.95–4.91 (m, 1H) 3.76 (s, 3H); ^**13**^**C NMR (150 MHz, CDCl**_**3**_**):** δ 159.9, 140.6, 136.3, 129.7, 125.9, 122.6, 121.6, 120.4, 120.2, 119.7, 114.8, 114.3, 112.4, 111.3, 79.3, 55.0, 41.4. **IR (KBr, cm**^**−1**^**):** 3417, 3055, 1725, 1548, 1427, 1260, 740.

**Compound 4l:** The crude material was purified by flash chromatography on silica gel (Ethyl acetate/Hexane, 40:100) to afford the product in 92% yield as colorless oil. The chromatographed material was determined to be of 82% ee by chiral HPLC analysis [Chiralpak AD-H, 90–10 *n*-hexane/*i*PrOH, 0.5 mL/min, t_r_ (major) = 23.6 min, t_r_ (minor) = 27.2 min]; [α] _D_^20^ = -12.2 (c = 0.98 in CHCl_3_). ^**1**^**H NMR (600 MHz, CDCl**_**3**_**):** 8.28 (d, *J* = 6 Hz, 1H), 8.06 (br s, 1H), 7.90 (d, *J* = 6 Hz, 1H), 7.79 (t, *J* = 6 Hz, 1H), 7.50– 7.57 (m, 2H), 7.44 (d, *J* = 12.0 Hz, 1H), 7.38–7.40 (m, 3H), 7.20 (t, *J* = 6.1 Hz, 1H), 7.06 (t, *J* = 6.0 Hz, 1H), 7.03 (s, 1H), 6.08 (t, *J* = 6 Hz, 1H), 5.07–5.15 (m, 2H); ^**13**^**C NMR (150 MHz, CDCl**_**3**_**):** δ 136.5, 134.8, 134.3, 131.0, 129.2, 128.2, 127.0, 126.7, 126.2, 125.2, 124.7, 123.9, 120.7, 119.7, 114.3, 111.3, 78.4, 37.1. **IR (KBr, cm**^**−1**^**):** 3418, 3053, 2916, 1728, 1547, 1421, 1226, 796.

**Compound 4m:** The crude material was purified by flash chromatography on silica gel (Ethyl acetate/Hexane, 40:100) to afford the product in 84% yield as colorless oil. The chromatographed material was determined to be of 84% ee by chiral HPLC analysis [Chiralpak AD-H, 90–10 *n*-hexane/*i*PrOH, 0.5 mL/min, t_r_ (major) = 72.8 min, t_r_ (minor) = 83.1 min]; [α] _D_^20^ =  + 7.9 (c = 0.94 in CHCl_3_). ^**1**^**H NMR (400 MHz, CDCl**_**3**_**):** δ 8.17 (br s, 1H), 7.60 (d, *J* = 8 Hz, 1H), 7.42–7.40 (m, 2H), 7.28 (d, *J* = 4 Hz, 1H), 7.19–7.15 (m, 2H), 6.33 (t, *J* = 5.2 Hz, 1H), 6.20 (d, *J* = 4.2, 1H), 5.30 (t, *J* = 12.2 Hz, 1H), 5.09 (dd, *J* = 12.0 Hz, 1H), 4.96 (dd, *J* = 12.6 Hz, 1H); ^**13**^**C NMR (100 MHz, CDCl**_**3**_**):** δ 152.6, 142.7, 136.8, 126.2, 123.2, 123.1, 121.0, 120.6, 119.2, 112.1, 112.0, 110.9, 107.9, 36.2.

**Compound 4n:** The crude material was purified by flash chromatography on silica gel (Ethyl acetate/Hexane, 40:100) to afford the product in 88% yield as colorless oil. The chromatographed material was determined to be of 81% ee by chiral HPLC analysis [Chiralpak AD-H, 90–10 *n*-hexane/*i*PrOH, 0.5 mL/min, t_r_ (major) = 40.6 min, t_r_ (minor) = 45.7 min]; [α] _D_^20^ =  + 21.8 (c = 0.47 in CHCl_3_). ^**1**^**H NMR (600 MHz, CDCl**_**3**_**):** δ 8.18 (br s, 1H), 7.41–7.29 (m, 7H), 7.19–716 (m, 1H), 7.14 (s, 1H), 5.16 (t, *J* = 8 Hz, 1H), 5.09–5.04 (m, 1H), 4.98–4.93 (m, 1H); ^**13**^**C NMR (150 MHz, CDCl**_**3**_**):** δ 139.2, 135.3, 129.5, 128.2, 128.1, 127.7, 126.2, 123.6, 123.3, 118.9, 114.7, 112.9, 79.9, 41.8.

**Compound 4o:** The crude material was purified by flash chromatography on silica gel (Ethyl acetate/Hexane, 40:100) to afford the product in 89% yield as colorless oil. The chromatographed material was determined to be of 81% ee by chiral HPLC analysis [Chiralpak AD-H, 90–10 *n*-hexane/*i*PrOH, 0.5 mL/min, t_r_ (major) = 39.7 min, t_r_ (minor) = 42.7 min]; [α] _D_^20^ =  + 24.6 (c = 0.58 in CHCl_3_). ^**1**^**H NMR (600 MHz, CDCl**_**3**_ δ 8.21 (br s, 1H), 7.55–7.02 (m, 8H), 5.13 (t, *J* = 8 Hz, 1H), 5.07–5.01 (m, 1H), 4.95–4.90 (m, 1H); ^**13**^**C NMR (150 MHz, CDCl**_**3**_**):** δ 138.7, 134.5, 131.5, 131.2, 131.1, 129.5, 128.7, 128.3, 126.8, 123.9, 123.3, 118.7, 114.0, 113.0, 79.6, 41.4.

**Compound 4p:** The crude material was purified by flash chromatography on silica gel (Ethyl acetate/Hexane, 40:100) to afford the product in 91% yield as colorless oil. The chromatographed material was determined to be of 83% ee by chiral HPLC analysis [Chiralpak AD-H, 90–10 *n*-hexane/*i*PrOH, 0.5 mL/min, t_r_ (major) = 114.5 min, t_r_ (minor) = 126.3 min]; [α] _D_^20^ =  + 15.5 (c = 0.86 in CHCl_3_). ^**1**^**H NMR (600 MHz, CDCl**_**3**_**):** δ 8.28 (br s, 1H), 7.98–7.96 (dd, *J* = 1.2 Hz, *J* = 9.2 Hz, 1H), 7.55–7.52 (m, 2H), 7.50–7.45 (m, 2H), 7.32–7.30 (m, 1H), 7.23 (d, *J* = 1.6 Hz, 1H), 7.19–7.16 (m, 1H), 5.86 (t, *J* = 7.6 Hz, 1H), 5.19–5.06 (m, 2H), ^**13**^**C NMR (150 MHz, CDCl**_**3**_**):** δ 137.9, 135.2, 133.8, 133.7, 130.2, 129.3, 127.4, 126.5, 125.8, 124.9, 123.9, 118.6, 113.0, 112.9, 78.4, 36.9.

**Compound 4q:** The crude material was purified by flash chromatography on silica gel (Ethyl acetate/Hexane, 40:100) to afford the product in 84% yield as colorless oil. The chromatographed material was determined to be of 79% ee by chiral HPLC analysis [Chiralpak AD-H, 90–10 *n*-hexane/*i*PrOH, 0.5 mL/min, t_r_ (major) = 81.2 min, t_r_ (minor) = 88.2 min]; [α] _D_^20^ =  + 10.1 (c = 0.49 in CHCl_3_). ^**1**^**H NMR (600 MHz, CDCl**_**3**_**):** δ 8.14 (br s, 1H), 7.46 (d, *J* = 7.6 Hz, 1H), 7.39 (d, *J* = 8.4 Hz, 1H), 7.29–7.24 (m, 2H), 7.12–7.06 (m, 2H), 6.89–6.87 (m, 2H), 5.17 (t, *J* = 8 Hz, 1H), 5.10–5.05 (m, 1H), 4.95–4.90 (m, 1H), 3.80 (s, 3H); ^**13**^**C NMR (150 MHz, CDCl**_**3**_**):** δ 159.3, 137.0, 131.6, 129.3, 126.6, 123.1, 121.9, 120.4, 119.5, 115.2, 114.7, 111.8, 80.2, 55.7, 41.3.

**Compound 4r:** The crude material was purified by flash chromatography on silica gel (Ethyl acetate/Hexane, 40:100) to afford the product in 95% yield as colorless oil. The chromatographed material was determined to be of 84% ee by chiral HPLC analysis [Chiralpak AD-H, 90–10 *n*-hexane/*i*PrOH, 0.5 mL/min, t_r_ (major) = 29.6 min, t_r_ (minor) = 43.5 min]; [α] _D_^20^ =  + 15 (c = 0.71 in CHCl_3_). ^**1**^**H NMR (600 MHz, CDCl**_**3**_**):** δ 8.09 (br s, 1H), 7.37–7.26 (m, 6H), 7.03 (s, 1H), 6.90–6.87 (m, 2H), 5.16 (t, *J* = 6 Hz, 1H), 5.07–5.05 (m, 1H), 4.99–4.94 (m, 1H), 3.80 (s, 3H); ^**13**^**C NMR (150 MHz, CDCl**_**3**_**):** δ 154.6, 139.6, 132.0, 129.4, 128.2, 128.0, 127.0, 122.7, 114.5, 113.2, 112.6, 101.3, 80.0, 56.3, 42.0. **IR (KBr, cm**^**−1**^**):** 3422, 2944, 1727, 1548, 1449, 1259, 747.

**Compound 4s:** The crude material was purified by flash chromatography on silica gel (Ethyl acetate/Hexane, 40:100) to afford the product in 95% yield as colorless oil. The chromatographed material was determined to be of 89% ee by chiral HPLC analysis [Chiralpak AD-H, 90–10 *n*-hexane/*i*PrOH, 0.5 mL/min, t_r_ (major) = 27.4 min, t_r_ (minor) = 33.3 min]; [α] _D_^20^ =  + 21 (c = 1.1 in CHCl_3_). ^**1**^**H NMR (600 MHz, CDCl**_**3**_ δ 8.14 (br s, 1H), 7.50 (s, 1H), 7.42 (d, *J* = 7.6 Hz, 1H), 7.30–7.20 (m, 3H), 7.02 (d, *J* = 2 Hz, 1H), 6.92–6.86 (m, 2H), 5.13 (t, *J* = 8 Hz, 1H), 5.07–5.02 (m, 1H), 4.95–4.90 (m, 1H), 3.82 (s, 3H); ^**13**^**C NMR (150 MHz, CDCl**_**3**_**):** δ 154.7, 142.1, 132.09, 131.36, 131.31, 131.0, 126.9, 126.8, 123.5, 122.8, 113.7, 113.3, 112.7, 101.1, 79.6, 56.4, 41.6.

**Compound 4t:** The crude material was purified by flash chromatography on silica gel (Ethyl acetate/Hexane, 40:100) to afford the product in 98% yield as colorless oil. The chromatographed material was determined to be of 88% ee by chiral HPLC analysis [Chiralpak AD-H, 90–10 *n*-hexane/*i*PrOH, 0.5 mL/min, t_r_ (major) = 65.4 min, t_r_ (minor) = 90.3 min]; [α] _D_^20^ =  + 12.6 (c = 0.89 in CHCl_3_). ^**1**^**H NMR (600 MHz, CDCl**_**3**_**):** δ 8.15 (br s, 1H), 7.92 (d, *J* = 8 Hz, 1H), 7.56–7.52 (m, 1H), 7.46–7.42 (m, 2H), 7.29–7.25 (m, 1H), 7.15 (s, 2H), 6.88–6.85 (m, 1H), 6.79 (d, *J* = 2 Hz, 1H), 5.84 (t, *J* = 7.6 Hz, 1H), 5.12–5.09 (m, 2H), 3.77 (s, 3H); ^**13**^**C NMR (150 MHz, CDCl**_**3**_**):** δ 154.8, 150.2, 134.1, 133.6, 131.9, 130.3, 129.0, 126.8, 125.4, 122.9, 113.6, 113.0, 112.6, 100.8, 78.6, 56.2, 36.7.

**Compound 4u:** The crude material was purified by flash chromatography on silica gel (Ethyl acetate/Hexane, 40:100) to afford the product in 91% yield as colorless oil. The chromatographed material was determined to be of 83% ee by chiral HPLC analysis [Chiralpak AD-H, 90–10 *n*-hexane/*i*PrOH, 0.5 mL/min, t_r_ (major) = 46.9 min, t_r_ (minor) = 63.5 min]; [α] _D_^20^ =  + 7.8 (c = 0.93 in CHCl_3_). ^**1**^**H NMR (600 MHz, CDCl**_**3**_ δ 8.05 (br s, 1H), 7.29–7.26 (m, 3H), 7.02 (d, *J* = 3 Hz, 1H), 6.89–6.87 (m, 4H), 5.13–5.03 (m, 2H), 4.94–4.89 (m, 1H), 3.81–3.80 (s, 6H); ^**13**^**C NMR (150 MHz, CDCl**_**3**_**):** δ 159.3, 154.6, 132.1, 131.6, 129.3, 127.0, 122.6, 114.9, 114.7, 113.1, 112.5, 101.4, 80.21, 56.3, 55.7, 41.3 (Supplementary figures).

### Supplementary Information

Supplementary Figures.

## Data Availability

The datasets generated during and/or analysed during the current study are available from the corresponding author on reasonable request.
