# Supplementary material for: Catalytic asymmetric Friedel–Crafts alkylation of unprotected indoles with nitroalkenes using a novel chiral Yb(OTf)3–pybox complex
Source: Sci Rep. 2023 Sep 7;13:14736. doi: 10.1038/s41598-023-41921-9 (PMC10484919; doi:10.1038/s41598-023-41921-9)
Supplement: Supplementary file 1 — Supplementary Figures. [file 41598_2023_41921_MOESM1_ESM.pdf]

Catalytic Asymmetric Friedel-Crafts Alkylation of unprotected Indoles with Nitroalkenes Using a Novel Chiral $Yb(OTf)_3$-Pybox Complex

Babak karimi,* Ehsan Jafari, Fariborz Mansouri and Mina Tavakolian

Department of Chemistry
Institute for Advanced Studies in Basic Sciences (IASBS)
PO-Box 45195-1159, Prof. Sobouti Boulevard, Zanjan 45137-66731, Iran.
Fax: +98-24-3341-4949; Tel: +98-24-3315-3225

E-mail: karimi@iasbs.ac.ir

Table of Content

Title	Page
Figure S1: HPLC chromatogram for racemic mixture of **4a**	S5
Figure S2: HPLC chromatogram for chiral **4a**	S5
Figure S3: ^{1}HNMR spectrum of compound **4a**	S6
Figure S4: ^{13}CNMR spectrum of compound **4a**	S6
Figure S5: HPLC chromatogram for racemic mixture of **4b**	S7
Figure S6: HPLC chromatogram for chiral **4b**	S7
Figure S7: ^{1}HNMR spectrum of compound **4b**	S8
Figure S8: ^{13}CNMR spectrum of compound **4b**	S8
Figure S9: FTIR spectrum of compound **4b**	S9
Figure S10: HPLC chromatogram for racemic mixture of **4c**	S9
Figure S11: HPLC chromatogram for chiral **4c**	S10
Figure S12: ^{1}HNMR spectrum of compound **4c** (Purity of product is 91% according to ^{1}HNMR)	S10
Figure S13: ^{13}CNMR spectrum of compound **4c**	S11
Figure S14: HPLC chromatogram for racemic mixture of **4d**	S11
Figure S15: HPLC chromatogram for chiral **4d**	S12
Figure S16: ^{1}HNMR spectrum of compound **4d**	S12
Figure S17: ^{13}CNMR spectrum of compound **4d**	S13
Figure S18: FTIR spectrum of compound **4d**	S13
Figure S19: HPLC chromatogram for racemic mixture of **4e**	S14
Figure S20: HPLC chromatogram for chiral **4e**	S14
Figure S21: ^{1}HNMR spectrum of compound **4e** (purity of product is 95% according to ^{1}HNMR)	S15
Figure S22: ^{13}CNMR spectrum of compound **4e**	S15
Figure S23: HPLC chromatogram for racemic mixture of **4f**	S16
Figure S24: HPLC chromatogram for chiral **4f**	S16
Figure S25: ^{1}HNMR spectrum of compound **4f** (purity of product is 92% according to ^{1}HNMR)	S17
Figure S26: ^{13}CNMR spectrum of compound **4f**	S17
Figure S27: FTIR spectrum of compound **4f**	S18
Figure S28: HPLC chromatogram for racemic mixture of **4g**	S18
Figure S29: HPLC chromatogram for chiral **4g**	S19
Figure S30: ^{1}HNMR spectrum of compound **4g**	S19
Figure S31: ^{13}CNMR spectrum of compound **4g**	S20
Figure S32: FTIR spectrum of compound **4g**	S20
Figure S33: HPLC chromatogram for racemic mixture of **4h**	S21
Figure S34: HPLC chromatogram for chiral **4h**	S21
Figure S35: ^{1}HNMR spectrum of compound **4h** (purity of product is 97% according to ^{1}HNMR)	S22
Figure S36: ^{13}CNMR spectrum of compound **4h**	S22
Figure S37: FTIR spectrum of compound **4h**	S23
Figure S38: HPLC chromatogram for racemic mixture of **4i**	S23
Figure S39: HPLC chromatogram for chiral **4i**	S24
Figure S40: ^{1}HNMR spectrum of compound **4i** (purity of product is 98% according to ^{1}HNMR)	S24
Figure S41: ^{13}CNMR spectrum of compound **4i**	S25

Figure S42: HPLC chromatogram for racemic mixture of **4j**	S25
Figure S43: HPLC chromatogram for chiral **4j**	S26
Figure S44: ^{1}HNMR spectrum of compound **4j**	S26
Figure S45: ^{13}CNMR spectrum of compound **4j**	S27
Figure S46: FTIR spectrum of compound **4j**	S27
Figure S47: HPLC chromatogram for racemic mixture of **4k**	S28
Figure S48: HPLC chromatogram for chiral **4k**	S28
Figure S49: ^{1}HNMR spectrum of compound **4k**	S29
Figure S50: ^{13}CNMR spectrum of compound **4k**	S29
Figure S51: FTIR spectrum of compound **4k**	S30
Figure S52: HPLC chromatogram for racemic mixture of **4l**	S30
Figure S53: HPLC chromatogram for chiral **4l**	S31
Figure S54: ^{1}HNMR spectrum of compound **4l**	S31
Figure S55: ^{13}CNMR spectrum of compound **4l**	S32
Figure S56: FTIR spectrum of compound **4l**	S32
Figure S57: HPLC chromatogram for racemic mixture of **4m**	S33
Figure S58: HPLC chromatogram for chiral **4m**	S33
Figure S59: ^{1}HNMR spectrum of compound **4m** (purity of product is 96% according to ^{1}HNMR)	S34
Figure S60: ^{13}CNMR spectrum of compound **4m**	S34
Figure S61: HPLC chromatogram for racemic mixture of **4n**	S35
Figure S62: HPLC chromatogram for chiral **4n**	S35
Figure S63: ^{1}HNMR spectrum of compound **4n**	S36
Figure S64: ^{13}CNMR spectrum of compound **4n**	S36
Figure S65: HPLC chromatogram for racemic mixture of **4o**	S37
Figure S66: HPLC chromatogram for chiral **4o**	S37
Figure S67: ^{1}HNMR spectrum of compound **4o**	S38
Figure S68: ^{13}CNMR spectrum of compound **4o**	S38
Figure S69: HPLC chromatogram for racemic mixture of **4p**	S39
Figure S70: HPLC chromatogram for chiral **4p**	S39
Figure S71: ^{1}HNMR spectrum of compound **4p**	S40
Figure S72: ^{13}CNMR spectrum of compound **4p**	S40
Figure S73: HPLC chromatogram for racemic mixture of **4q**	S41
Figure S74: HPLC chromatogram for chiral **4q**	S41
Figure S75: ^{1}HNMR spectrum of compound **4q** (purity of product is 82% according to ^{1}HNMR)	S42
Figure S76: ^{13}CNMR spectrum of compound **4q**	S42
Figure S77: HPLC chromatogram for racemic mixture of **4r**	S43
Figure S78: HPLC chromatogram for chiral **4r**	S43
Figure S79: ^{1}HNMR spectrum of compound **4r**	S44
Figure S80: ^{13}CNMR spectrum of compound **4r**	S44
Figure S81: FTIR spectrum of compound **4r**	S45
Figure S82: HPLC chromatogram for racemic mixture of **4s**	S45
Figure S83: HPLC chromatogram for chiral **4s**	S46
Figure S84: ^{1}HNMR spectrum of compound **4s**	S46
Figure S85: ^{13}CNMR spectrum of compound **4s**	S47
Figure S86: HPLC chromatogram for racemic mixture of **4t**	S47
Figure S87: HPLC chromatogram for chiral **4t**	S48
Figure S88: ^{1}HNMR spectrum of compound **4t**	S48
Figure S89: ^{13}CNMR spectrum of compound **4t**	S49
Figure S90: HPLC chromatogram for racemic mixture of **4u**	S49
Figure S91: HPLC chromatogram for chiral **4u**	S50
Figure S92: ^{1}HNMR spectrum of compound **4u**	S50

Figure S93: ^{13}CNMR spectrum of compound **4u**	S51
Figure S94: ^{1}HNMR spectrum of compound **8**	S51
Figure S95: ^{13}CNMR spectrum of compound **8**	S52
Figure S96: ^{1}HNMR spectrum of compound **4-Cl-Indeno pybox 1g**	S52
Figure S97: ^{13}CNMR spectrum of compound **4-Cl-Indeno pybox 1g**	S53
Figure S98: MS spectrum of compound **4-Cl-Indeno pybox 1g**	S53
Figure S99: FTIR spectrum of compound **4-Cl-Indeno pybox 1g**	S54

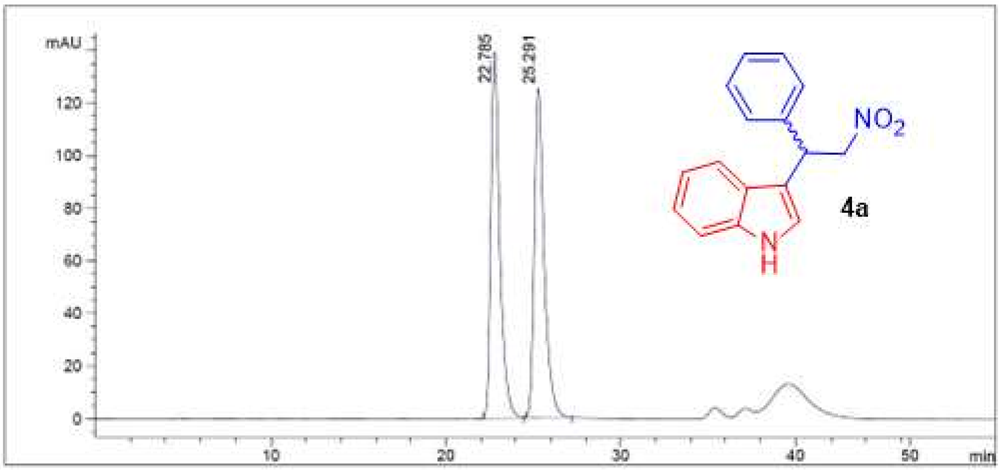

#	Ret. Time (min)	Width	Height (mAU)	Area (mAU*s)	Area %
1	22.78	0.53	139.61	4955.43	50.02
2	25.29	0.59	125.42	4951.48	49.98
Total				9906.91	100.00

Figure S1: HPLC chromatogram for racemic mixture of **4a**

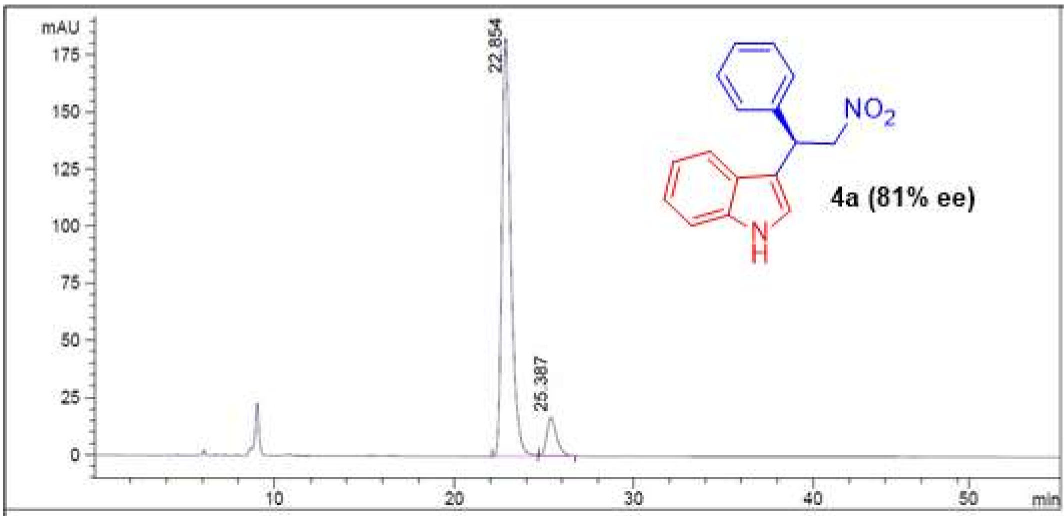

#	Ret. Time (min)	Width	Height (mAU)	Area (mAU*s)	Area %
1	22.85	0.52	183.27	6373.77	90.58
2	25.39	0.57	17.05	662.80	9.42
Total				7036.57	100.00

Figure S2: HPLC chromatogram for chiral **4a**

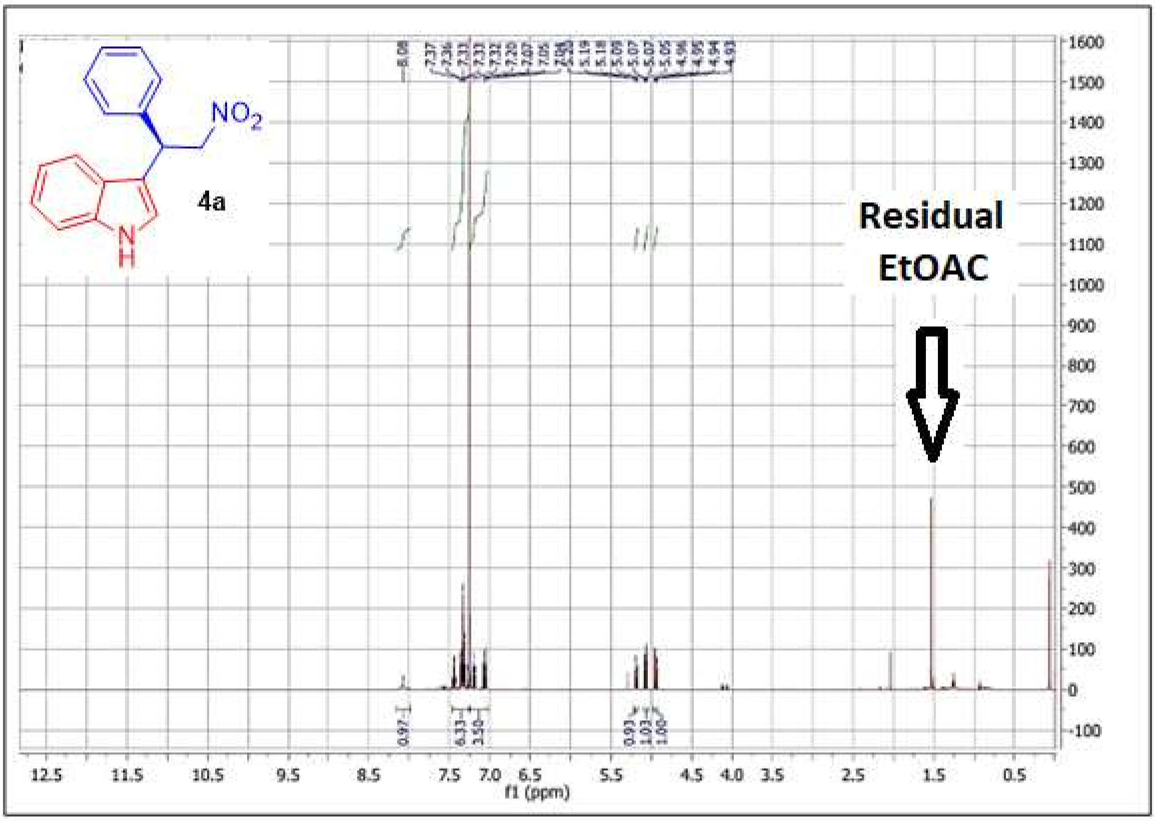

Figure S3: ^{1}HNMR spectrum of compound **4a**

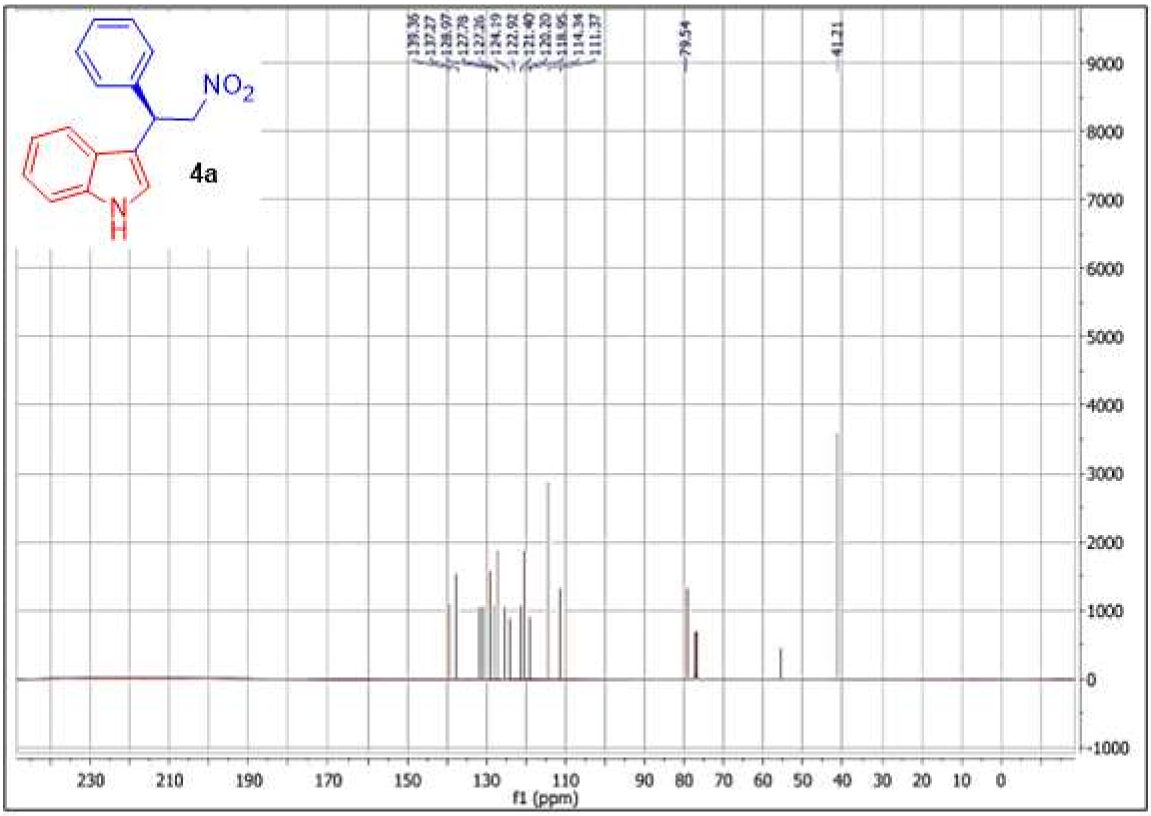

Figure S4: ^{13}CNMR spectrum of compound **4a**

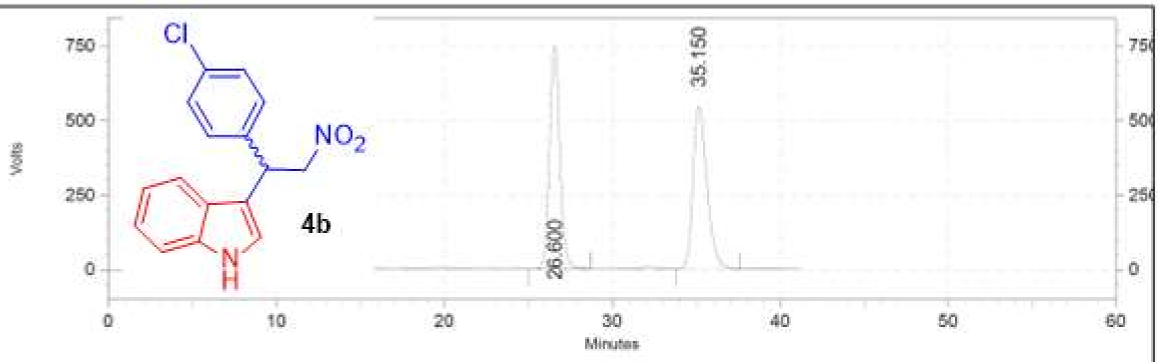

UV [1] Results

Retention Time	Area	Area %	Height	Height %
26.600	31804924	50.01	746991	57.87
35.150	31793425	49.99	543773	42.13
Totals	63598349	100.00	1290764	100.00

Figure S5: HPLC chromatogram for racemic mixture of **4b**

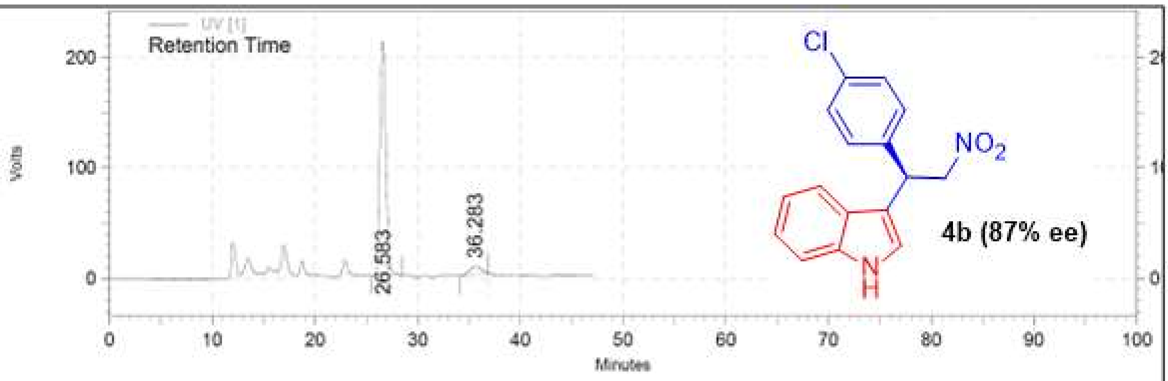

UV [1] Results

Retention Time	Area	Area %	Height	Height %
26.583	8424304	93.88	212310	96.70
36.283	549252	6.12	7243	3.30
Totals	8973556	100.00	219553	100.00

Figure S6: HPLC chromatogram for chiral **4b**

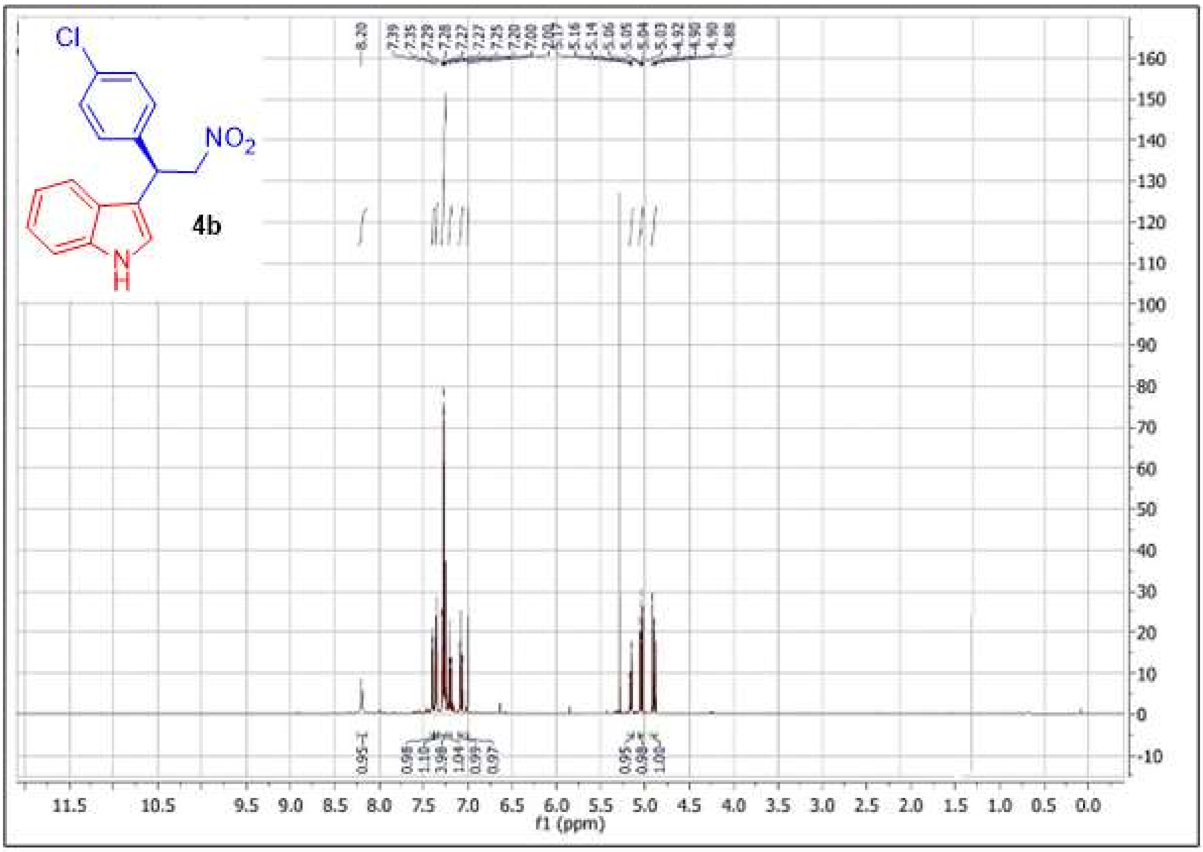

Figure S7: ^{1}HNMR spectrum of compound **4b**

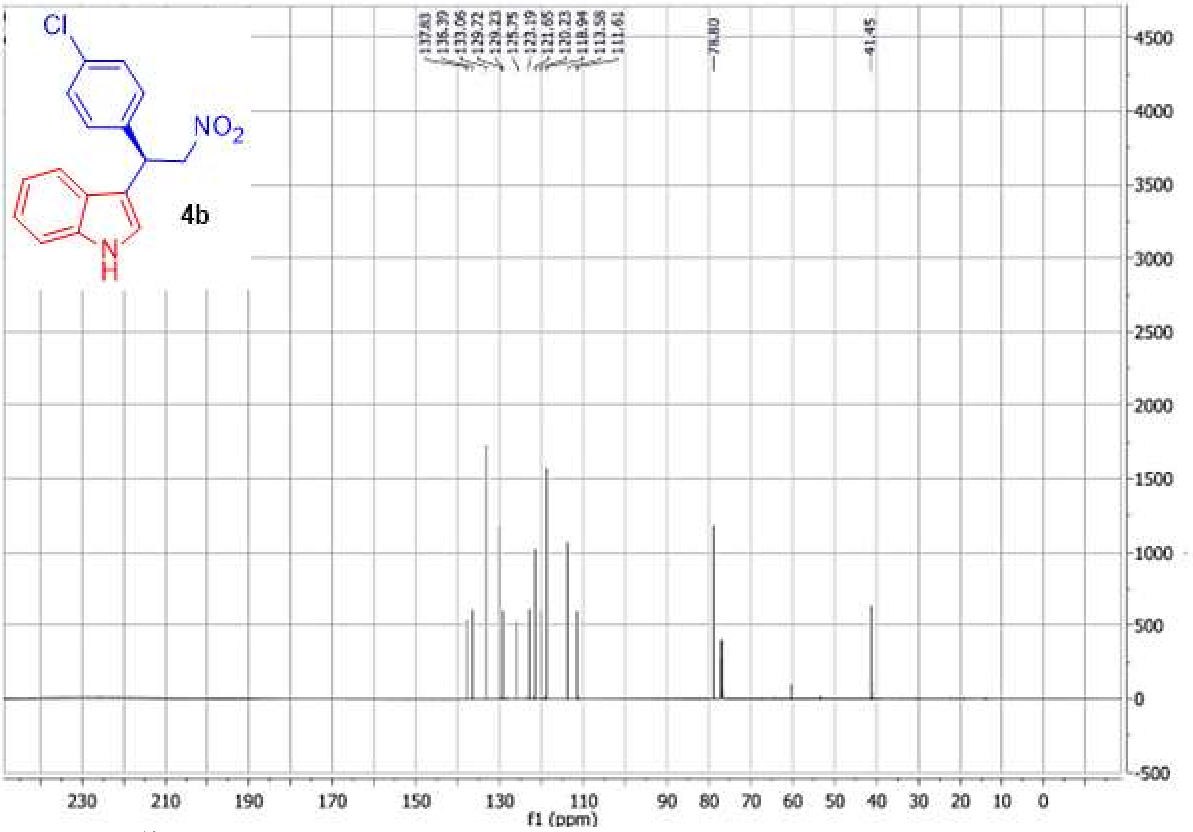

Figure S8: ^{13}CNMR spectrum of compound **4b**

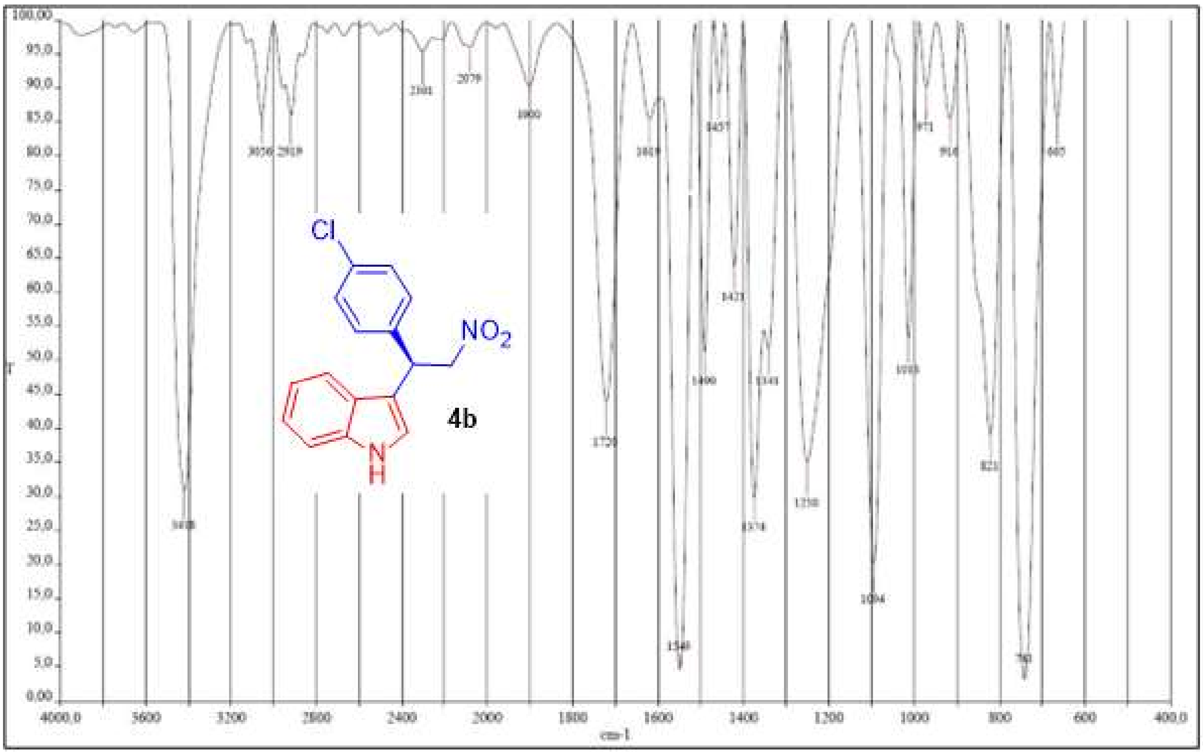

Figure S9: FTIR spectrum of compound **4b**

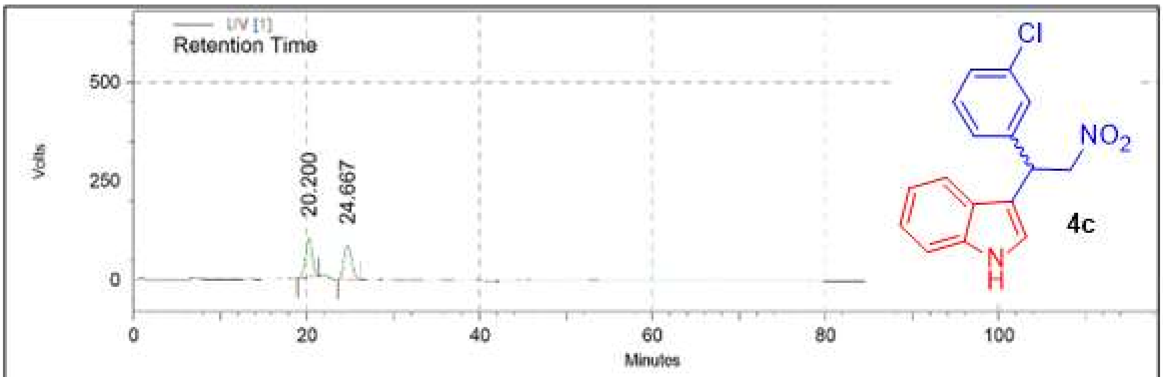

UV [1] Results

Retention Time	Area	Area %	Height	Height %
20.200	5258166	49.39	99031	53.41
24.667	5388983	50.61	86388	46.59
Totals	10647149	100.00	185419	100.00

Figure S10: HPLC chromatogram for racemic mixture of **4c**

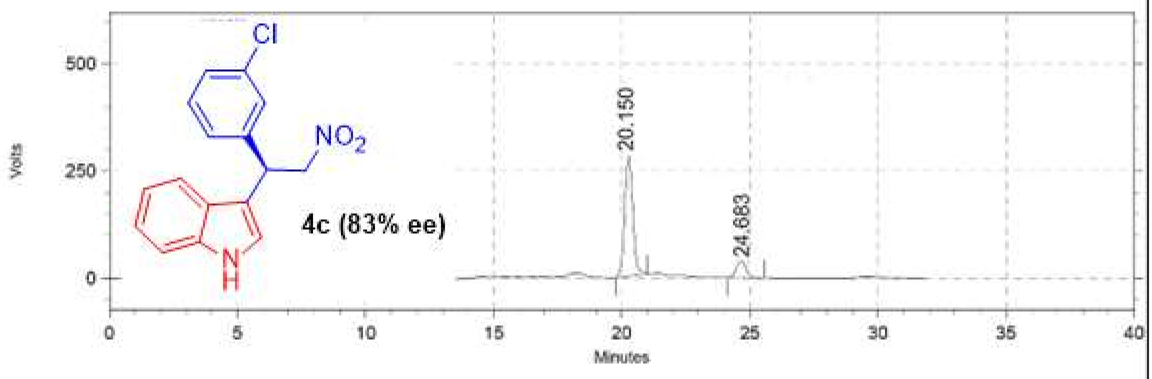

UV [1] Results

Retention Time	Area	Area %	Height	Height %
20.150	2180114	91.90	282773	82.07
24.683	446422	8.10	48883	17.93
Totals	2626536	100.00	52365	100.00

Figure S11: HPLC chromatogram for chiral **4c**

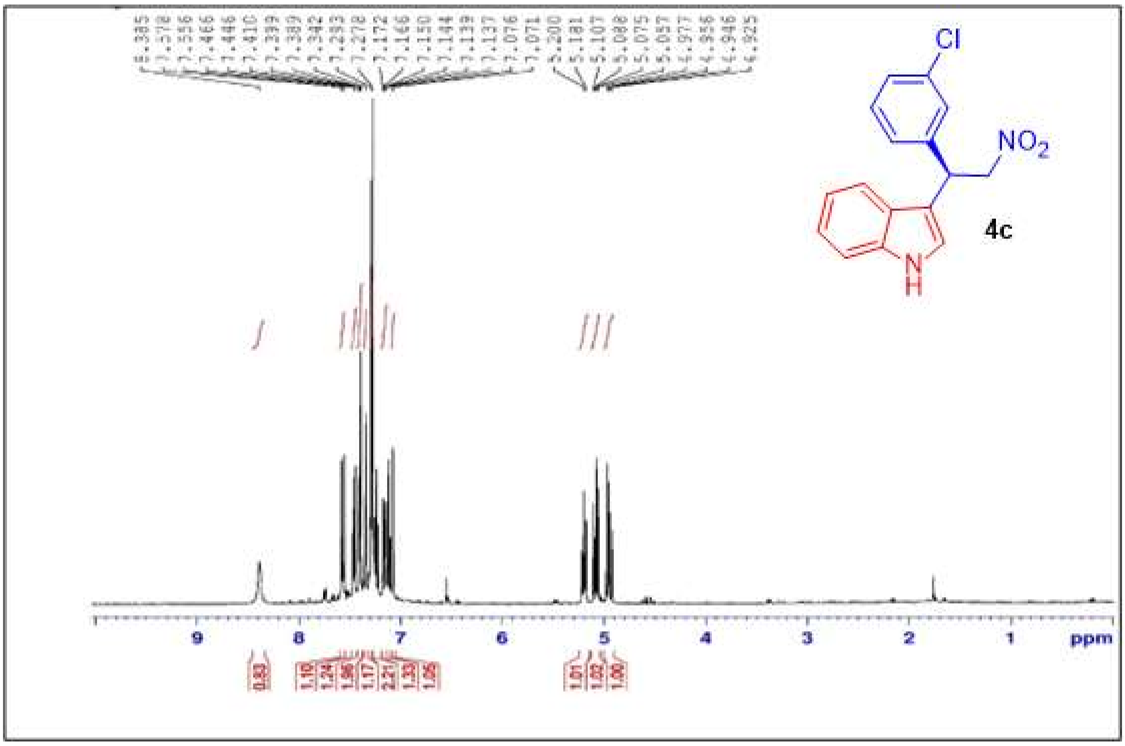

Figure S12: ^{1}HNMR spectrum of compound **4c** (Purity of product is 91% according to ^{1}HNMR)

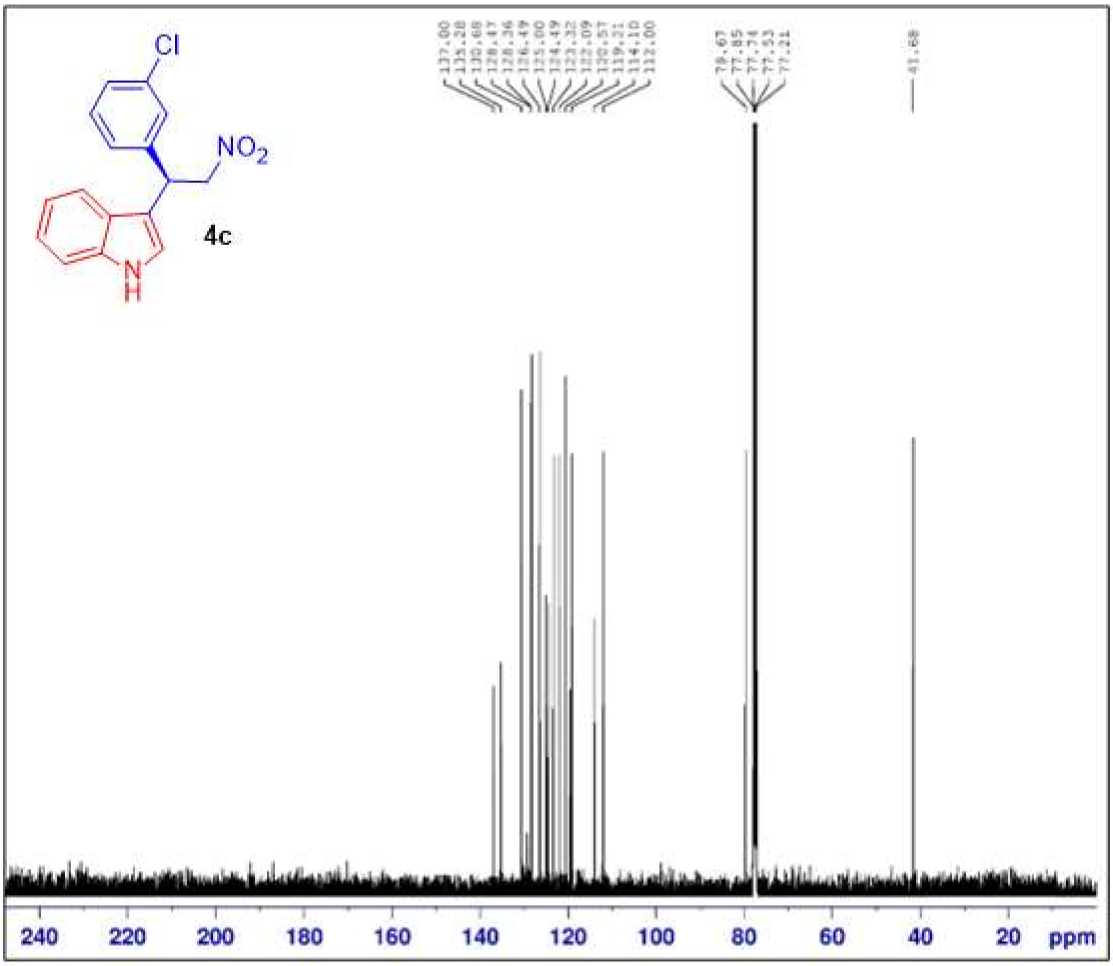

Figure S13: ^{13}CNMR spectrum of compound **4c**

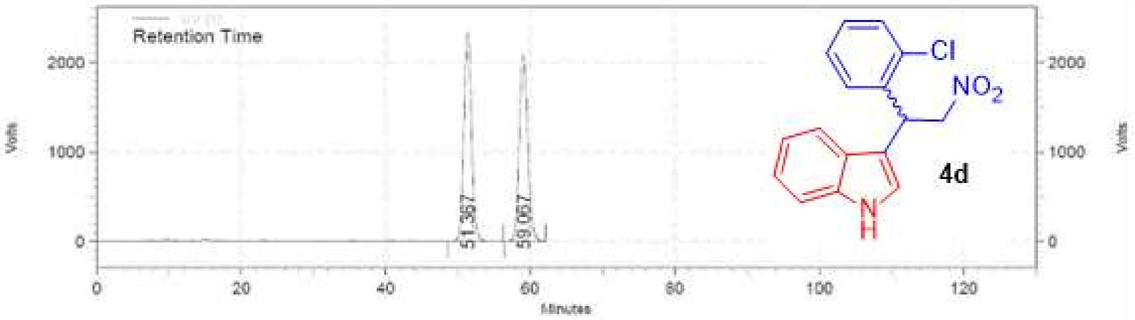

UV [1] Results

Retention Time	Area	Area %	Height	Height %
51.367	167590859	49.74	2323214	52.82
59.067	169357652	50.26	2075113	47.18
Totals	336948511	100.00	4398327	100.00

Figure S14: HPLC chromatogram for racemic mixture of **4d**

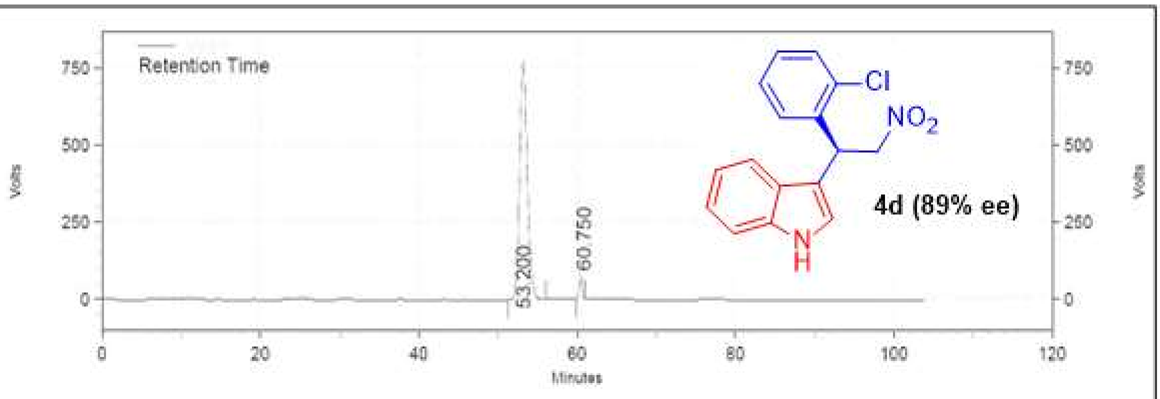

UV [1] Results

Retention Time	Area	Area %	Height	Height %
53.200	51713112	94.89	771811	94.46
60.750	2786817	5.11	4526	5.54
Totals	54499929	100.00	817080	100.00

Figure S15: HPLC chromatogram for chiral **4d**

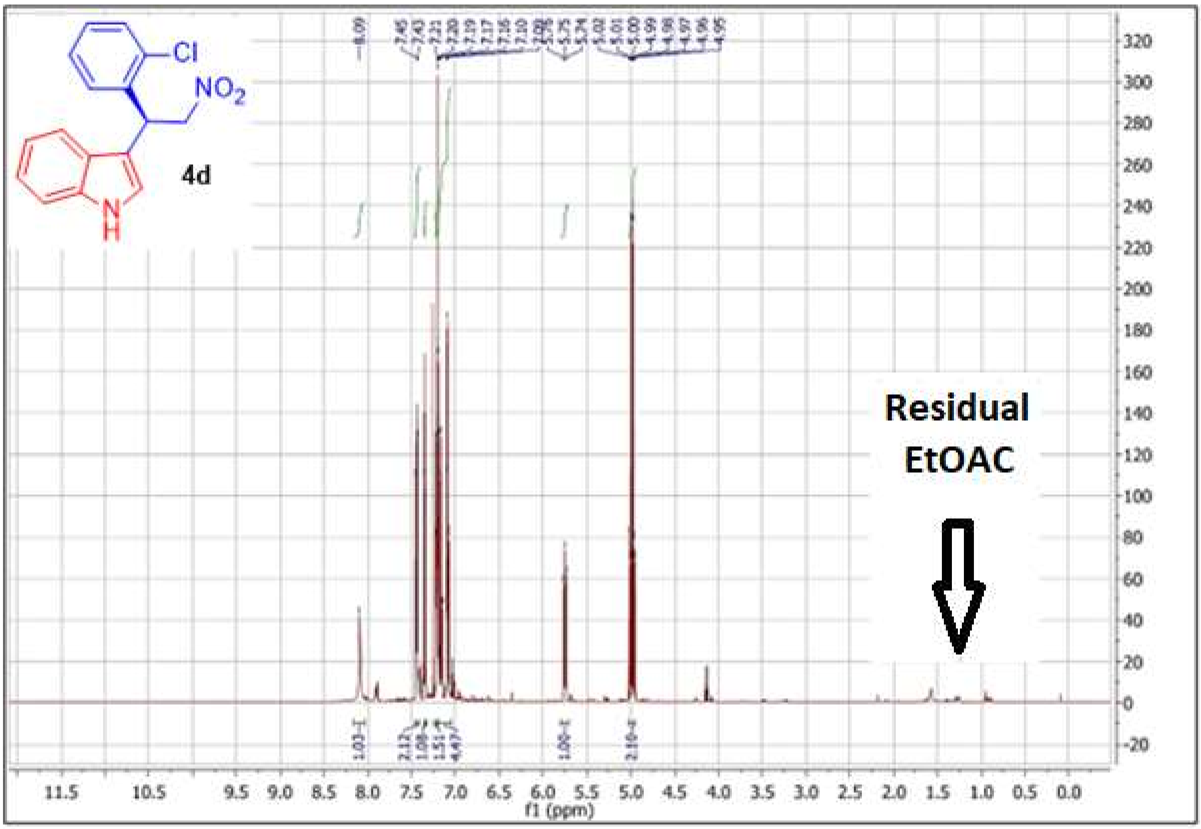

Figure S16: ^{1}HNMR spectrum of compound **4d**

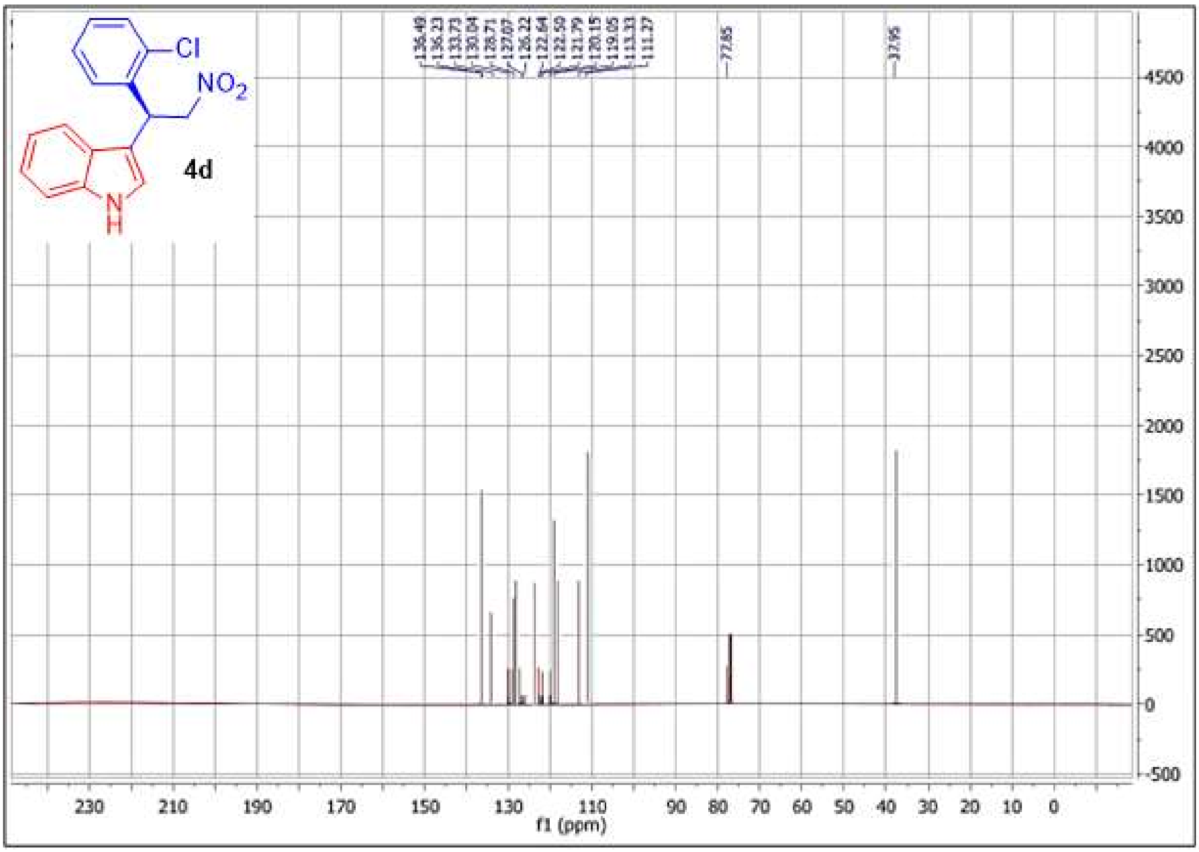

Figure S17: ^{13}CNMR spectrum of compound **4d**

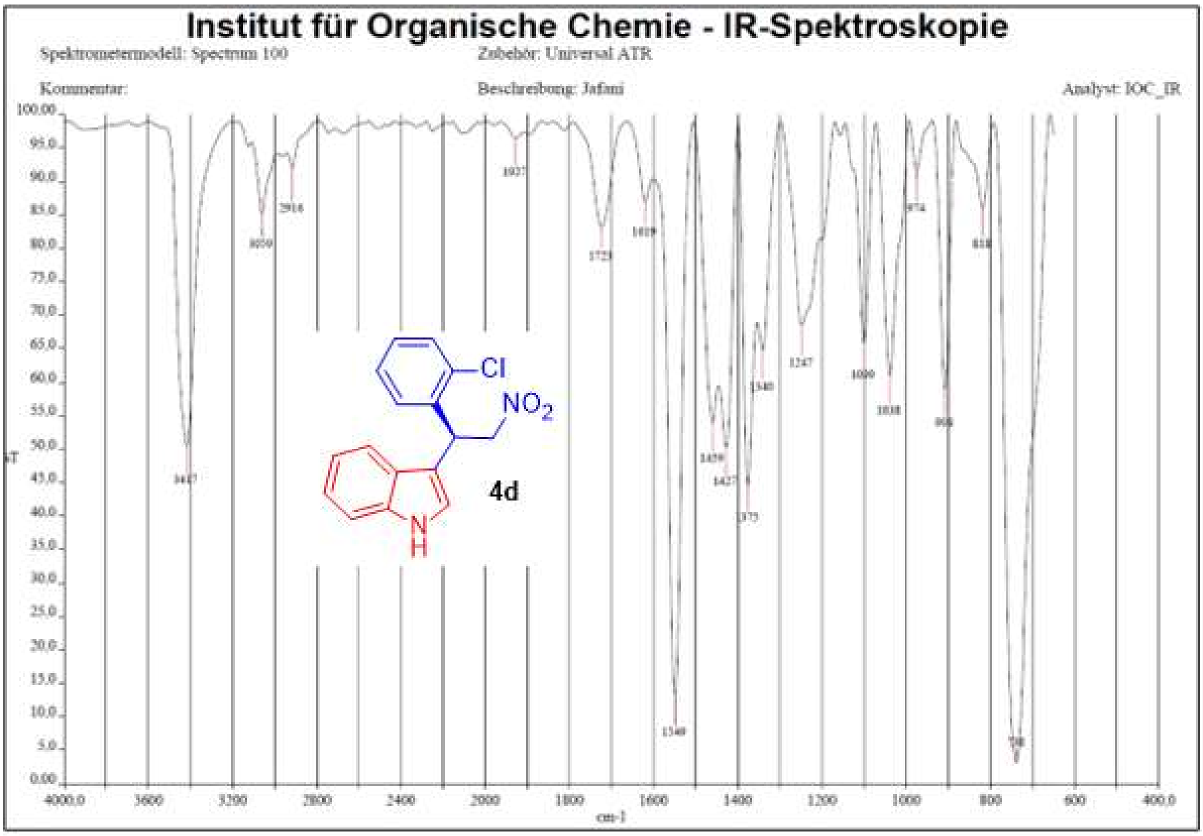

Figure S18: FTIR spectrum of compound **4d**

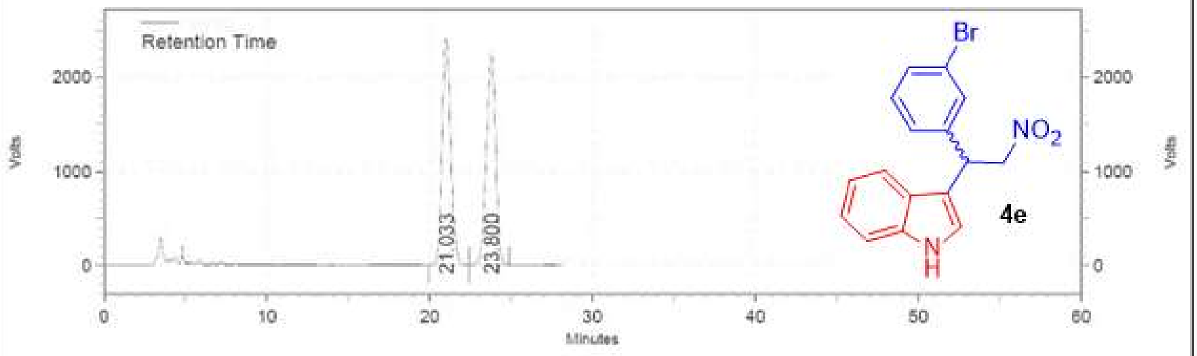

UV [1] Results

Retention Time	Area	Area %	Height	Height %
21.033	87124846	50.01	2407762	51.94
23.800	87089964	49.99	2228004	48.06
Totals	174214810	100.00	4635766	100.00

Figure S19: HPLC chromatogram for racemic mixture of **4e**

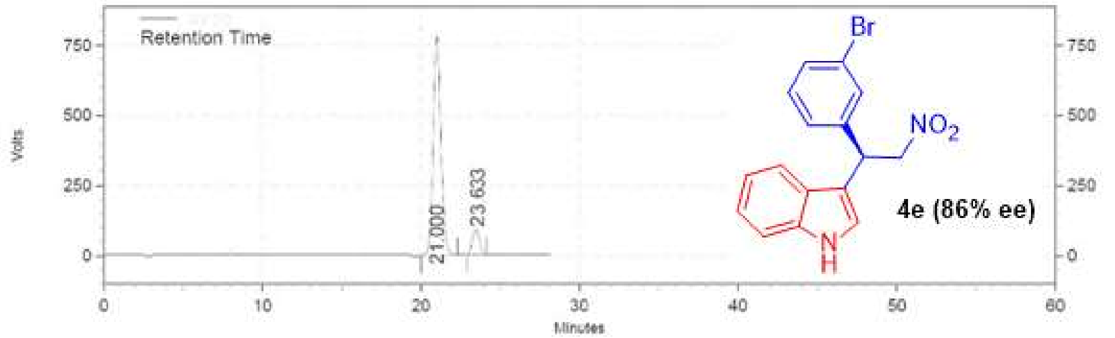

UV [1] Results

Retention Time	Area	Area %	Height	Height %
21.000	26478480	93.04	789017	85.85
23.633	1981215	6.96	130071	14.15
Totals	28459695	100.00	919088	100.00

Figure S20: HPLC chromatogram for chiral **4e**

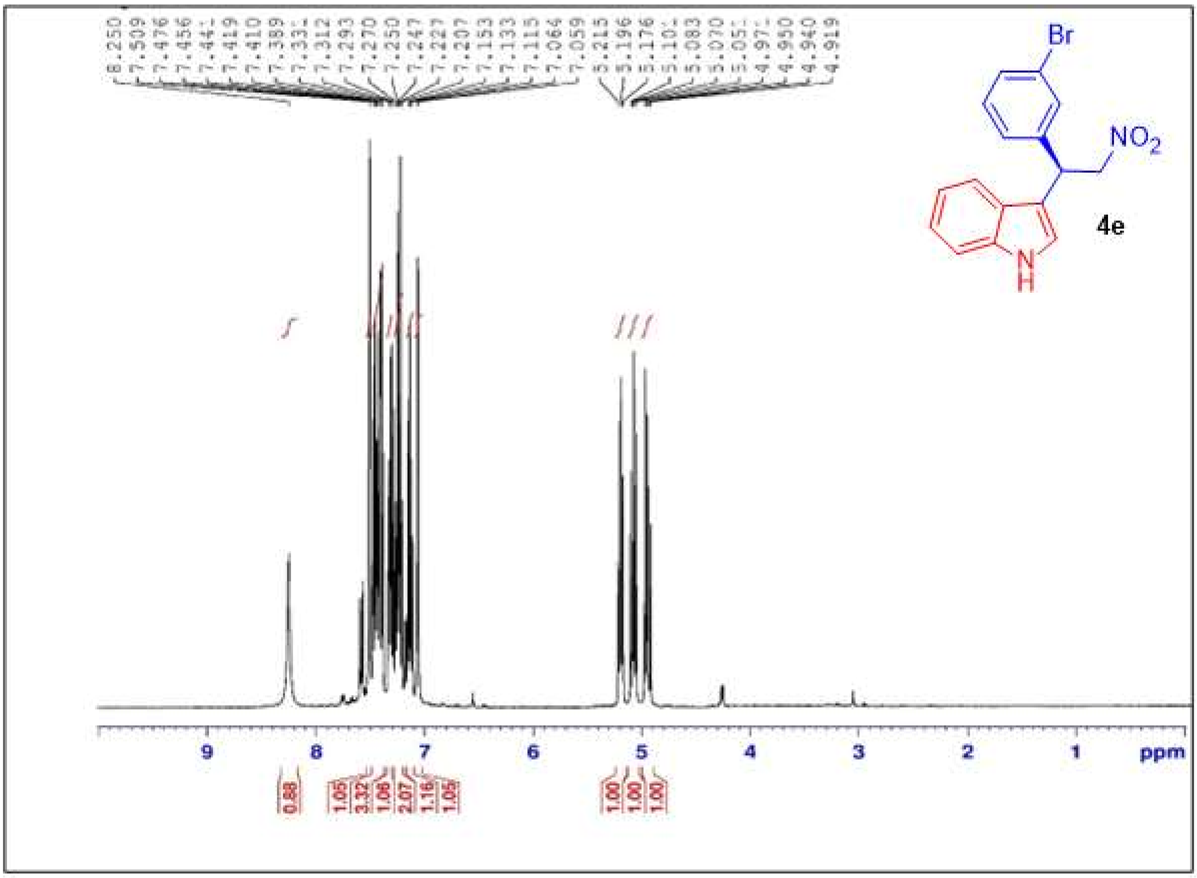

Figure S21: ^{1}HNMR spectrum of compound **4e** (purity of product is 95% according to ^{1}HNMR)

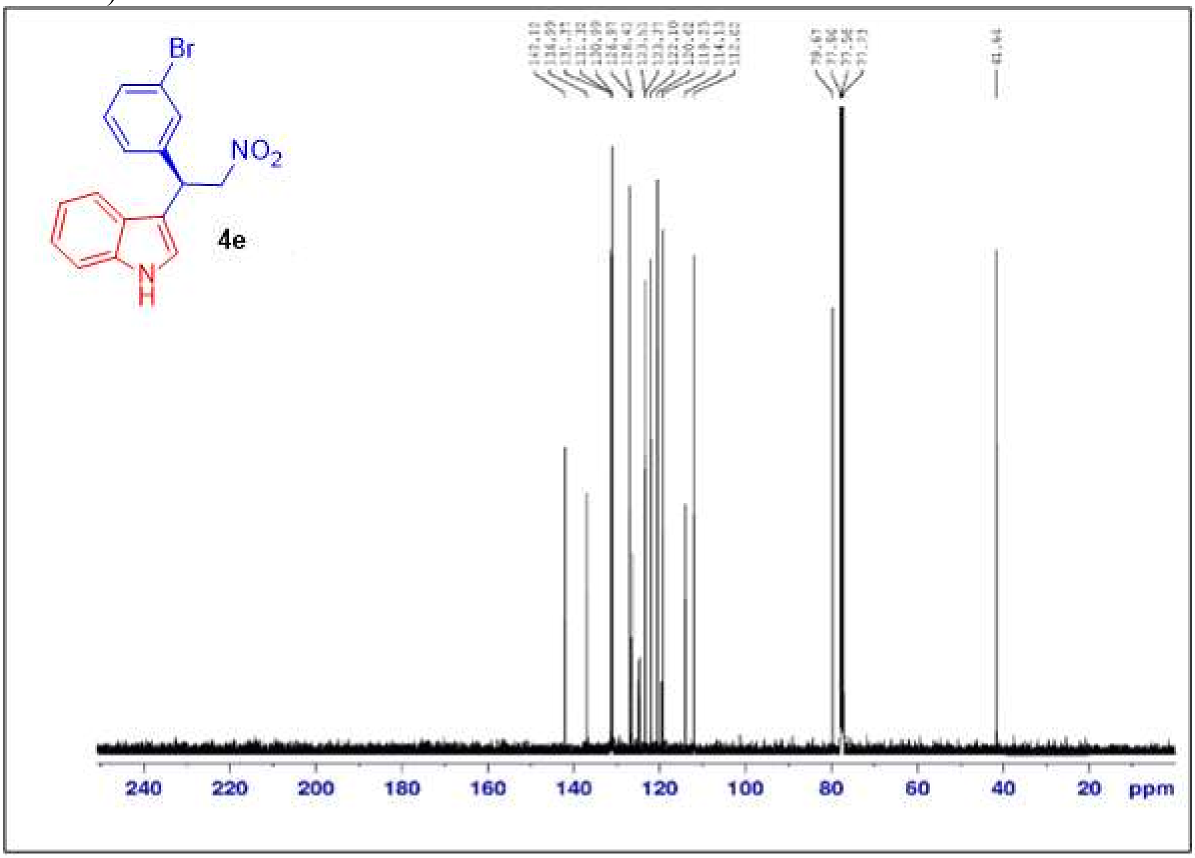

Figure S22: ^{13}CNMR spectrum of compound **4e**

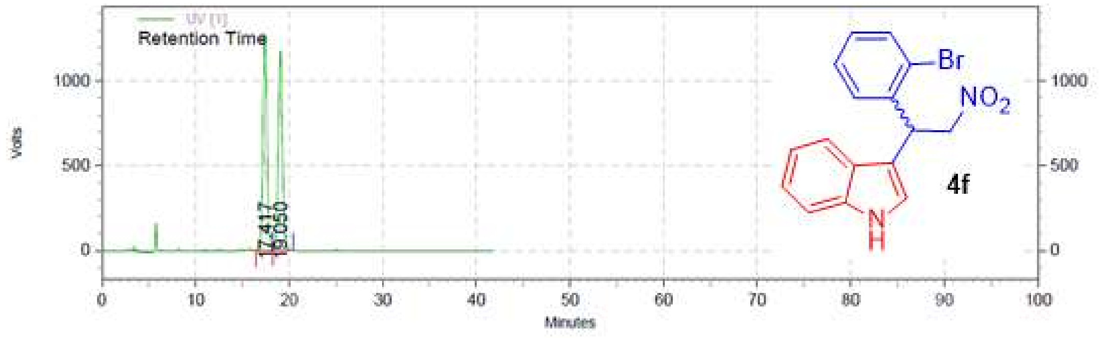

UV [1] Results

Retention Time	Area	Area %	Height	Height %
17.417	36746171	49.89	1275175	51.97
19.050	36912607	50.11	1178521	48.03
Totals	73658778	100.00	2453696	100.00

Figure S23: HPLC chromatogram for racemic mixture of **4f**

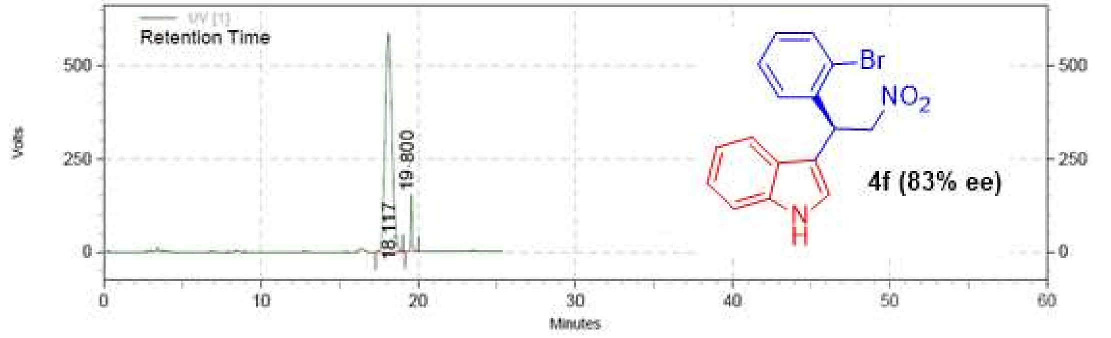

UV [1] Results

Retention Time	Area	Area %	Height	Height %
18.117	17471881	91.78	585885	82.66
19.800	1563881	8.22	122882	17.34
Totals	19035762	100.00	708767	100.00

Figure S24: HPLC chromatogram for chiral **4f**

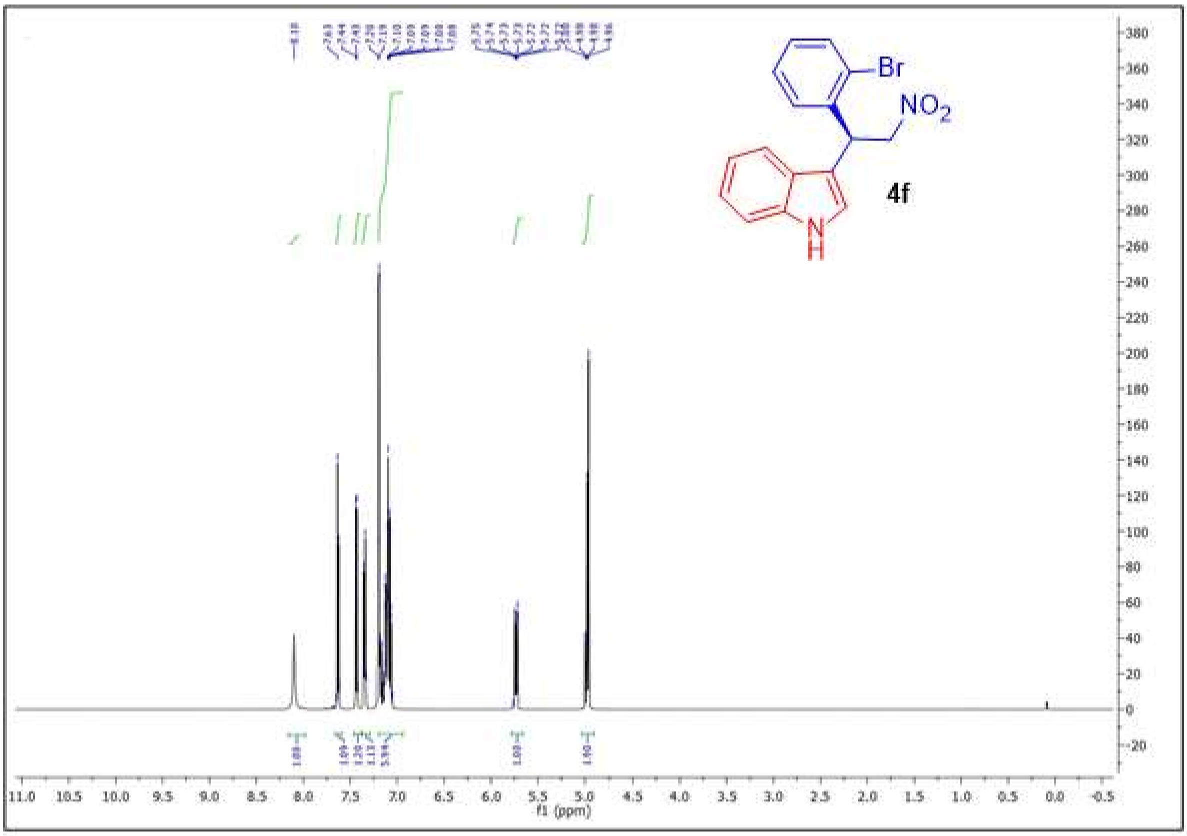

Figure S25: ^{1}HNMR spectrum of compound **4f** (purity of product is 92% according to ^{1}HNMR)

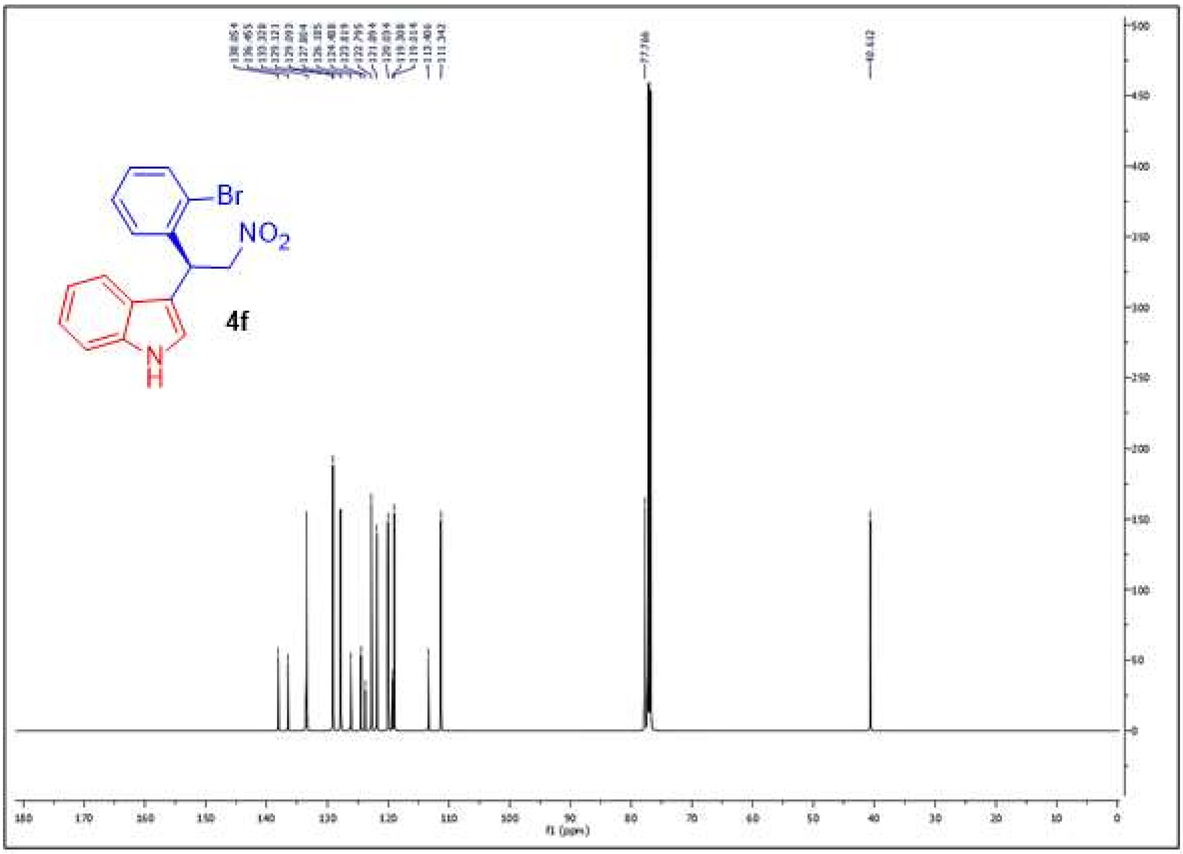

Figure S26: ^{13}CNMR spectrum of compound **4f**

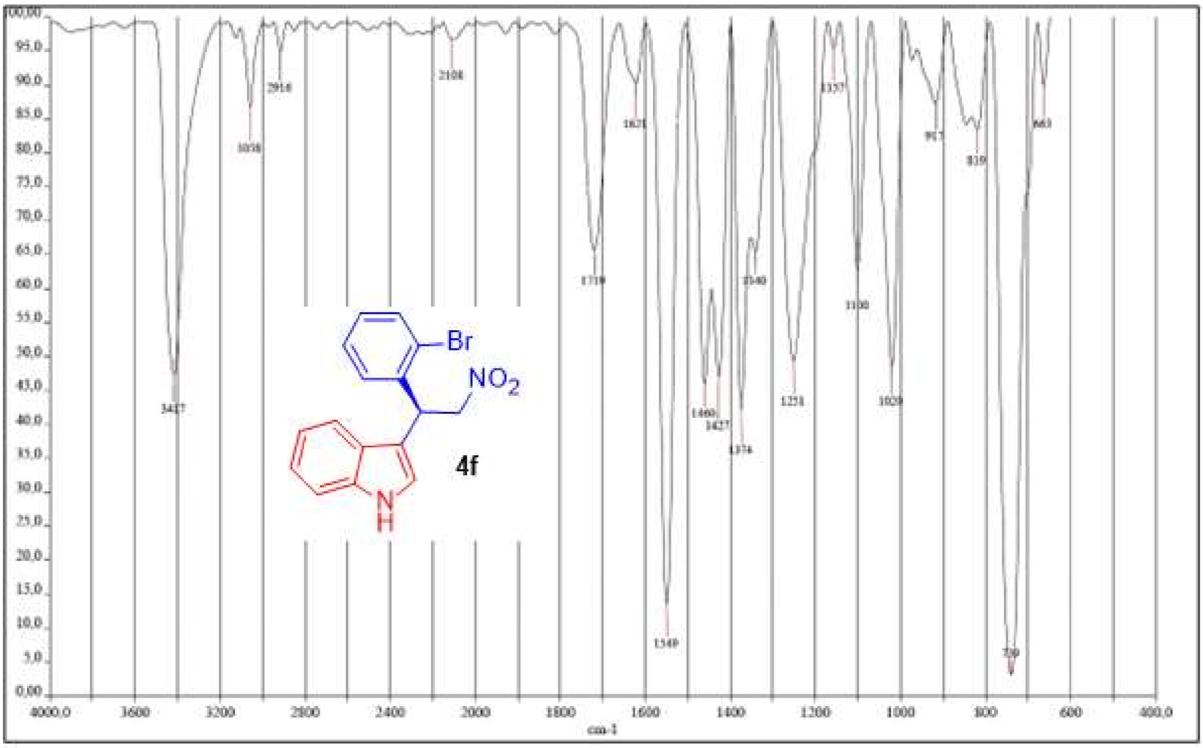

Figure S27: FTIR spectrum of compound **4f**

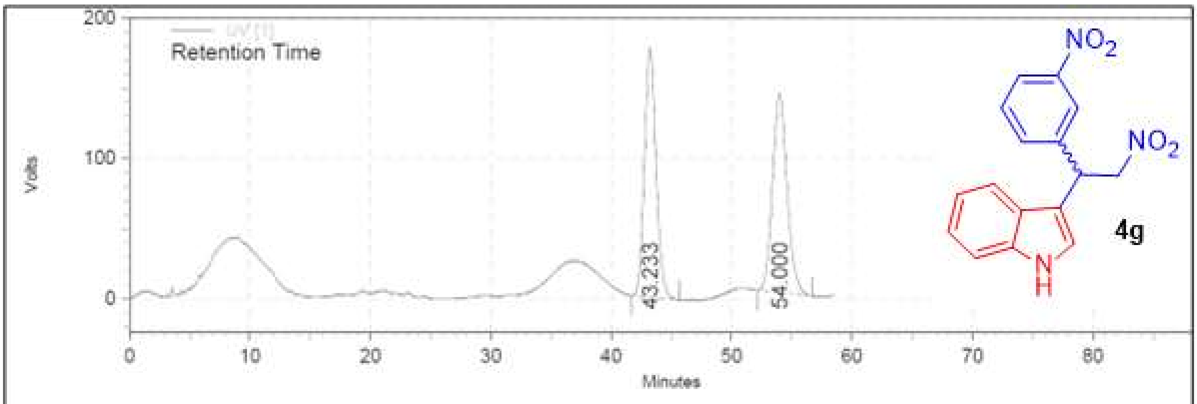

UV [1] Results

Retention Time	Area	Area %	Height	Height %
43.233	11901420	49.77	177139	55.48
54.000	12010255	50.23	142162	44.52
Totals	23911675	100.00	319301	100.00

Figure S28: HPLC chromatogram for racemic mixture of **4g**

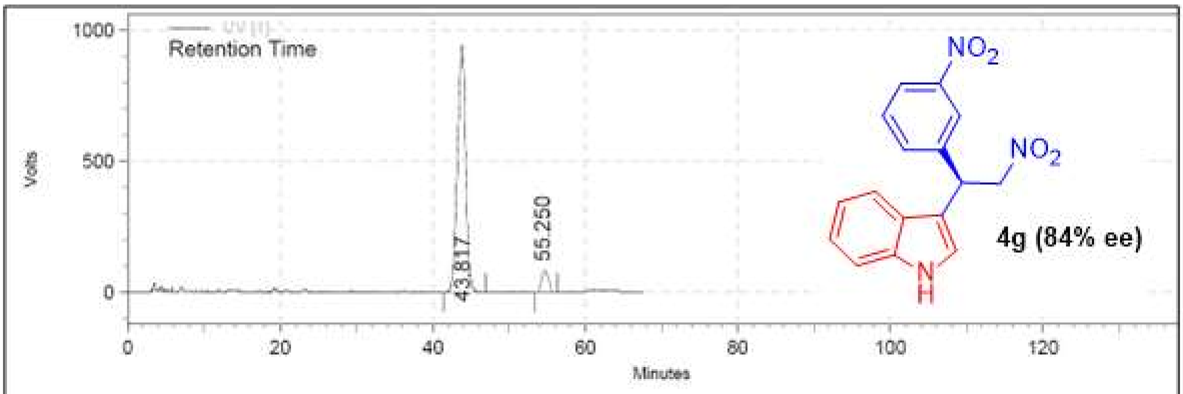

UV [1] Results

Retention Time	Area	Area %	Height	Height %
43.817	70358808	92.07	942870	86.88
55.250	6057946	7.93	142367	13.12
Totals	76416754	100.00	1085237	100.00

Figure S29: HPLC chromatogram for chiral **4g**

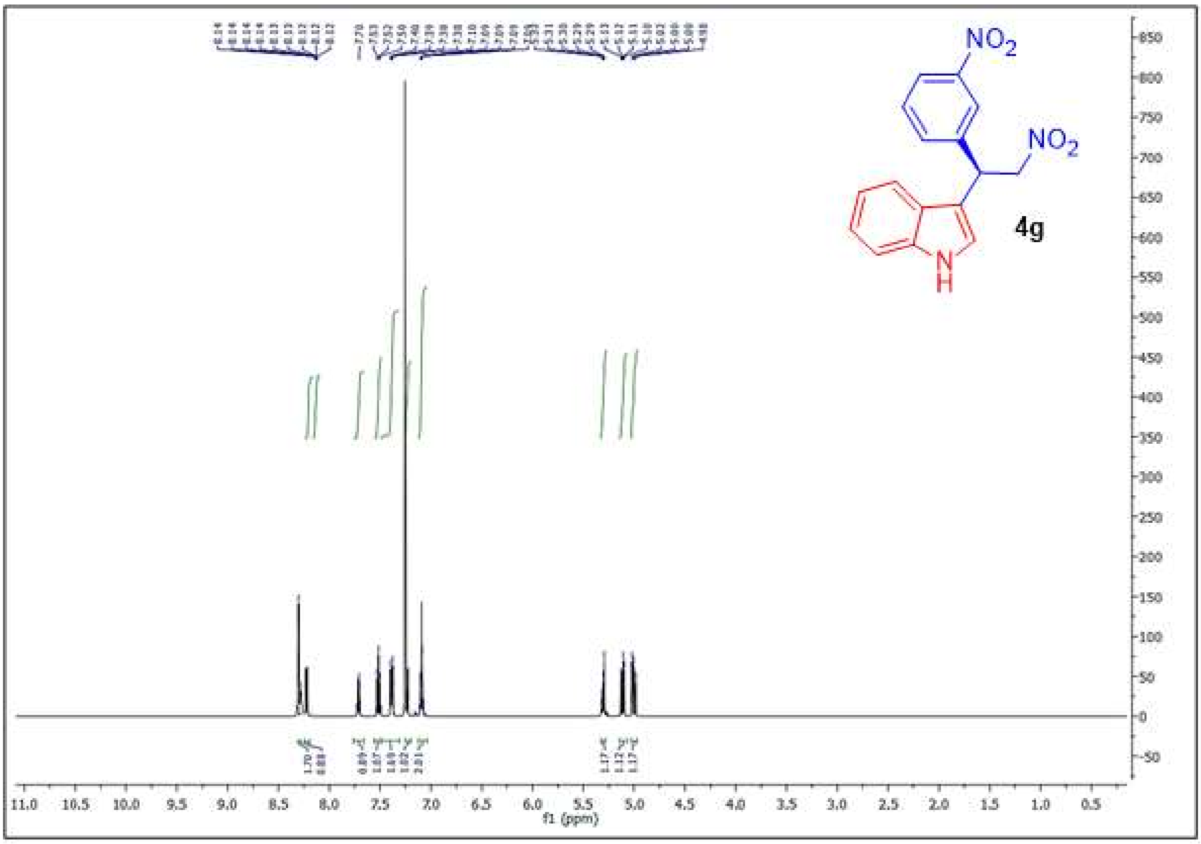

Figure S30: ^{1}HNMR spectrum of compound **4g**

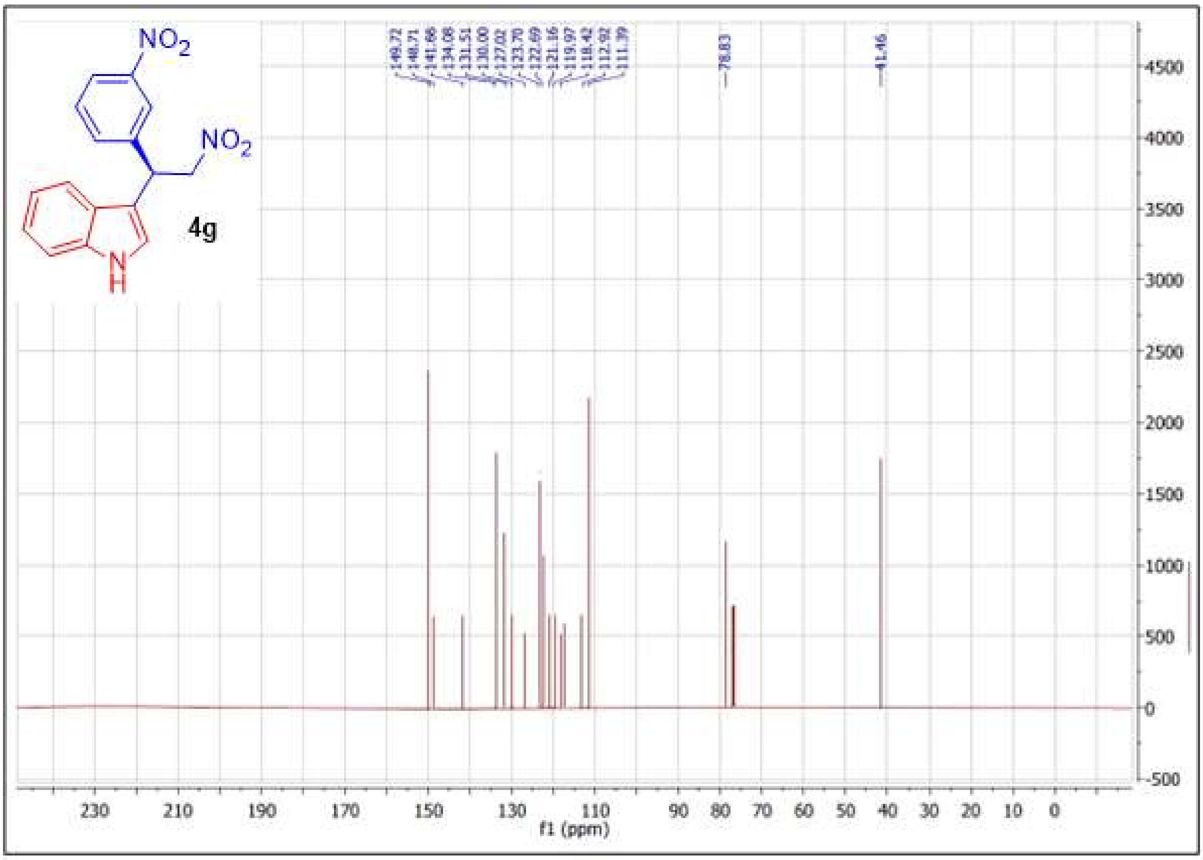

Figure S31: ^{13}CNMR spectrum of compound **4g**

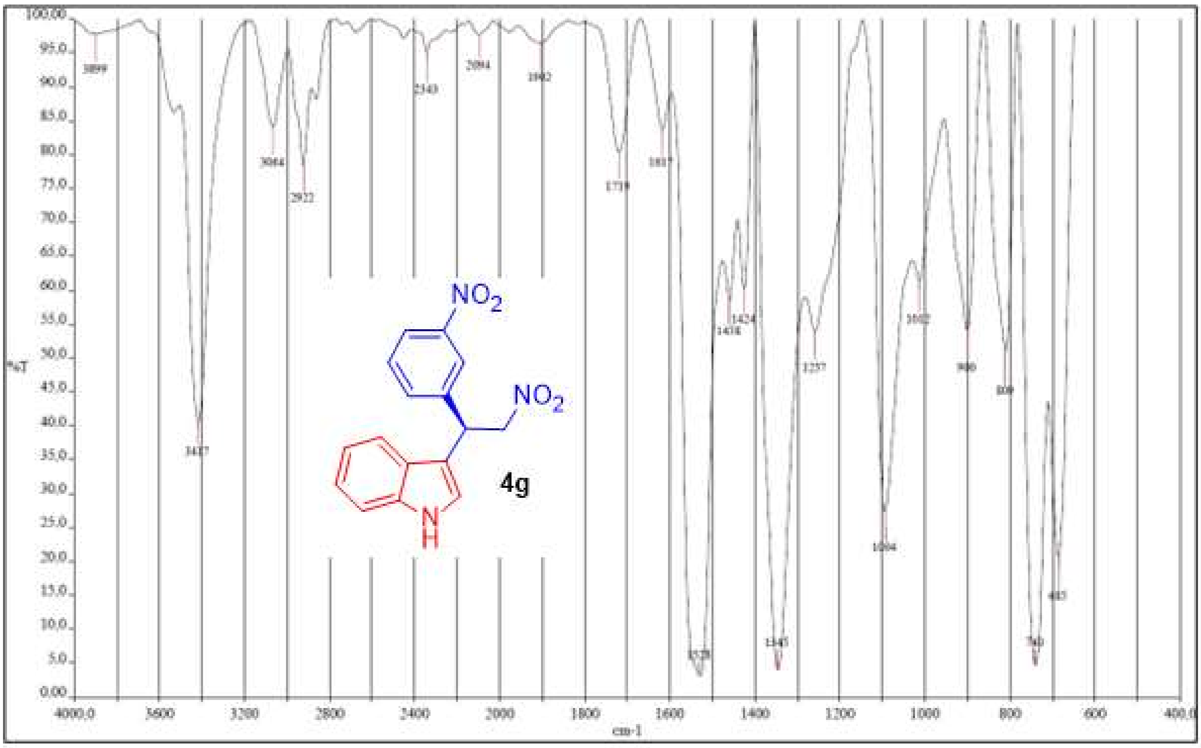

Figure S32: FTIR spectrum of compound **4g**

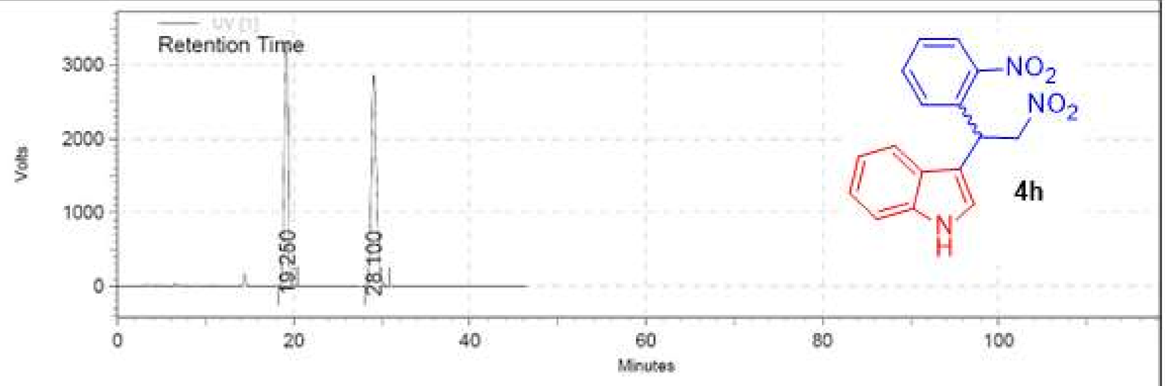

UV [1] Results

Retention Time	Area	Area %	Height	Height %
19.250	23046759	50.41	549986	53.72
28.100	22672160	49.59	473797	46.28
Totals	104507369	100.00	3440958	100.00

Figure S33: HPLC chromatogram for racemic mixture of **4h**

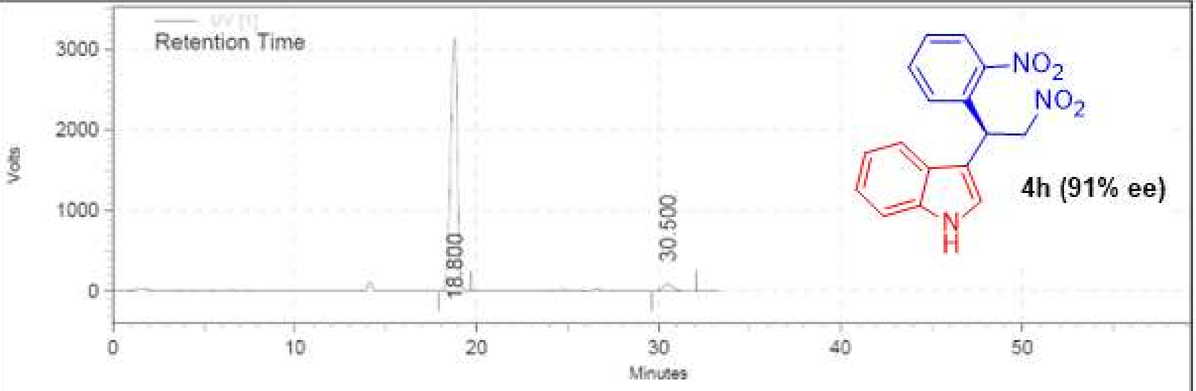

UV [1] Results

Retention Time	Area	Area %	Height	Height %
18.800	81437400	95.76	3133568	97.25
30.500	3607927	4.24	88693	2.75
Totals	85045327	100.00	3222261	100.00

Figure S34: HPLC chromatogram for chiral **4h**

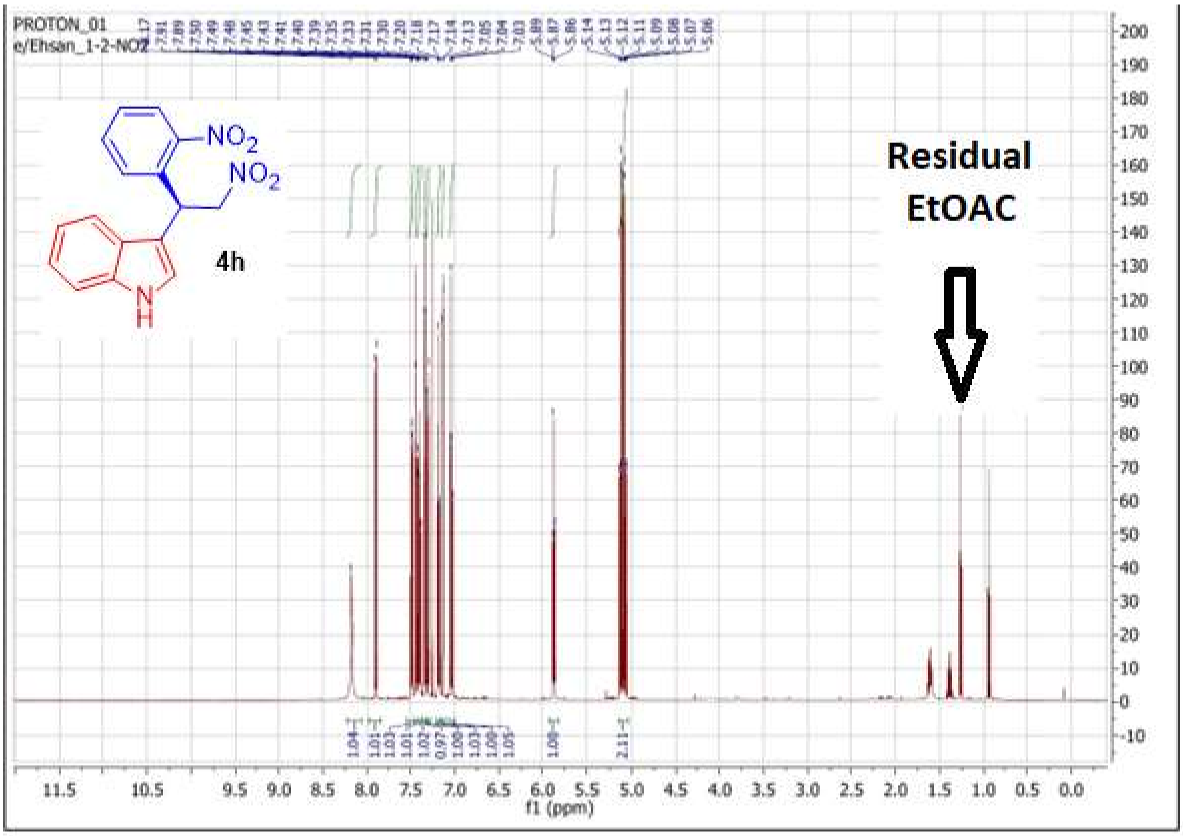

Figure S35: ^{1}HNMR spectrum of compound **4h** (purity of product is 97% according to ^{1}HNMR)

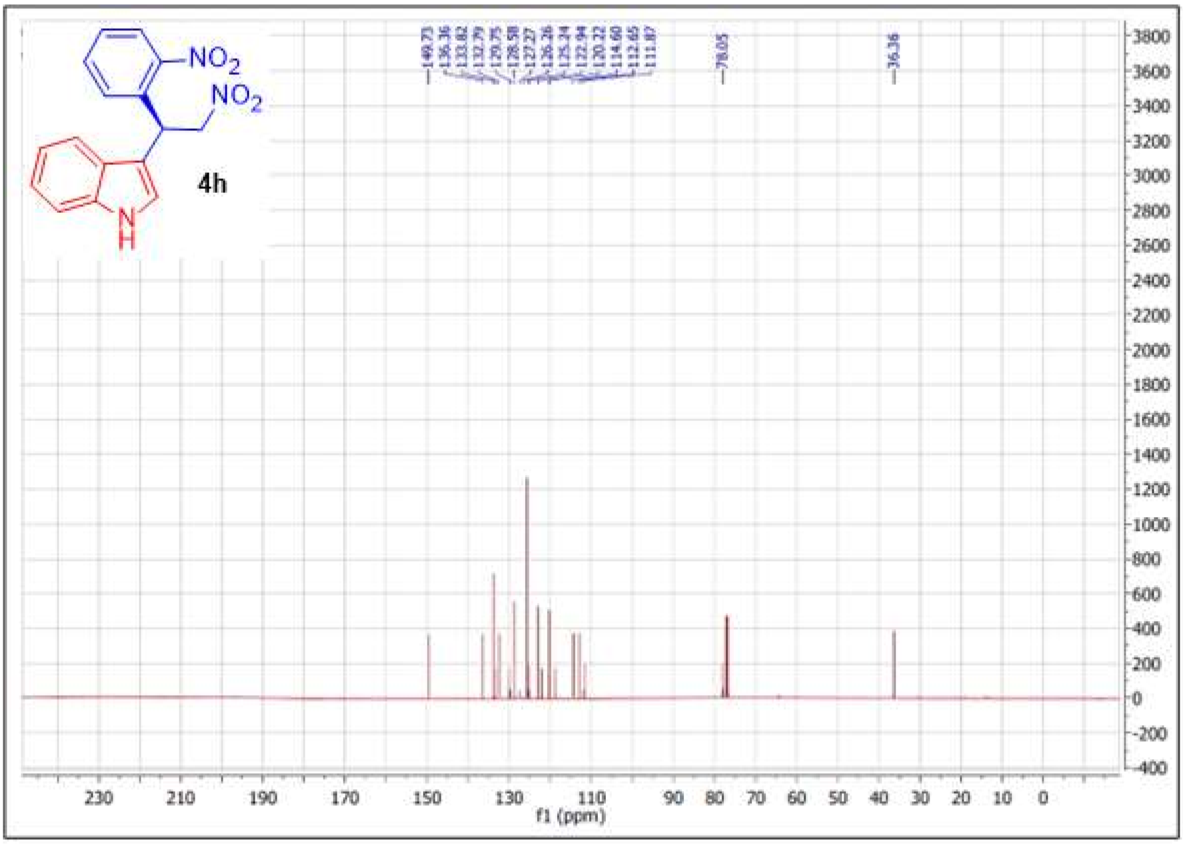

Figure S36: ^{13}CNMR spectrum of compound **4h**

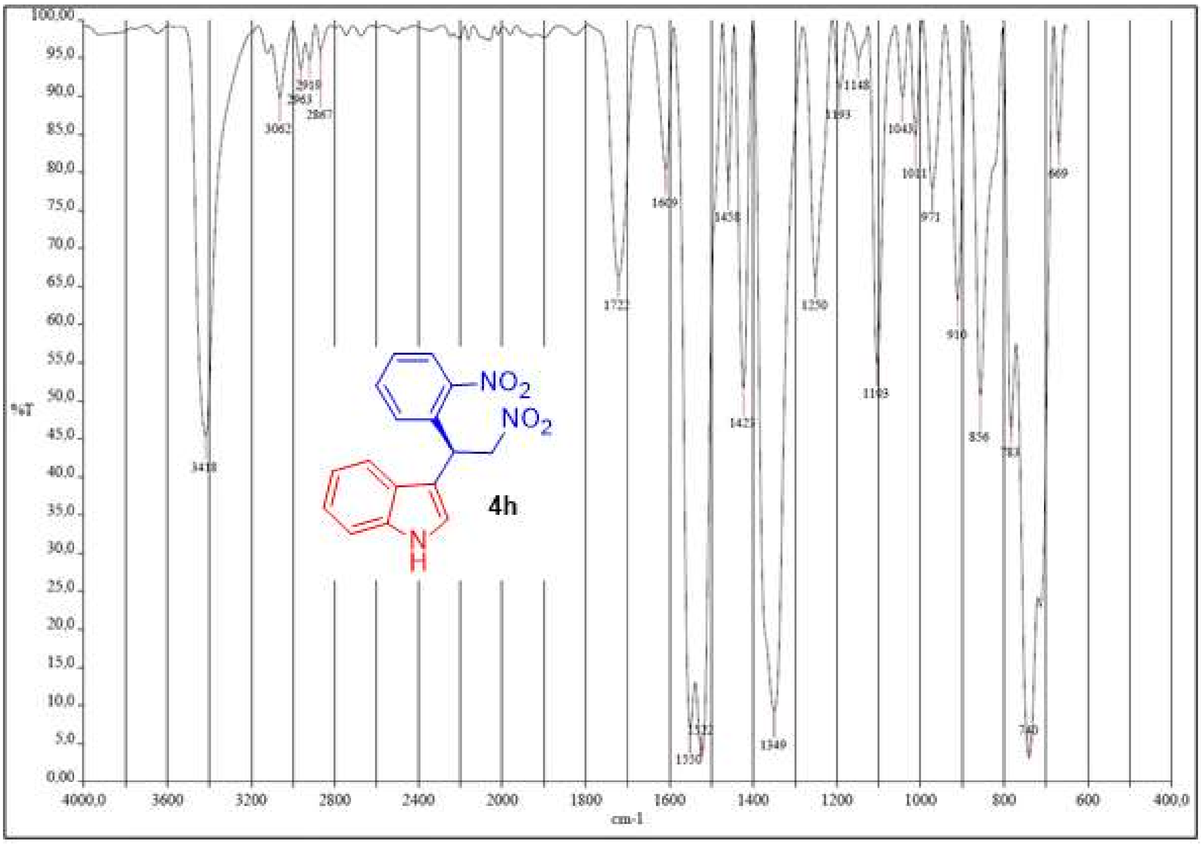

Figure S37: FTIR spectrum of compound **4h**

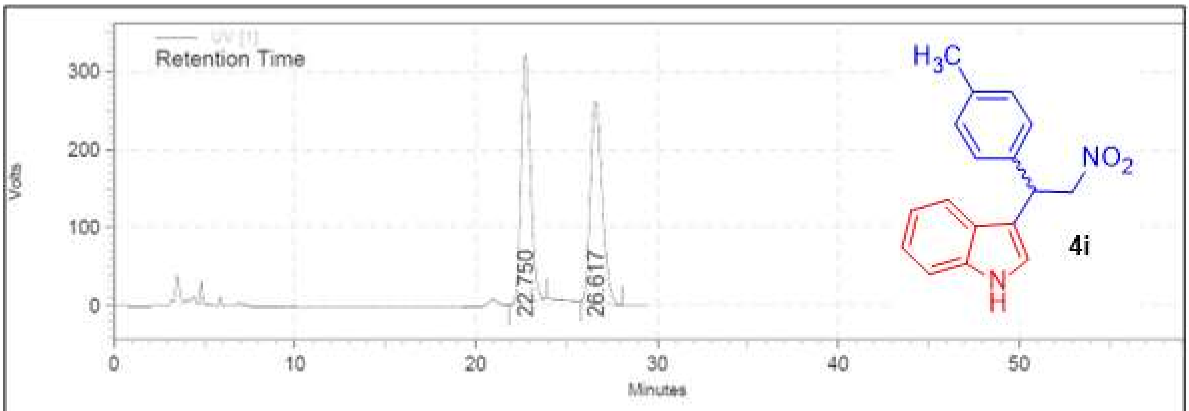

UV [1] Results

Retention Time	Area	Area %	Height	Height %
22.750	11253202	50.79	316768	55.10
26.617	10903332	49.21	258118	44.90
Totals	22156534	100.00	574886	100.00

Figure S38: HPLC chromatogram for racemic mixture of **4i**

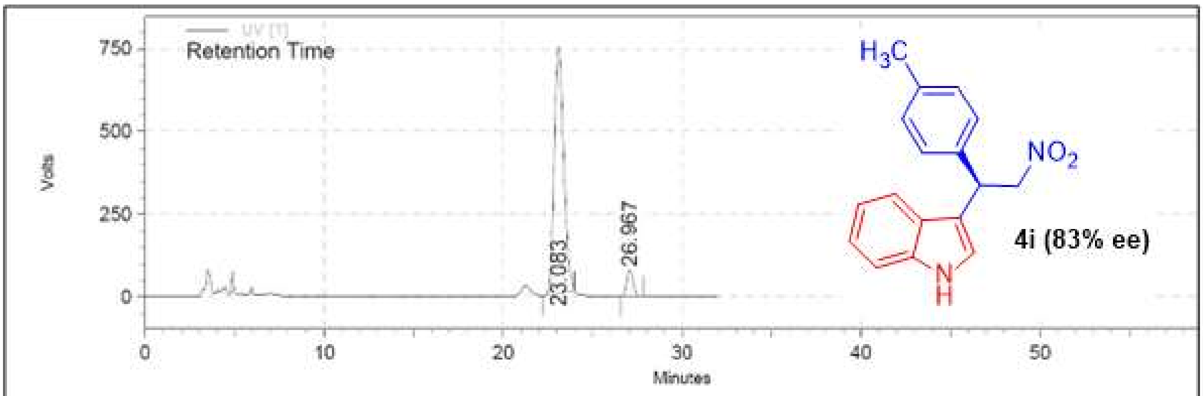

UV [1] Results

Retention Time	Area	Area %	Height	Height %
23.083	27222419	91.59	742935	88.00
26.967	2501104	8.41	101266	12.00
Totals	29723523	100.00	844201	100.00

Figure S39: HPLC chromatogram for chiral **4i**

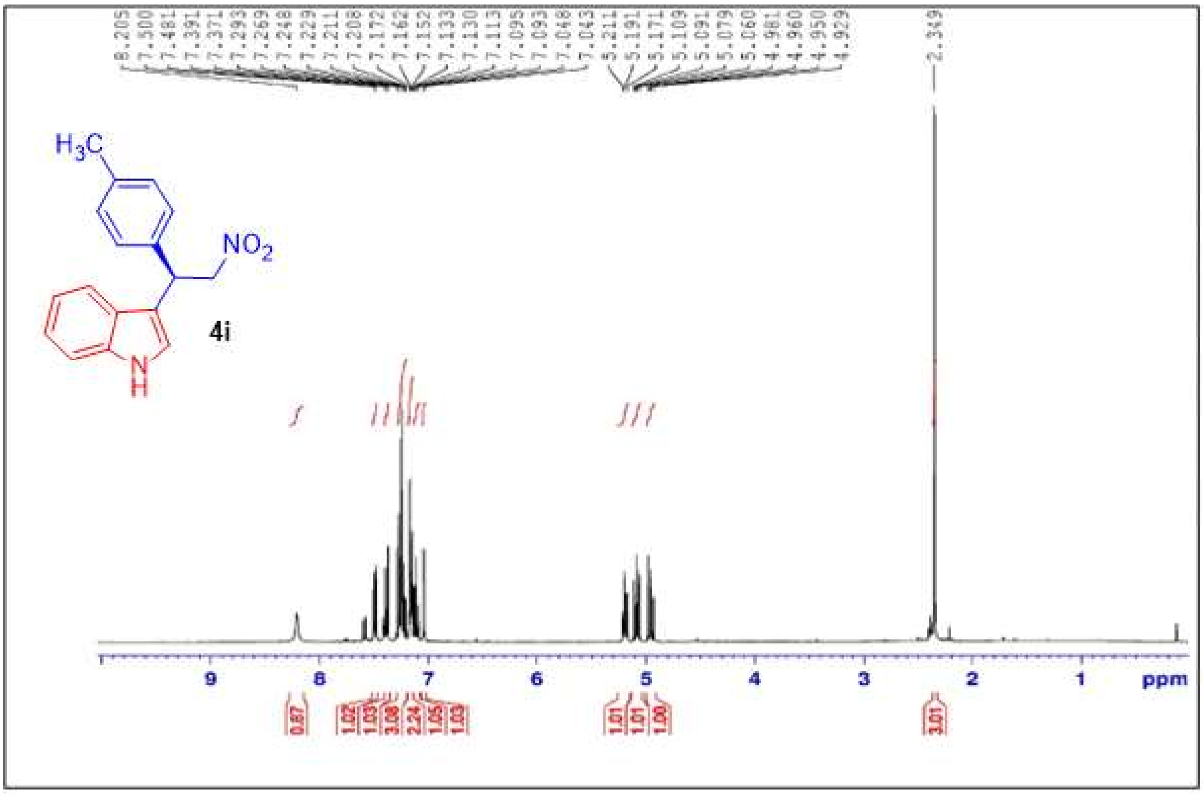

Figure S40: ^{1}HNMR spectrum of compound **4i** (purity of product is 98% according to ^{1}HNMR)

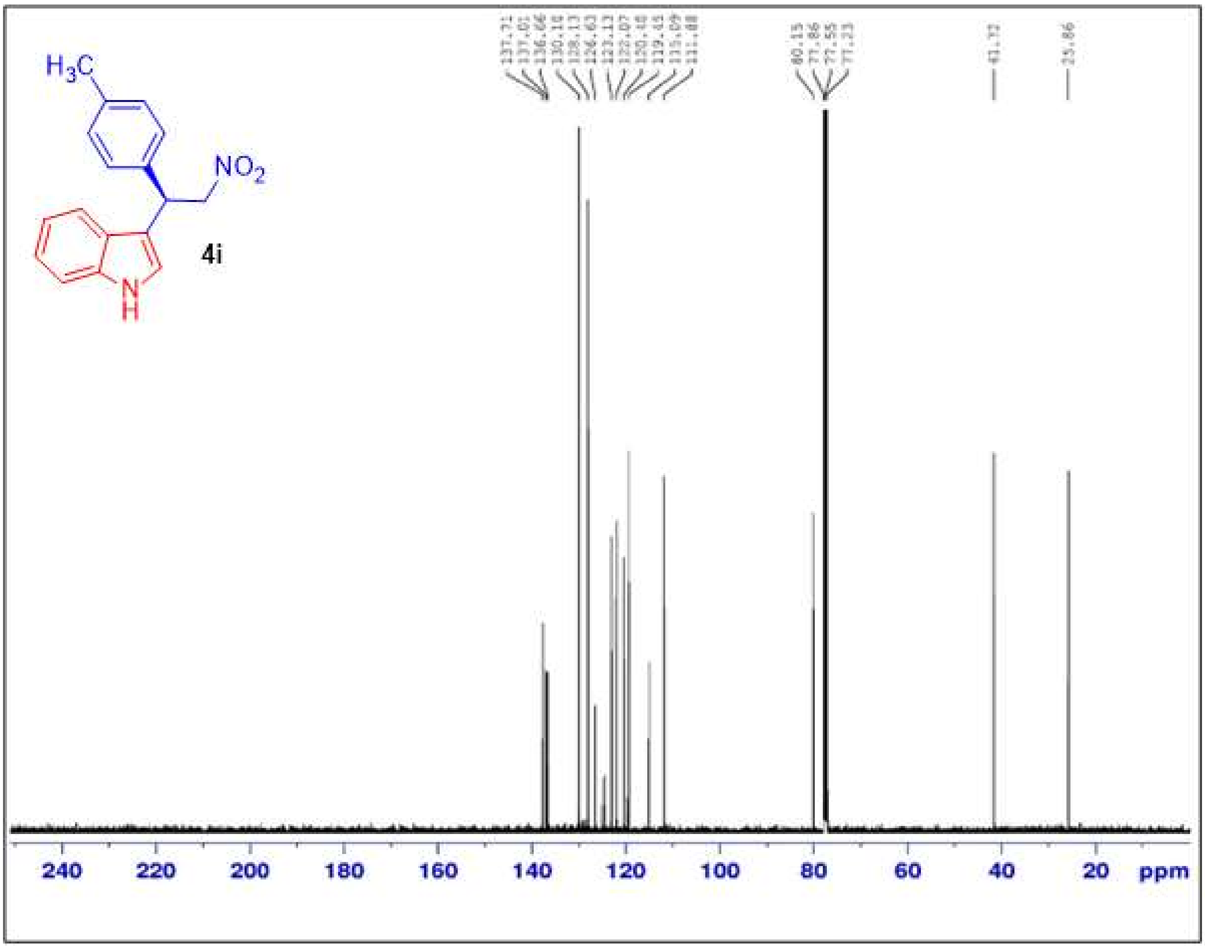

Figure S41: ^{13}CNMR spectrum of compound **4i**

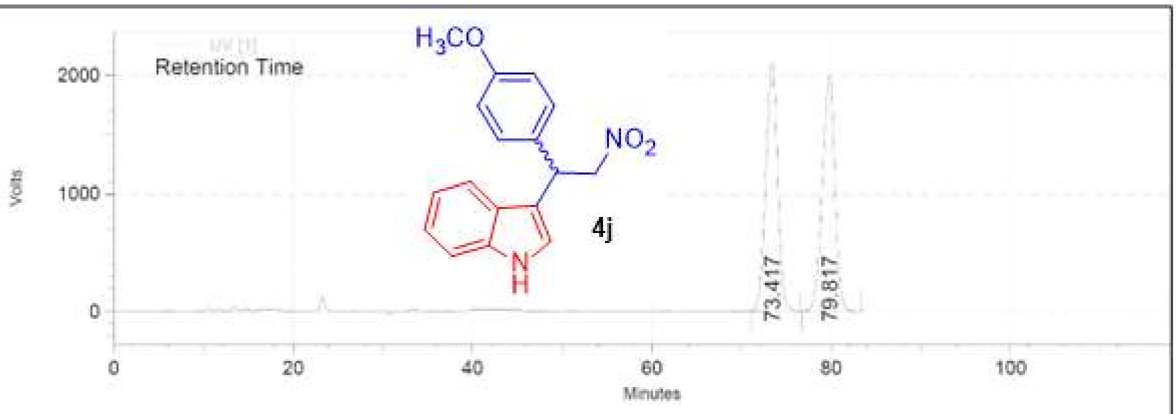

UV [1] Results

Retention Time	Area	Area %	Height	Height %
73.417	195092108	49.88	2101999	51.35
79.817	196038328	50.12	1991749	48.65
Totals	391130436	100.00	4093748	100.00

Figure S42: HPLC chromatogram for racemic mixture of **4j**

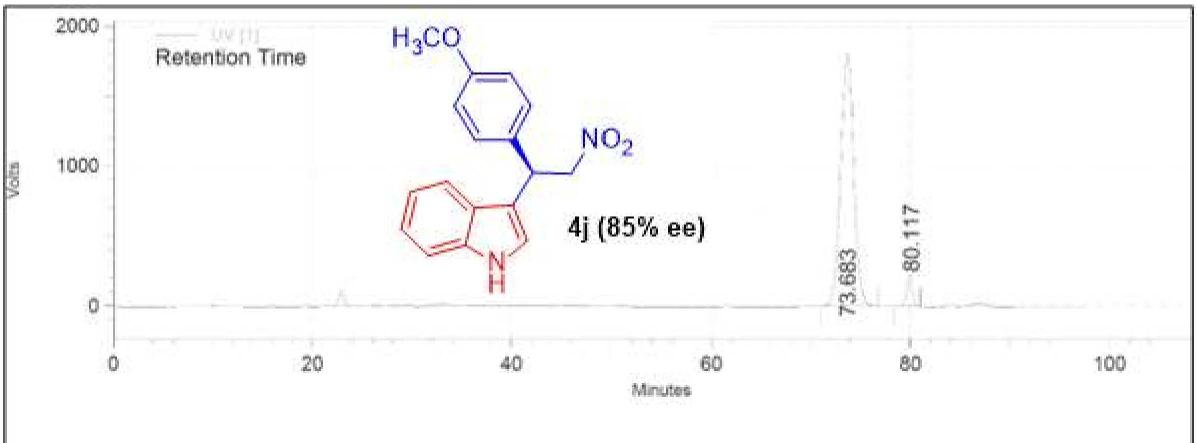

UV [1] Results

Retention Time	Area	Area %	Height	Height %
73.683	165816532	92.50	1808142	88.74
80.117	13447217	7.50	229487	11.26
Totals	179263749	100.00	2037629	100.00

Figure S43: HPLC chromatogram for chiral **4j**

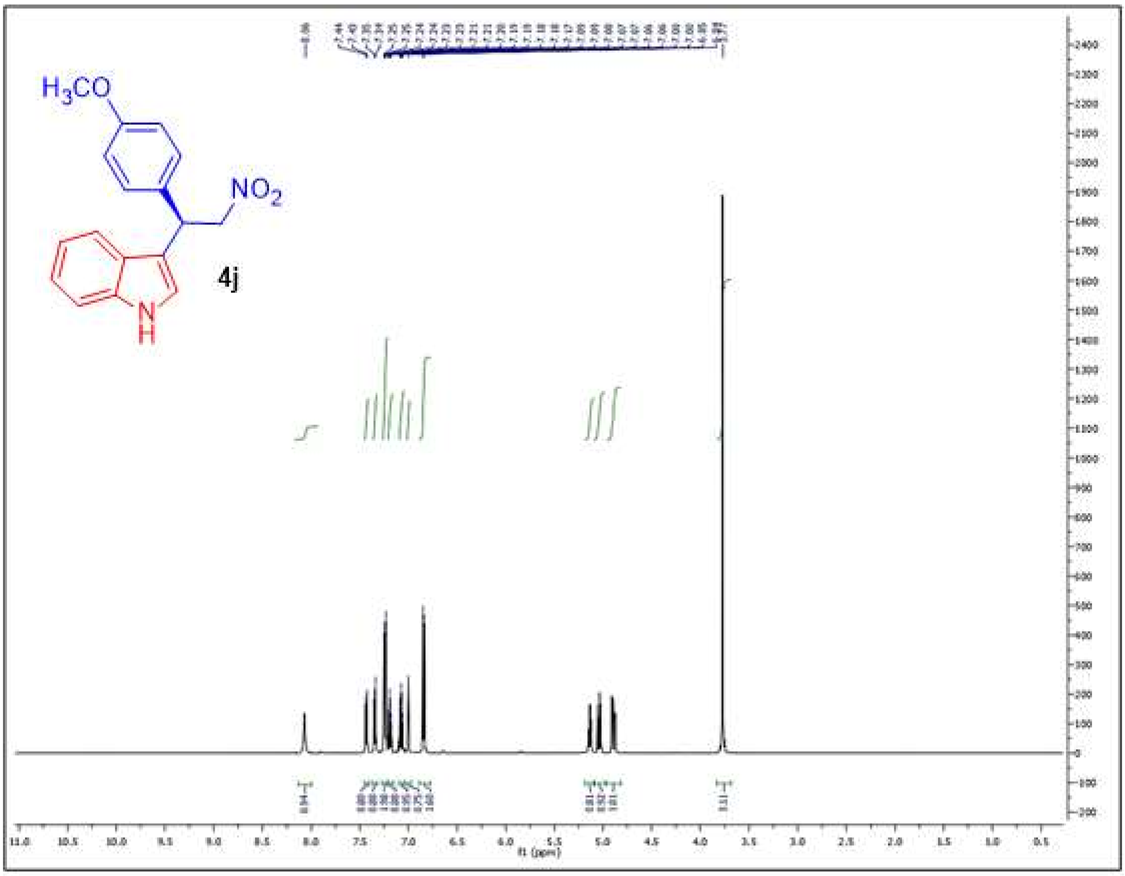

Figure S44: ^{1}HNMR spectrum of compound **4j**

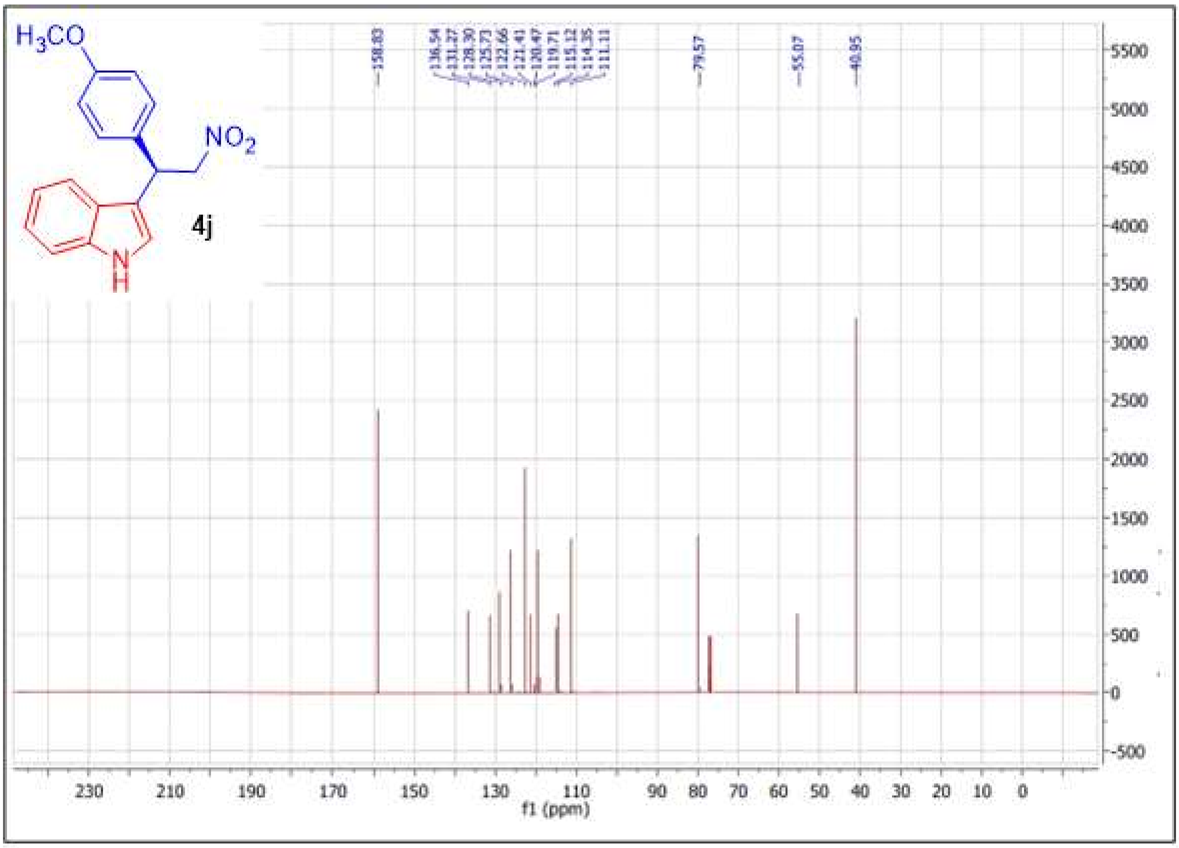

Figure S45: ^{13}CNMR spectrum of compound **4j**

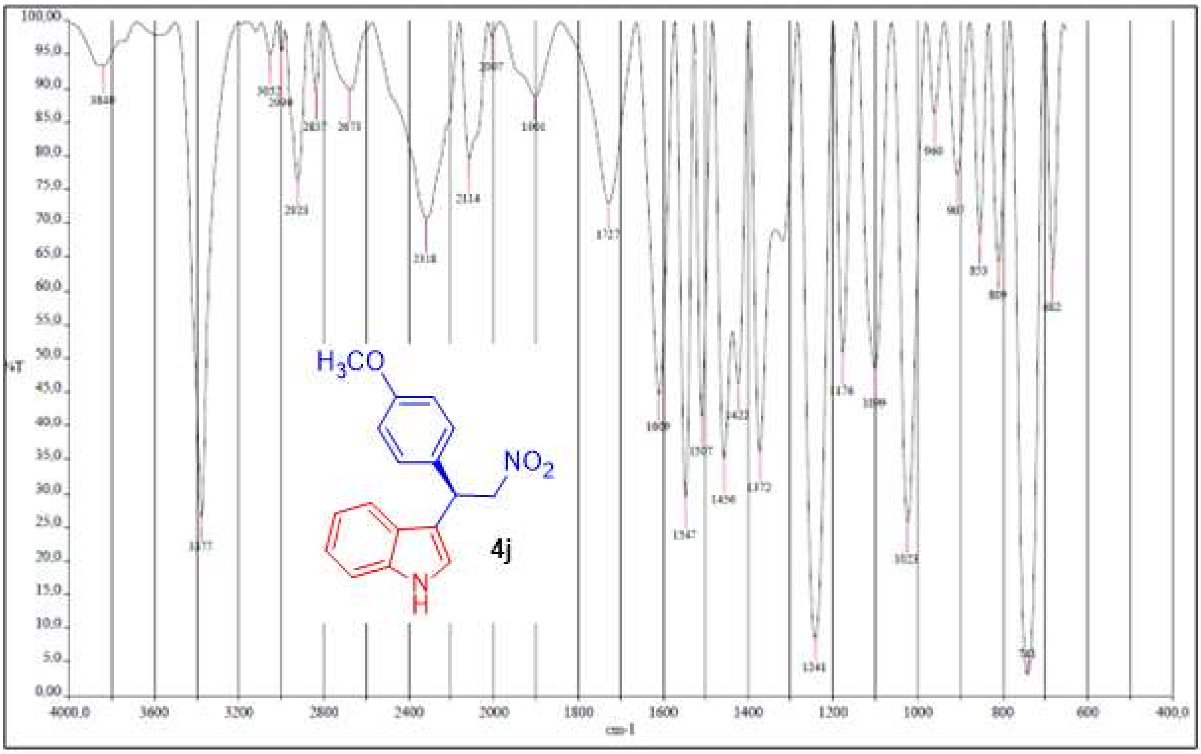

Figure S46: FTIR spectrum of compound **4j**

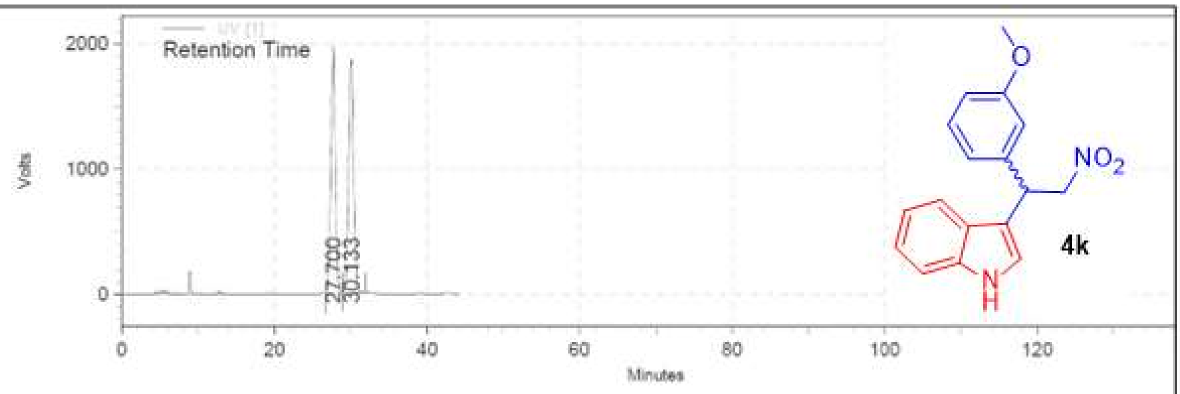

UV [1] Results

Retention Time	Area	Area %	Height	Height %
27.700	84853578	49.78	1976510	51.40
30.133	85615213	50.22	1869101	48.60
Totals	170468791	100.00	3845611	100.00

Figure S47: HPLC chromatogram for racemic mixture of **4k**

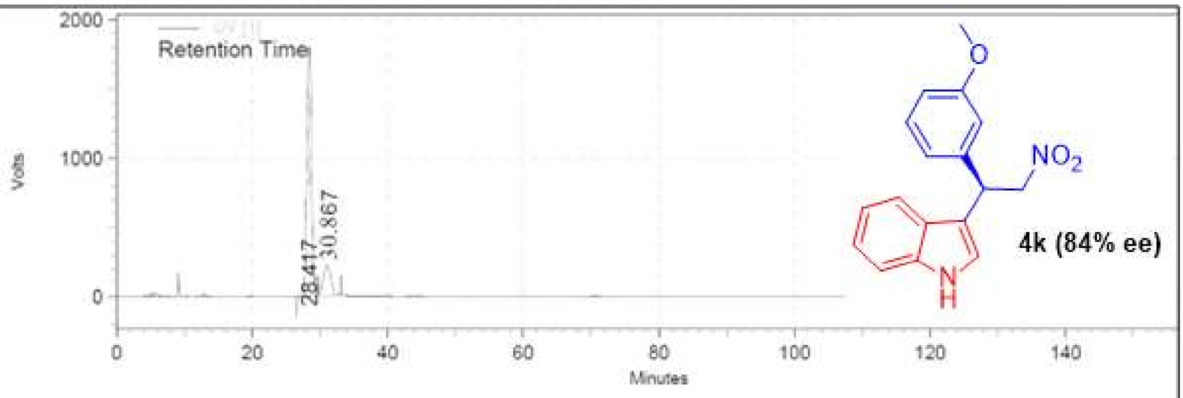

UV [1] Results

Retention Time	Area	Area %	Height	Height %
28.417	79101523	92.06	1808391	87.02
30.867	6822133	7.94	269630	12.98
Totals	85923656	100.00	2078021	100.00

Figure S48: HPLC chromatogram for chiral **4k**

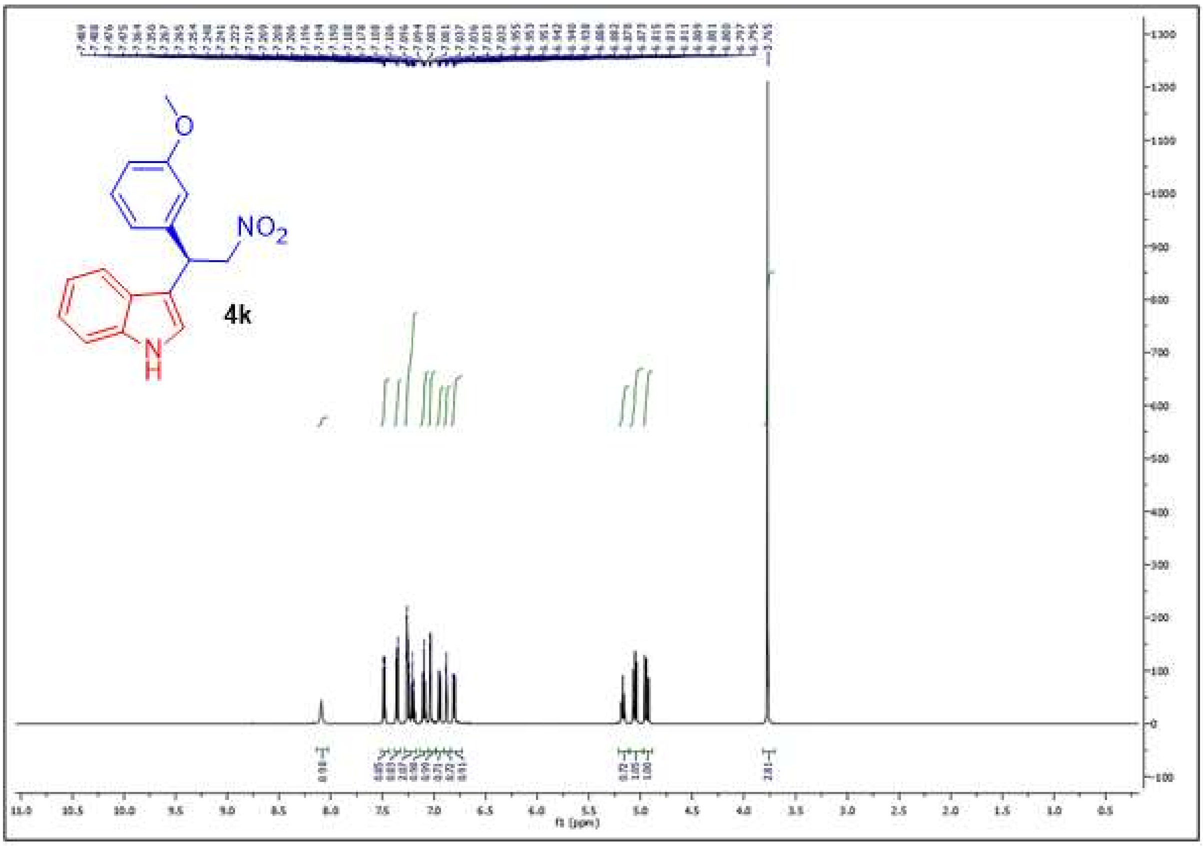

Figure S49: ^{1}HNMR spectrum of compound **4k**

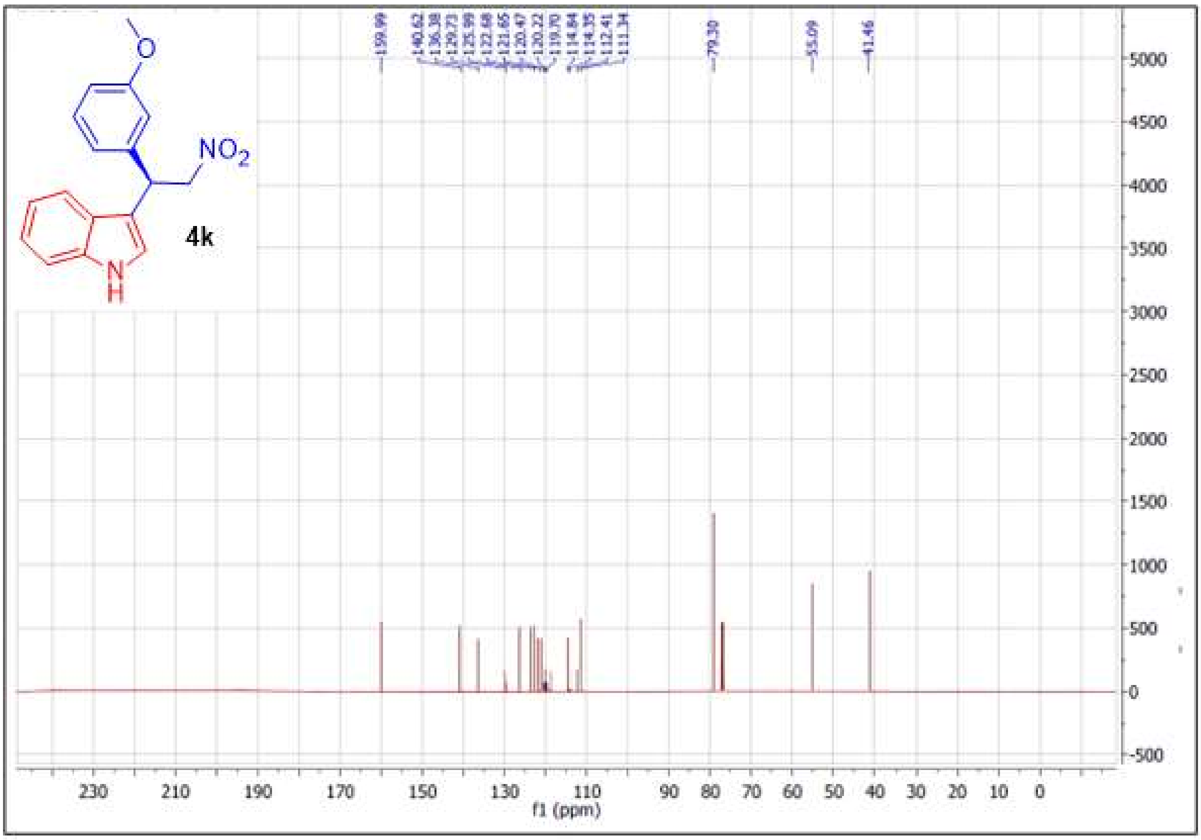

Figure S50: ^{13}CNMR spectrum of compound **4k**

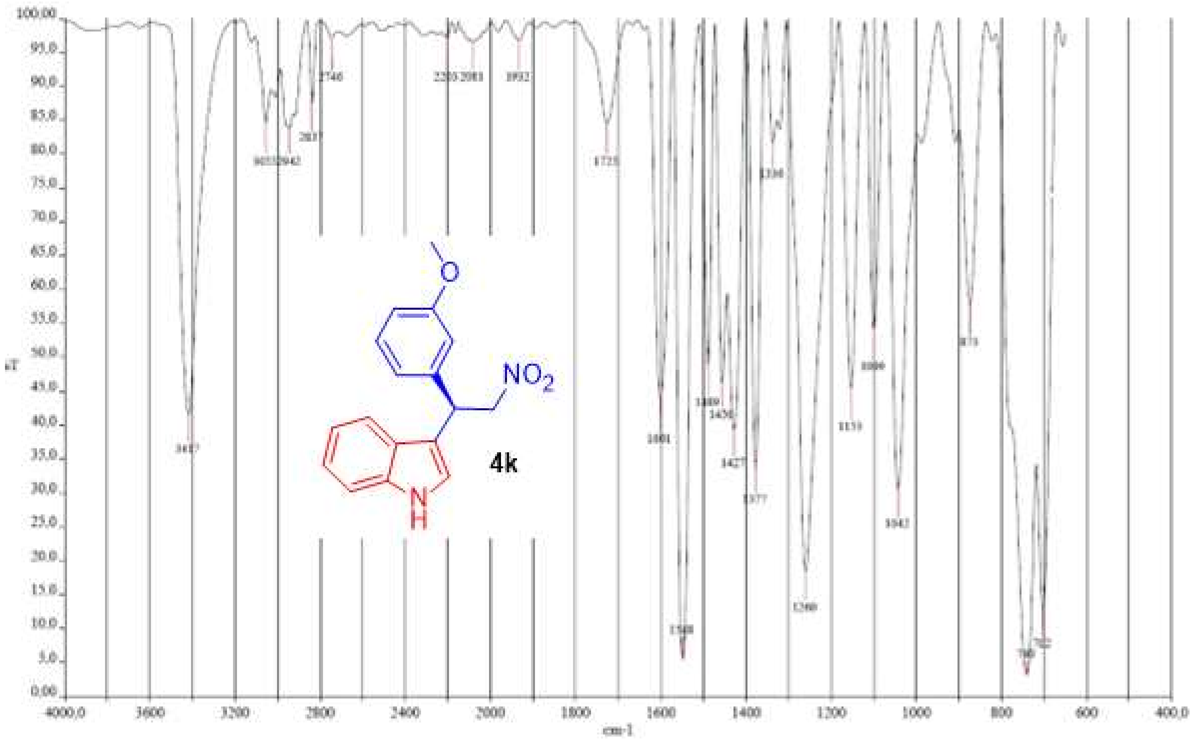

Figure S51: FTIR spectrum of compound **4k**

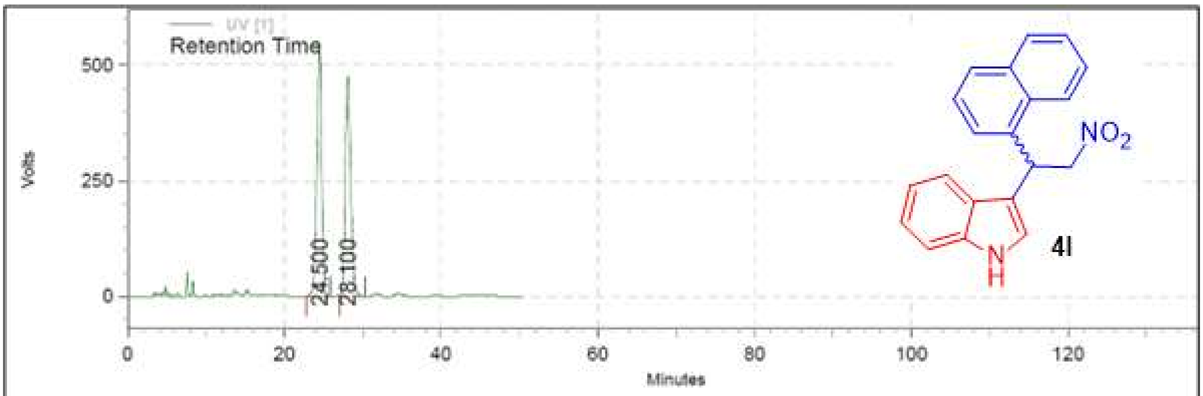

UV [1] Results

Retention Time	Area	Area %	Height	Height %
24.500	23046759	50.41	549986	53.72
28.100	22672160	49.59	473797	46.28
Totals	45718919	100.00	1023783	100.00

Figure S52: HPLC chromatogram for racemic mixture of **4l**

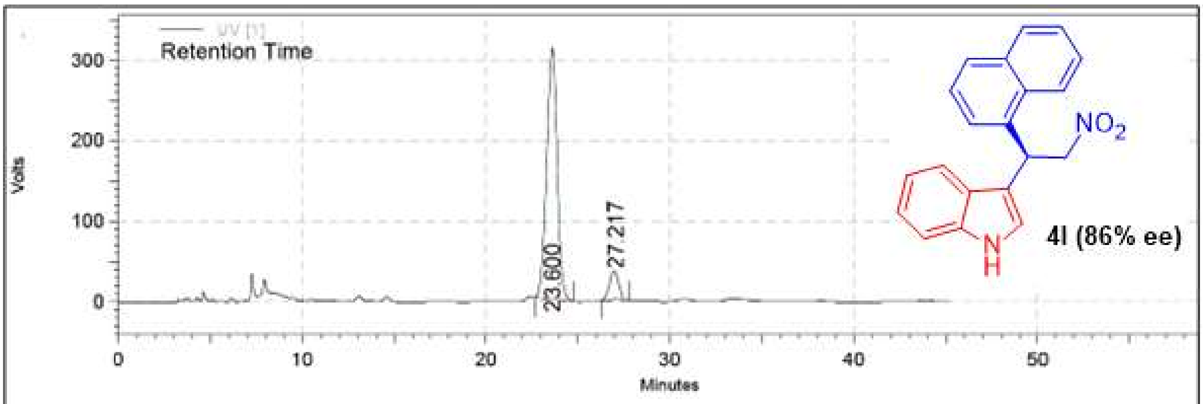

UV [1] Results

Retention Time	Area	Area %	Height	Height %
23.600	12370786	93.06	312781	91.18
27.217	922245	6.94	30267	8.82
Totals	13293031	100.00	343048	100.00

Figure S53: HPLC chromatogram for chiral **4l**

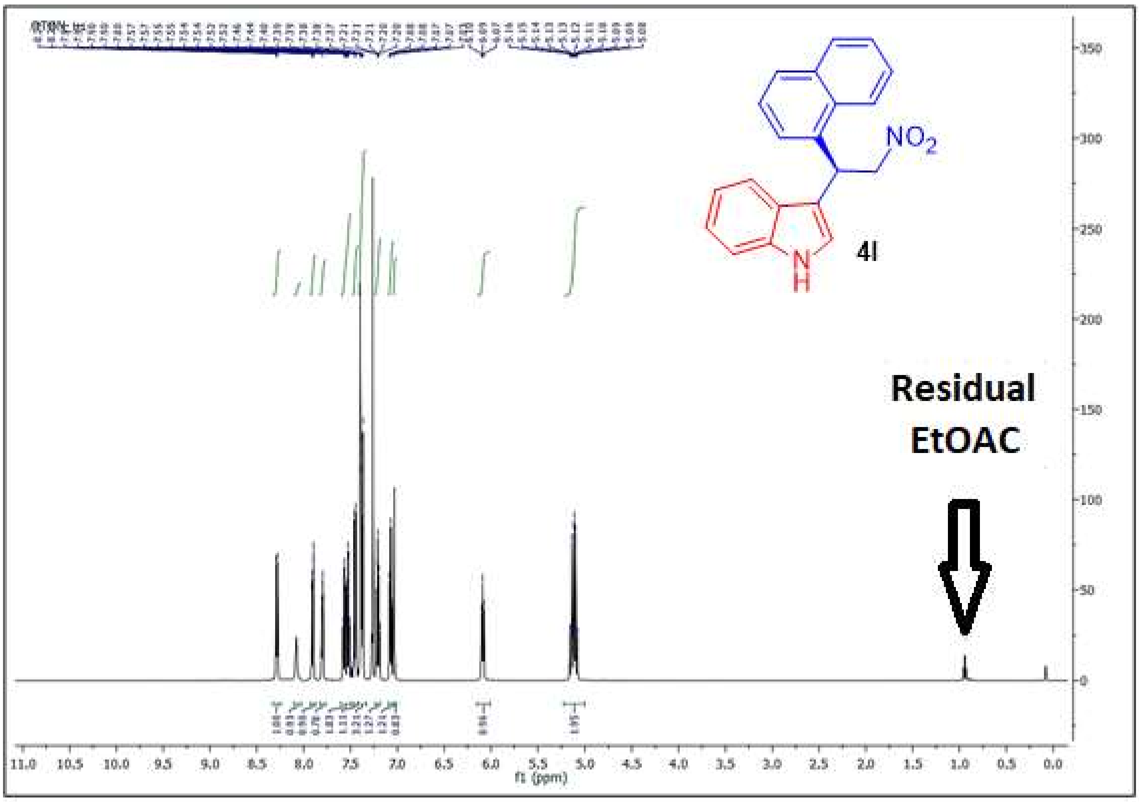

Figure S54: ^{1}HNMR spectrum of compound **4l**

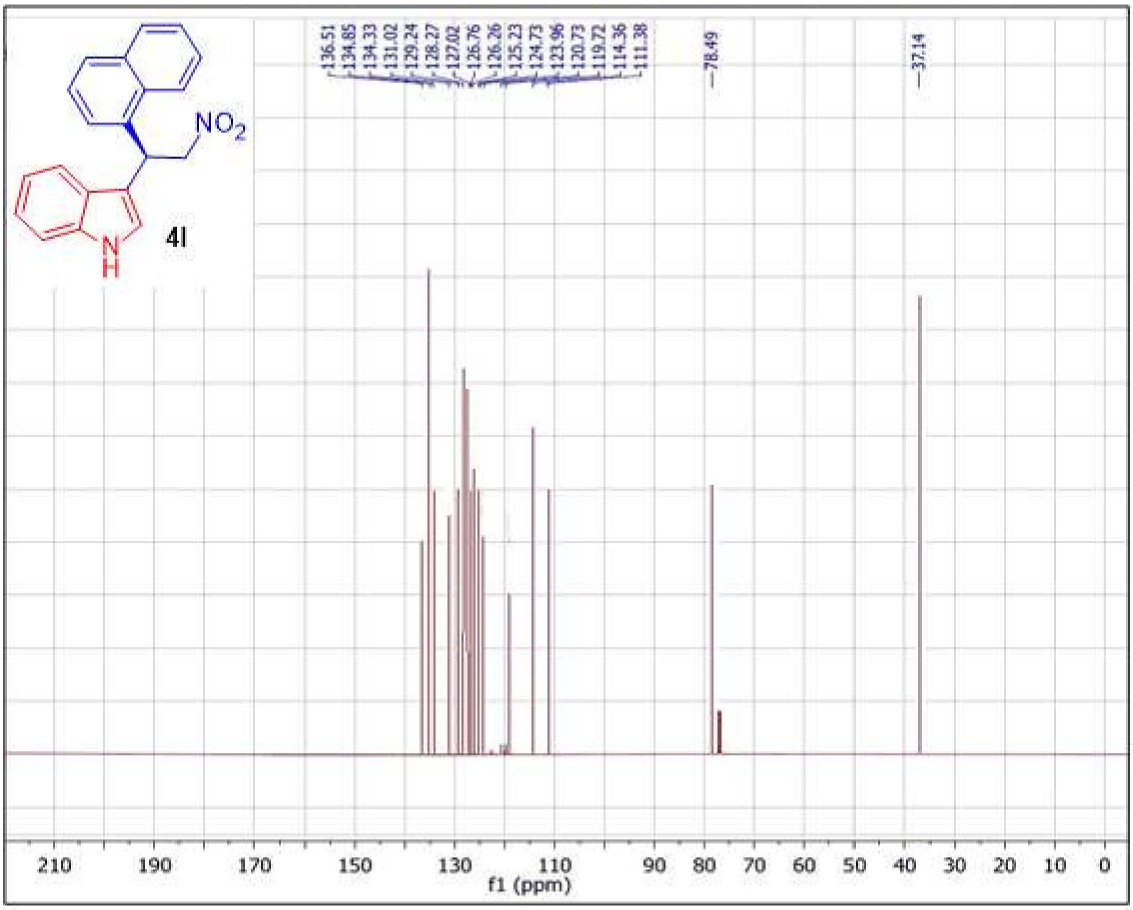

Figure S55: ^{13}CNMR spectrum of compound **4l**

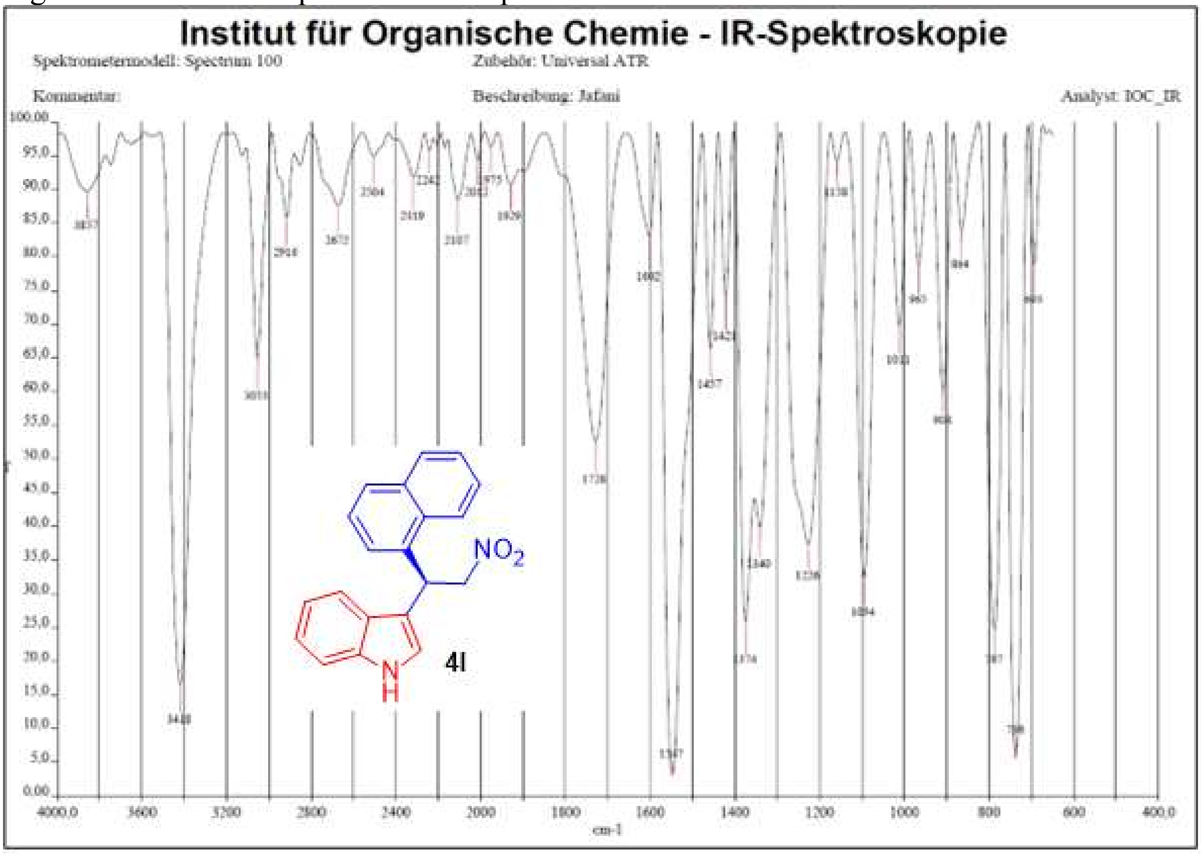

Figure S56: FTIR spectrum of compound **4l**

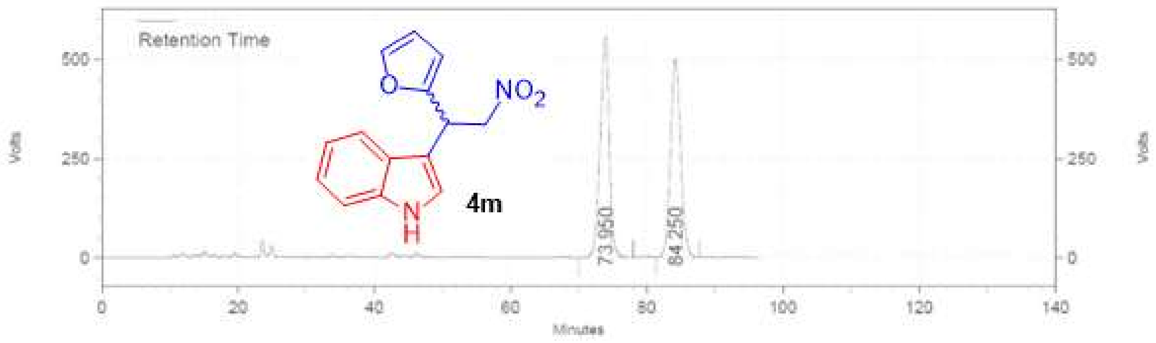

UV [1] Results

Retention Time	Area	Area %	Height	Height %
73.950	51232383	50.22	556359	52.63
84.250	50775877	49.78	500850	47.37
Totals	102008260	100.00	1057209	100.00

Figure S57: HPLC chromatogram for racemic mixture of **4m**

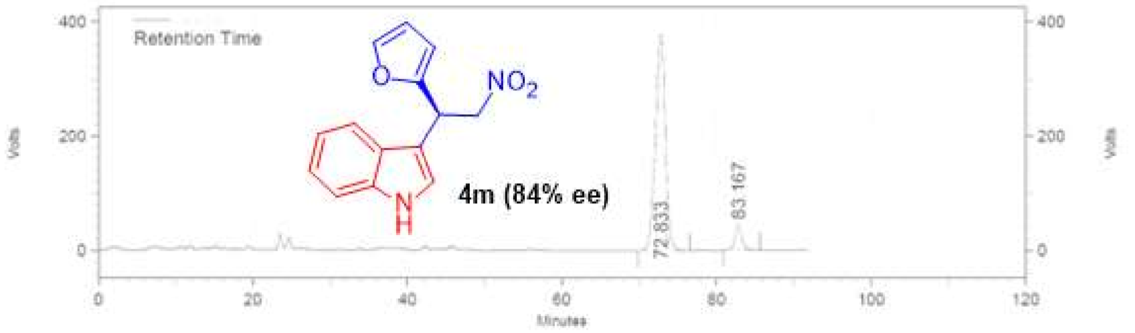

UV [1] Results

Retention Time	Area	Area %	Height	Height %
72.833	33120391	92.15	376918	85.42
83.167	2820958	7.85	64334	14.58
Totals	35941349	100.00	441252	100.00

Figure S58: HPLC chromatogram for chiral **4m**

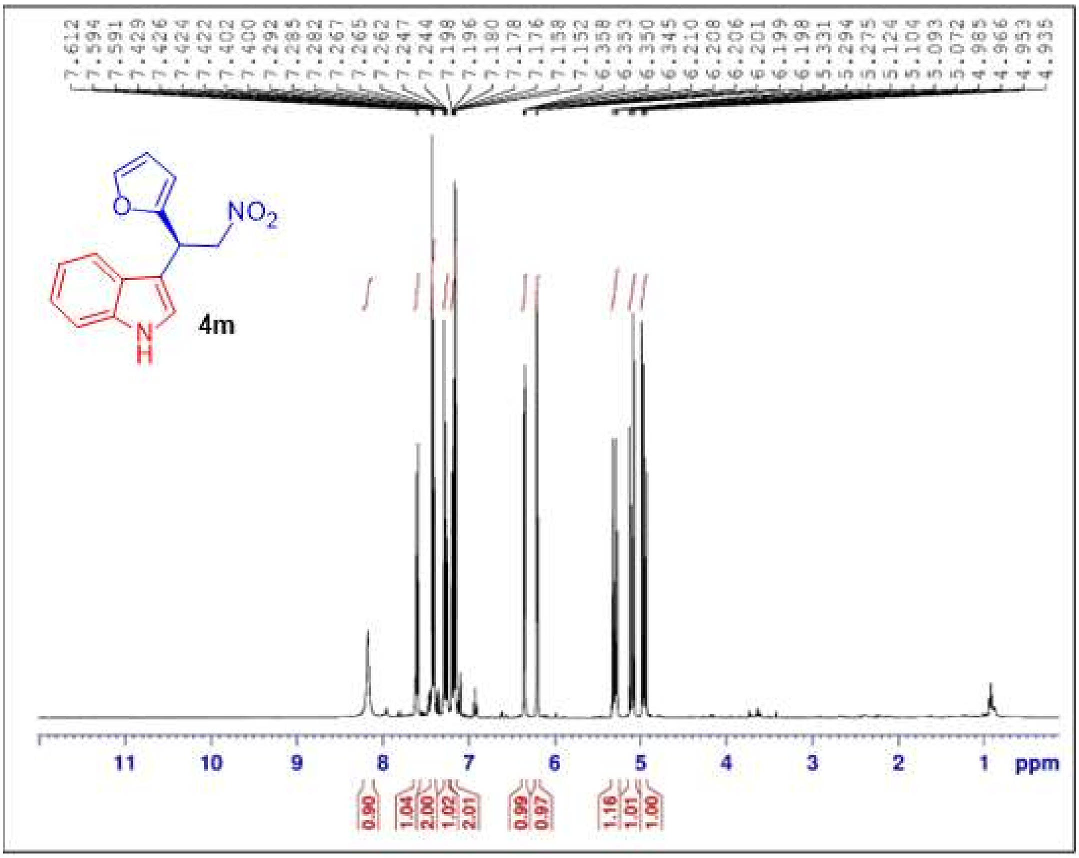

Figure S59: ^{1}HNMR spectrum of compound **4m** (purity of product is 96% according to ^{1}HNMR)

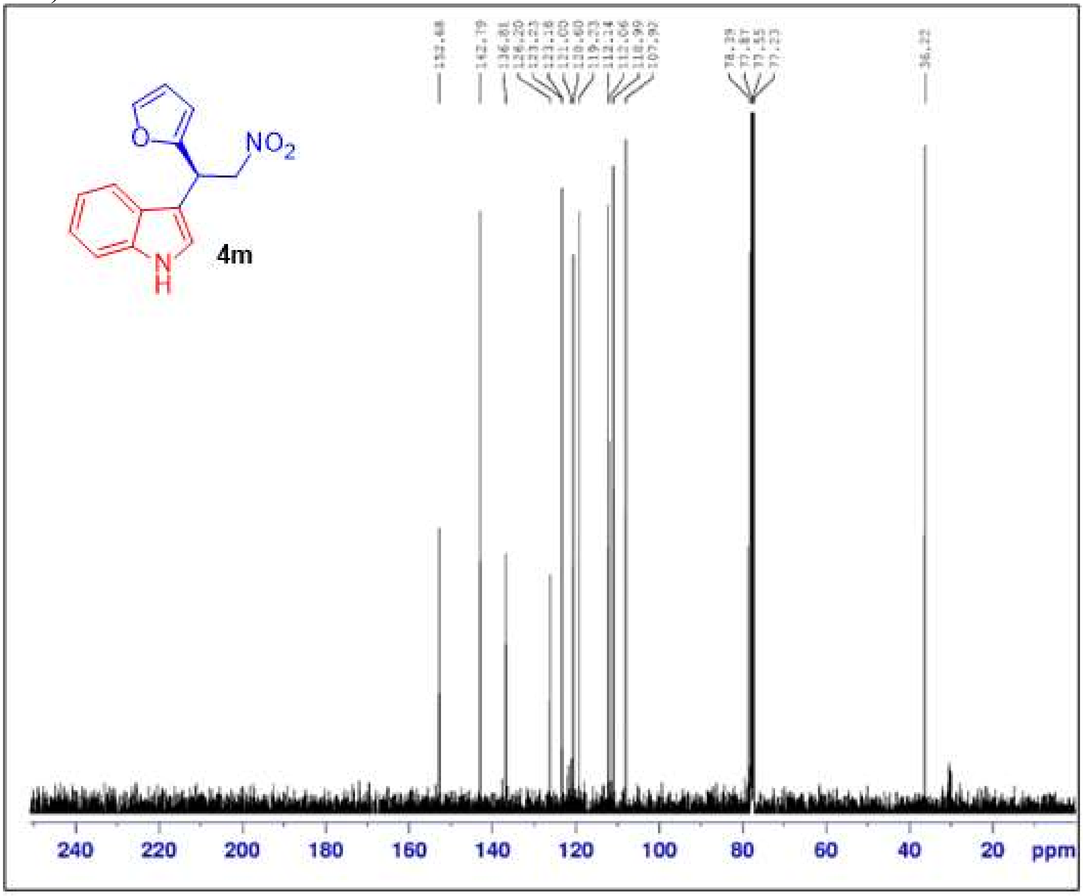

Figure S60: ^{13}CNMR spectrum of compound **4m**

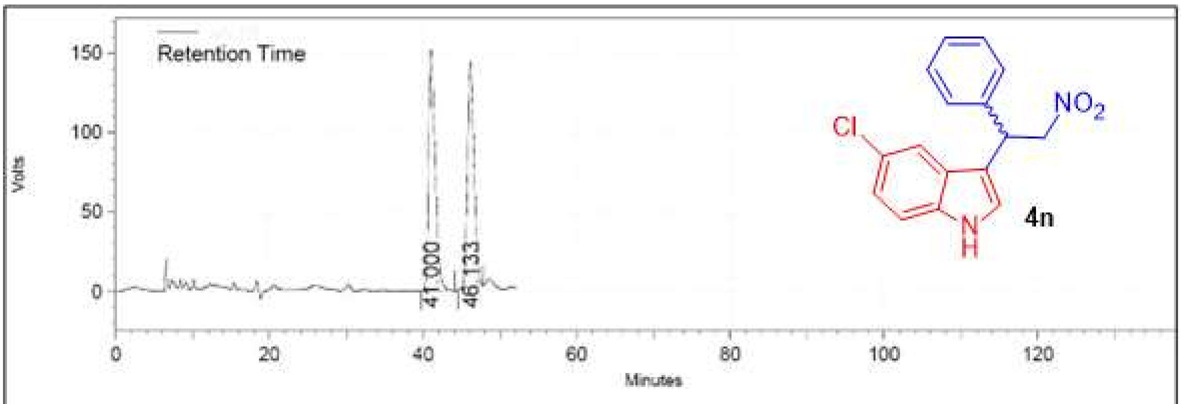

UV [1] Results

Retention Time	Area	Area %	Height	Height %
41.000	8966523	50.89	151106	51.59
46.133	8653051	49.11	141774	48.41
Totals	17619574	100.00	292880	100.00

Figure S61: HPLC chromatogram for racemic mixture of **4n**

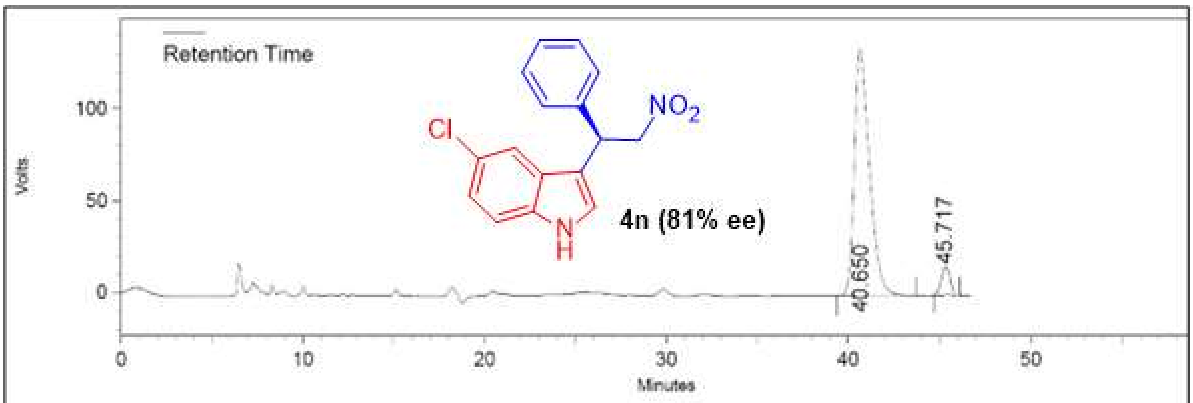

UV [1] Results

Retention Time	Area	Area %	Height	Height %
40.650	7779173	90.56	133573	83.12
45.717	810524	9.44	17117	16.88
Totals	8589697	100.00	160690	100.00

Figure S62: HPLC chromatogram for chiral **4n**

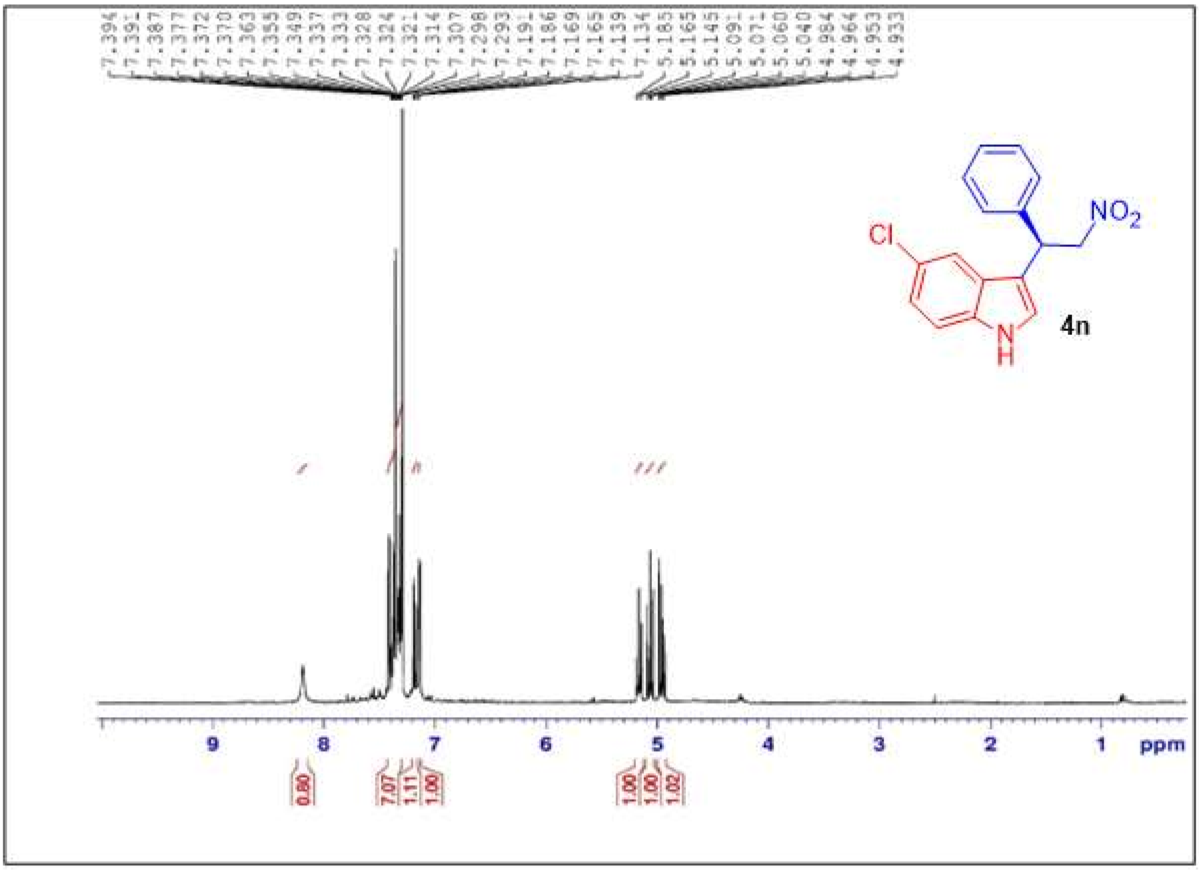

Figure S63: ^{1}HNMR spectrum of compound **4n**

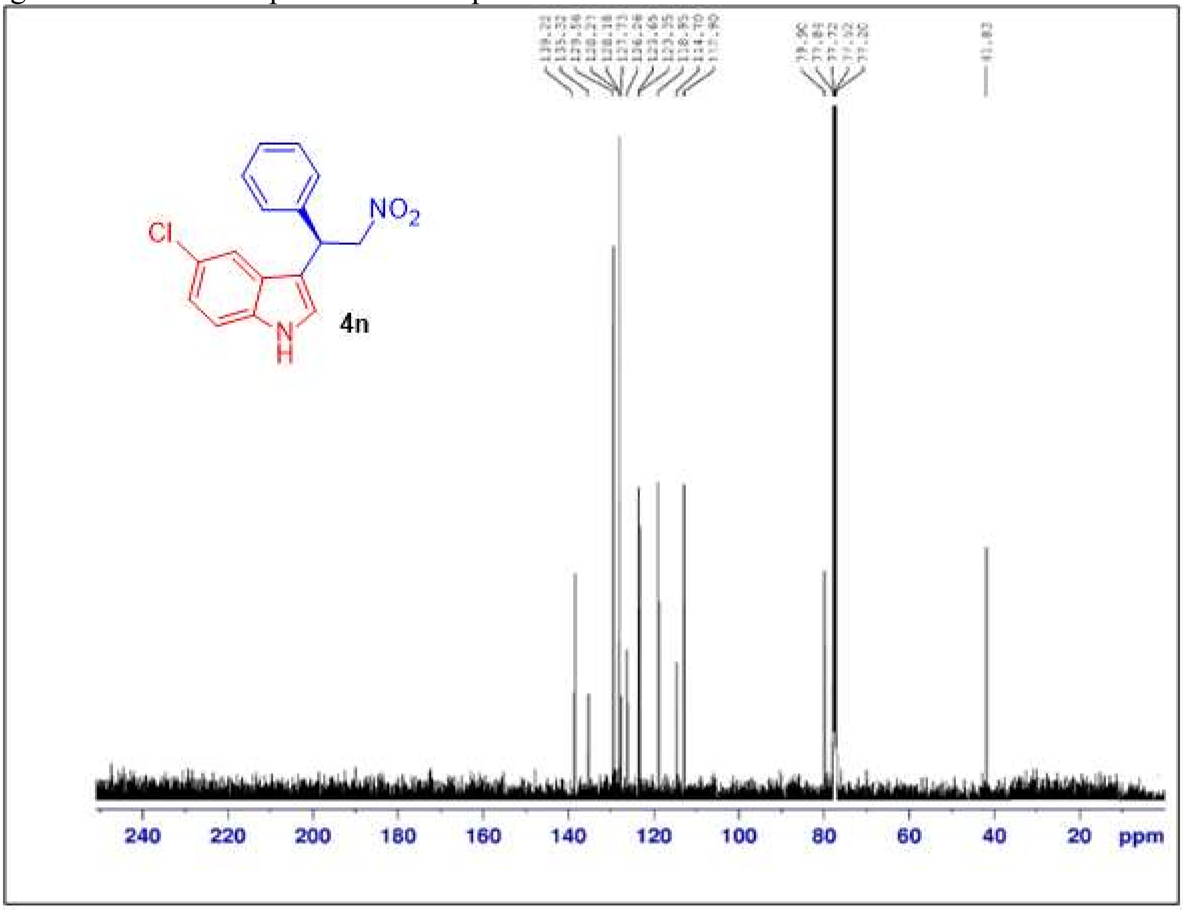

Figure S64: ^{13}CNMR spectrum of compound **4n**

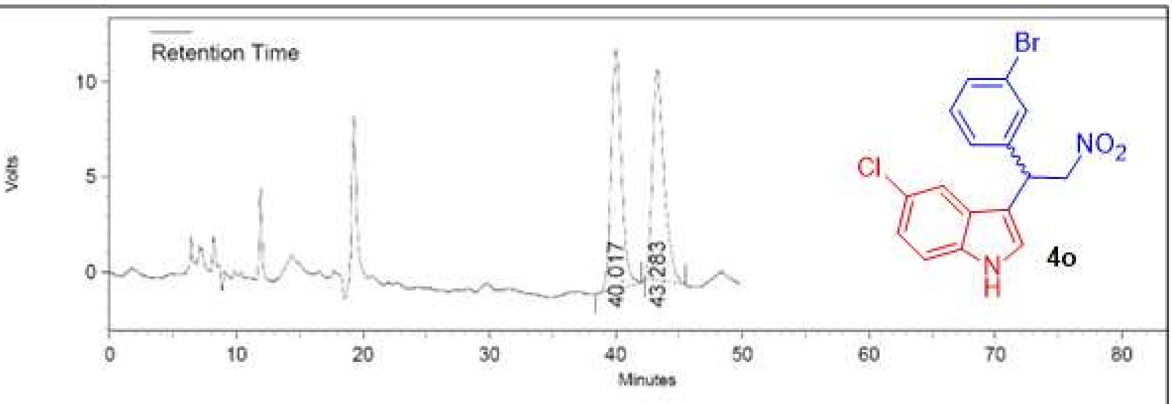

UV [1] Results

Retention Time	Area	Area %	Height	Height %
40.017	705799	49.31	12660	53.28
43.283	725547	50.69	11102	46.72
Totals	1431346	100.00	23762	100.00

Figure S65: HPLC chromatogram for racemic mixture of **4o**

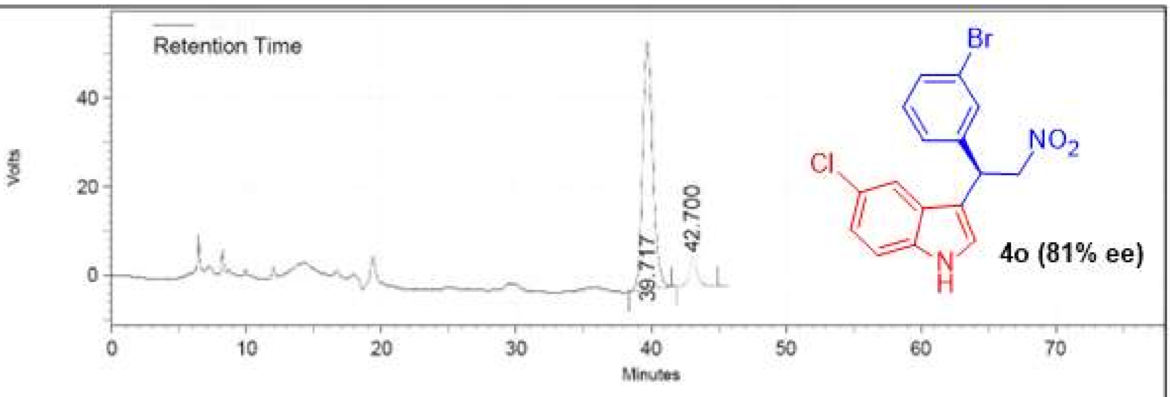

UV [1] Results

Retention Time	Area	Area %	Height	Height %
39.717	2981551	90.53	55818	82.54
42.700	311866	9.47	4809	17.46
Totals	3293417	100.00	67627	100.00

Figure S66: HPLC chromatogram for chiral **4o**

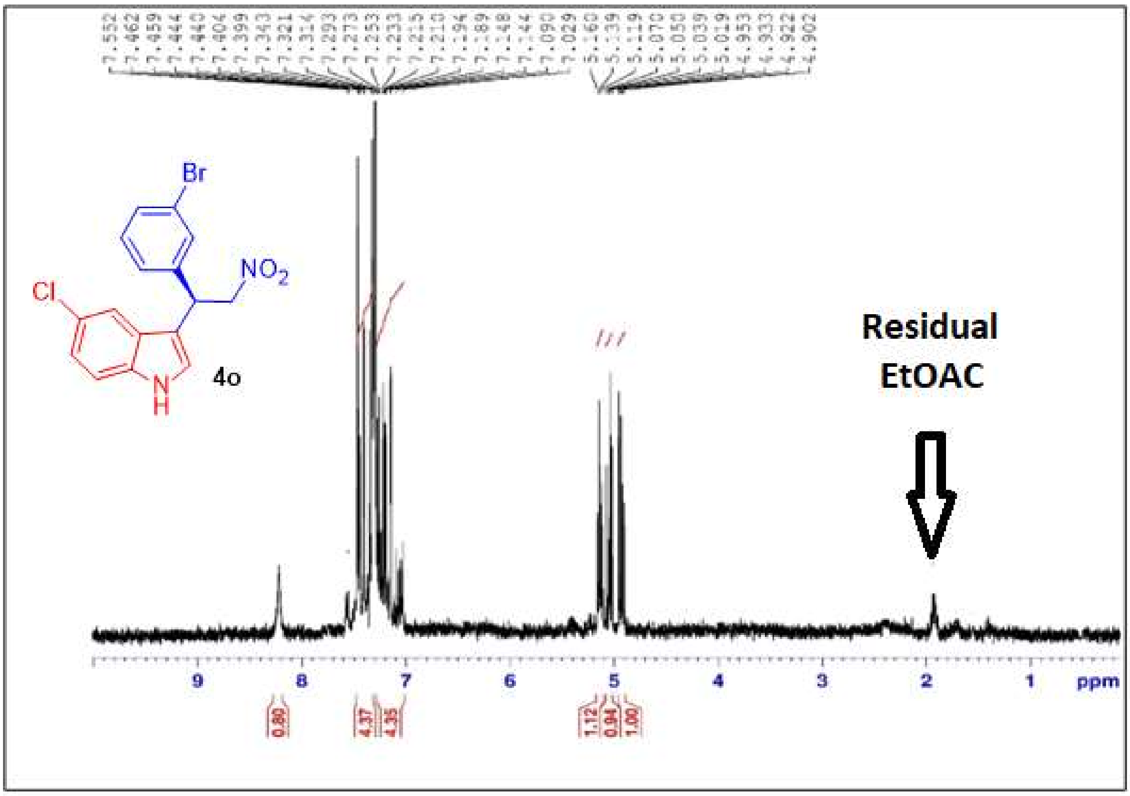

Figure S67: ^{1}HNMR spectrum of compound **4o**

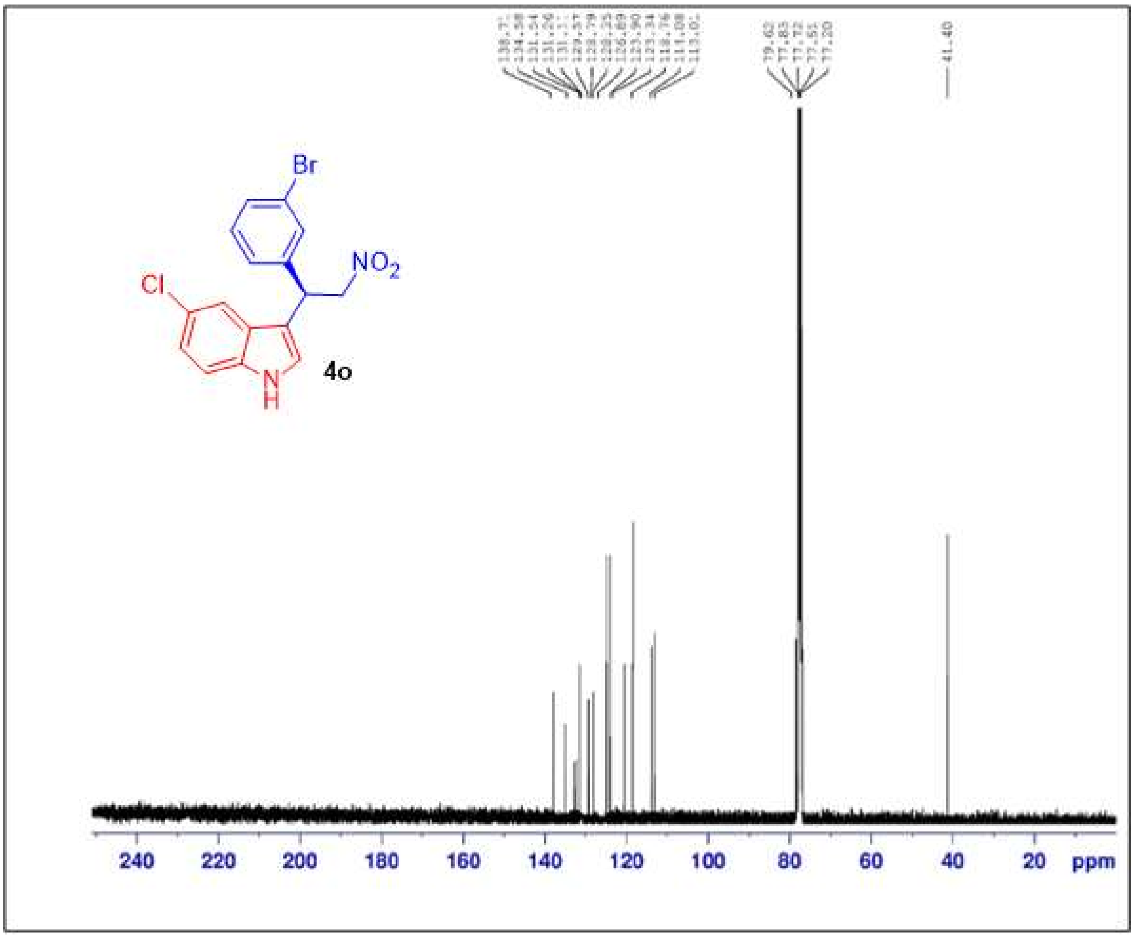

Figure S68: ^{13}CNMR spectrum of compound **4o**

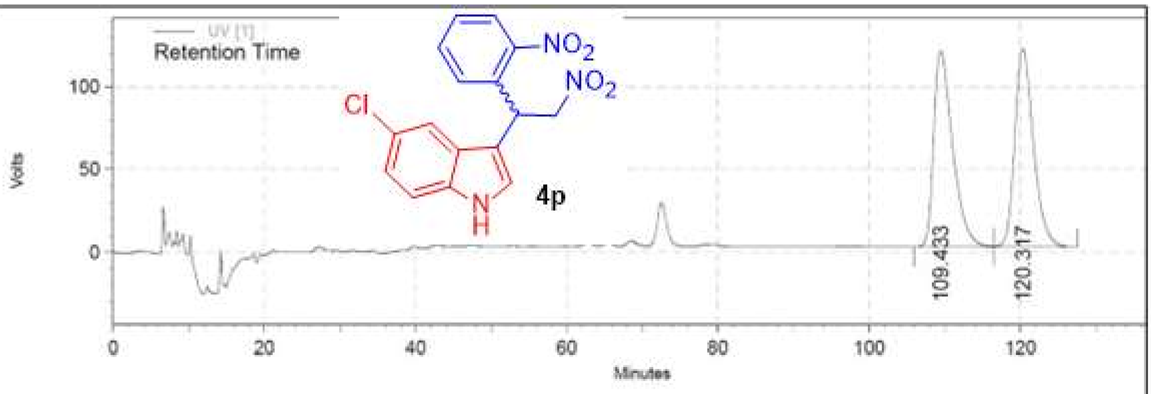

UV [1] Results

Retention Time	Area	Area %	Height	Height %
109.433	20706041	49.97	118520	49.65
120.317	20728832	50.03	120176	50.35
Totals	41434873	100.00	238696	100.00

Figure S69: HPLC chromatogram for racemic mixture of **4p**

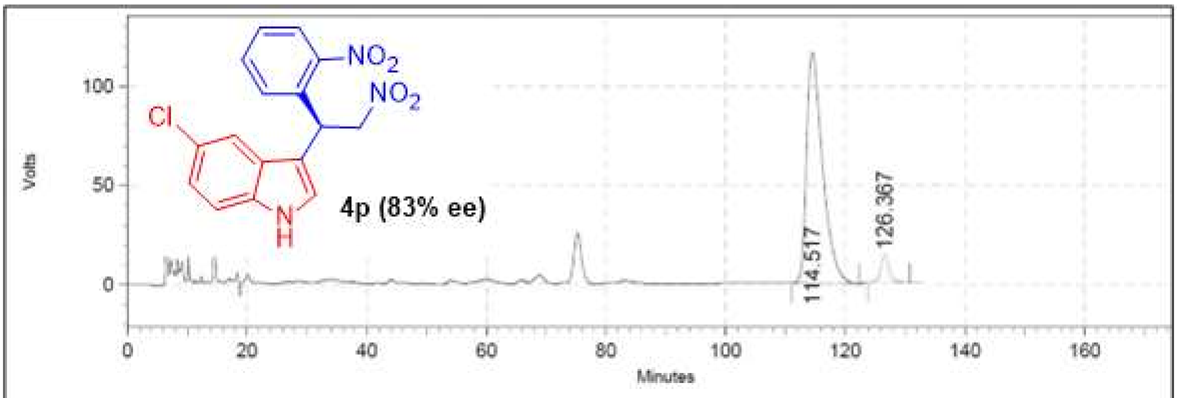

UV [1] Results

Retention Time	Area	Area %	Height	Height %
114.517	21632416	91.66	116527	86.36
126.367	1968845	8.34	13410	13.64
Totals	23601261	100.00	134937	100.00

Figure S70: HPLC chromatogram for chiral **4p**

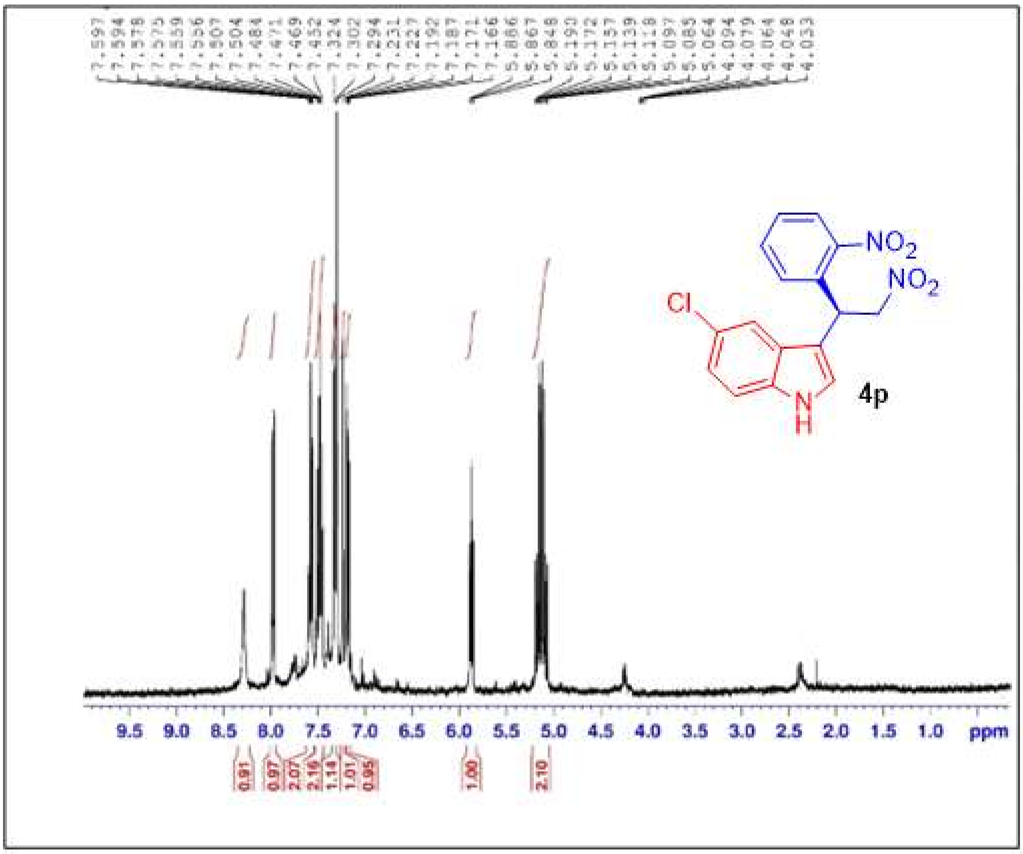

Figure S71: ^{1}HNMR spectrum of compound **4p**

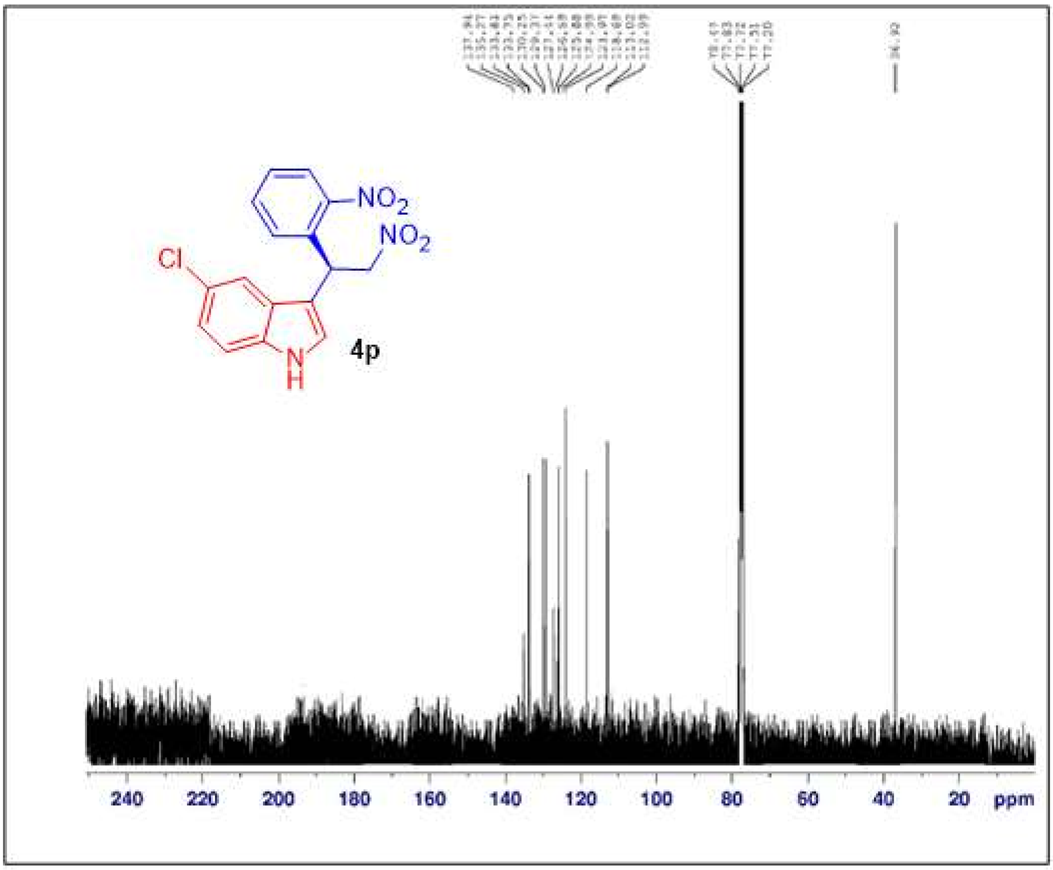

Figure S72: ^{13}CNMR spectrum of compound **4p**

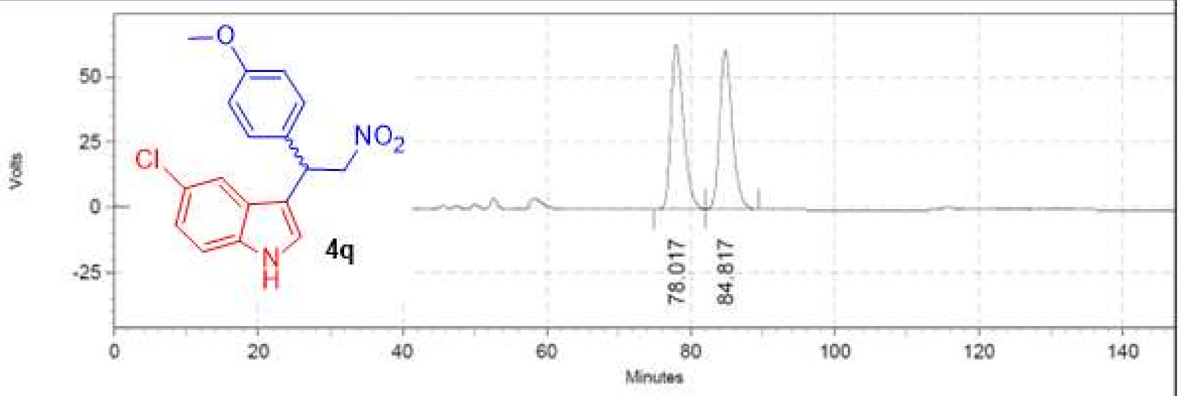

UV [1] Results

Retention Time	Area	Area %	Height	Height %
78.017	7602939	50.07	62901	50.77
84.817	7580438	49.93	60996	49.23
Totals	15183377	100.00	123897	100.00

Figure S73: HPLC chromatogram for racemic mixture of **4q**

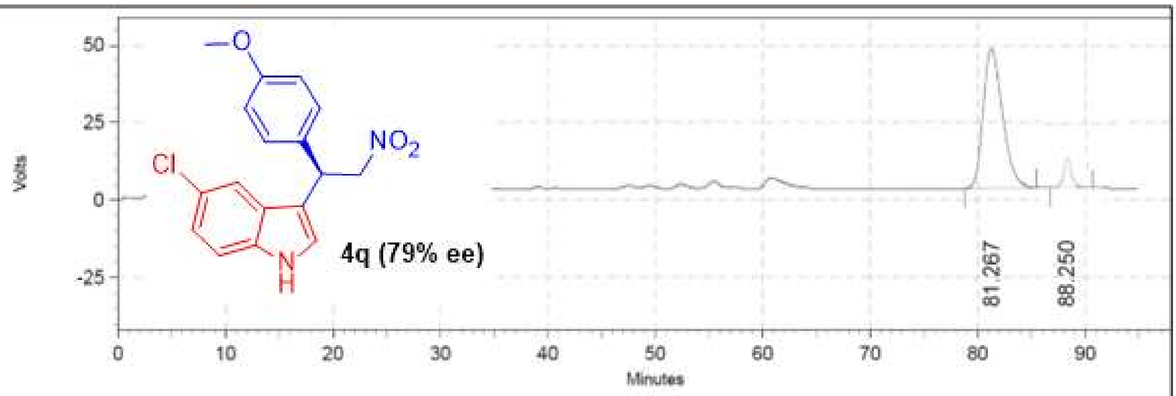

UV [1] Results

Retention Time	Area	Area %	Height	Height %
81.267	5588611	89.56	44989	83.20
88.250	651755	10.44	9083	16.80
Totals	6240366	100.00	54072	100.00

Figure S74: HPLC chromatogram for chiral **4q**

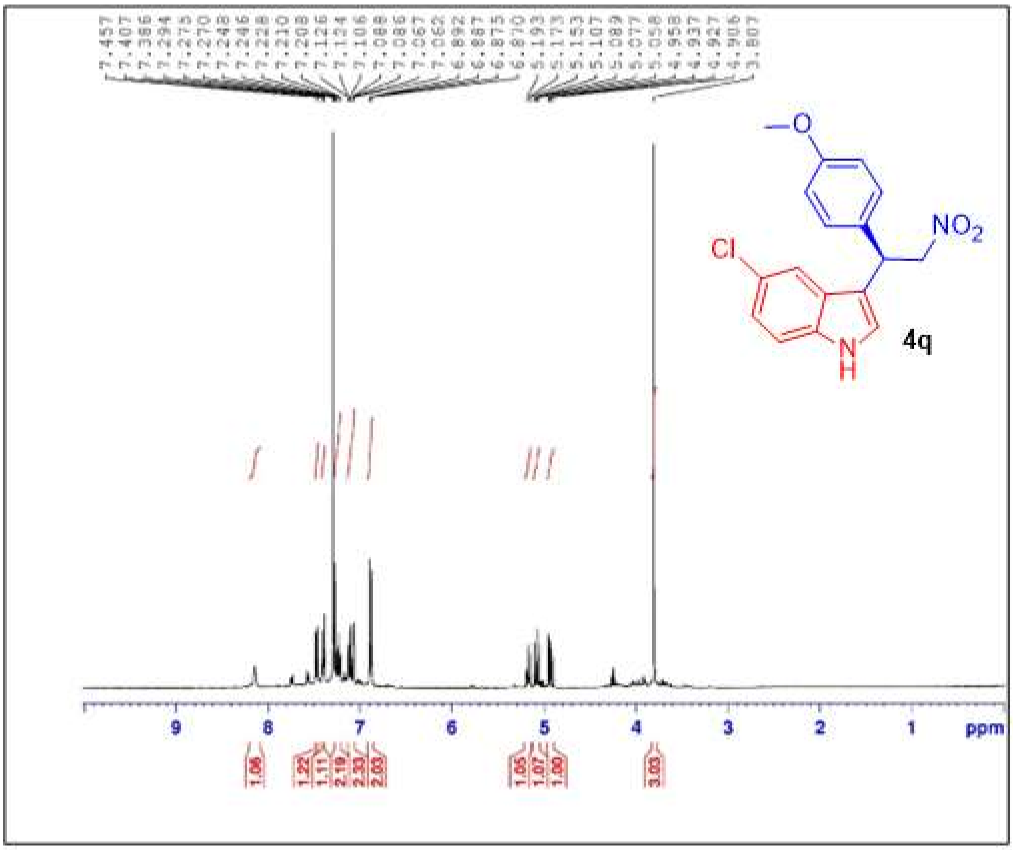

Figure S75: ^{1}HNMR spectrum of compound **4q** (purity of product is 82% according to ^{1}HNMR)

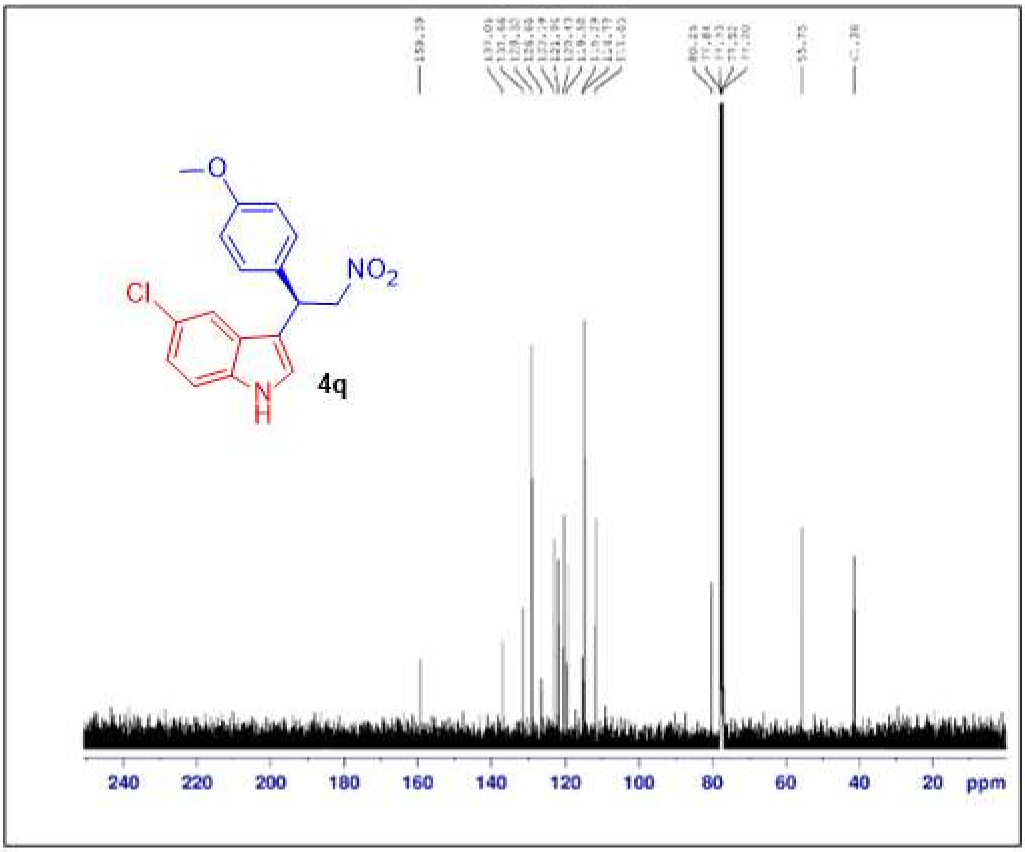

Figure S76: ^{13}CNMR spectrum of compound **4q**

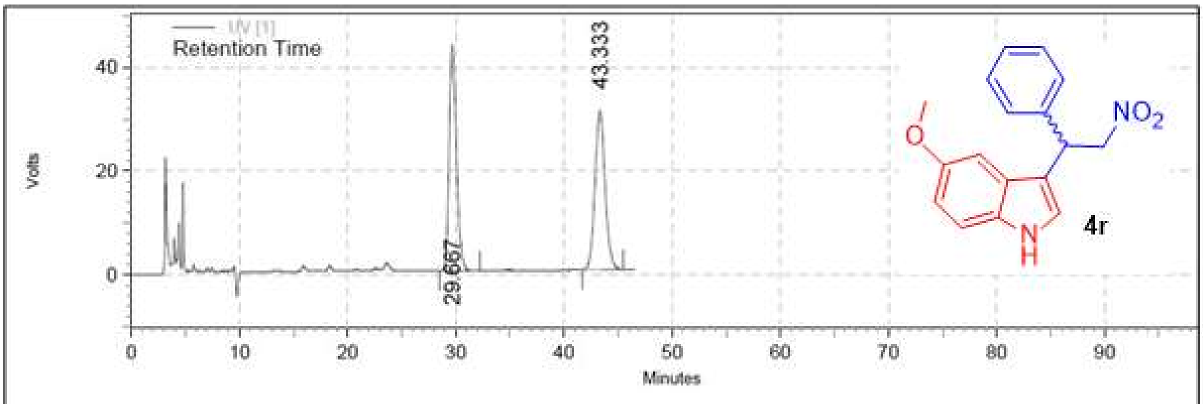

UV [1] Results

Retention Time	Area	Area %	Height	Height %
29.667	2012051	50.55	43565	58.78
43.333	1968414	49.45	30552	41.22
Totals	3980465	100.00	74117	100.00

Figure S77: HPLC chromatogram for racemic mixture of **4r**

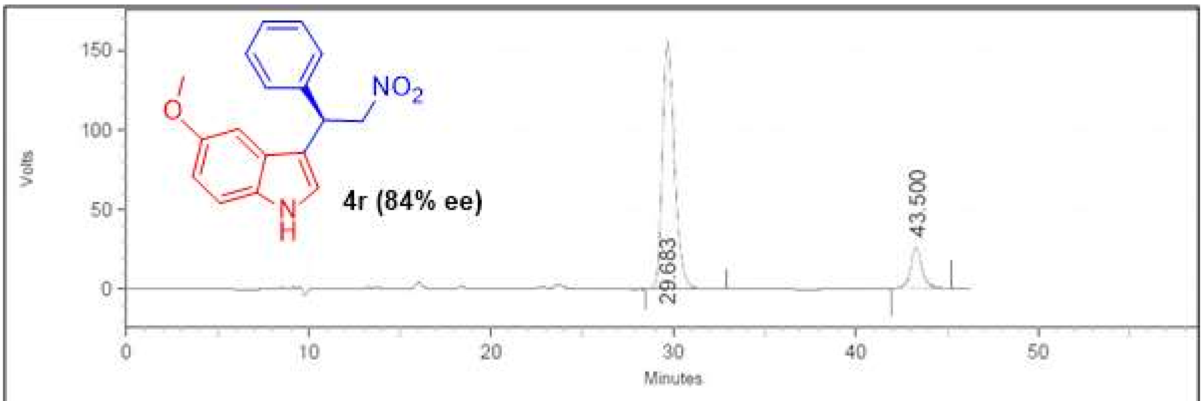

UV [1] Results

Retention Time	Area	Area %	Height	Height %
29.683	7197657	92.05	155711	91.36
43.500	621559	7.95	24726	8.64
Totals	7819216	100.00	170437	100.00

Figure S78: HPLC chromatogram for chiral **4r**

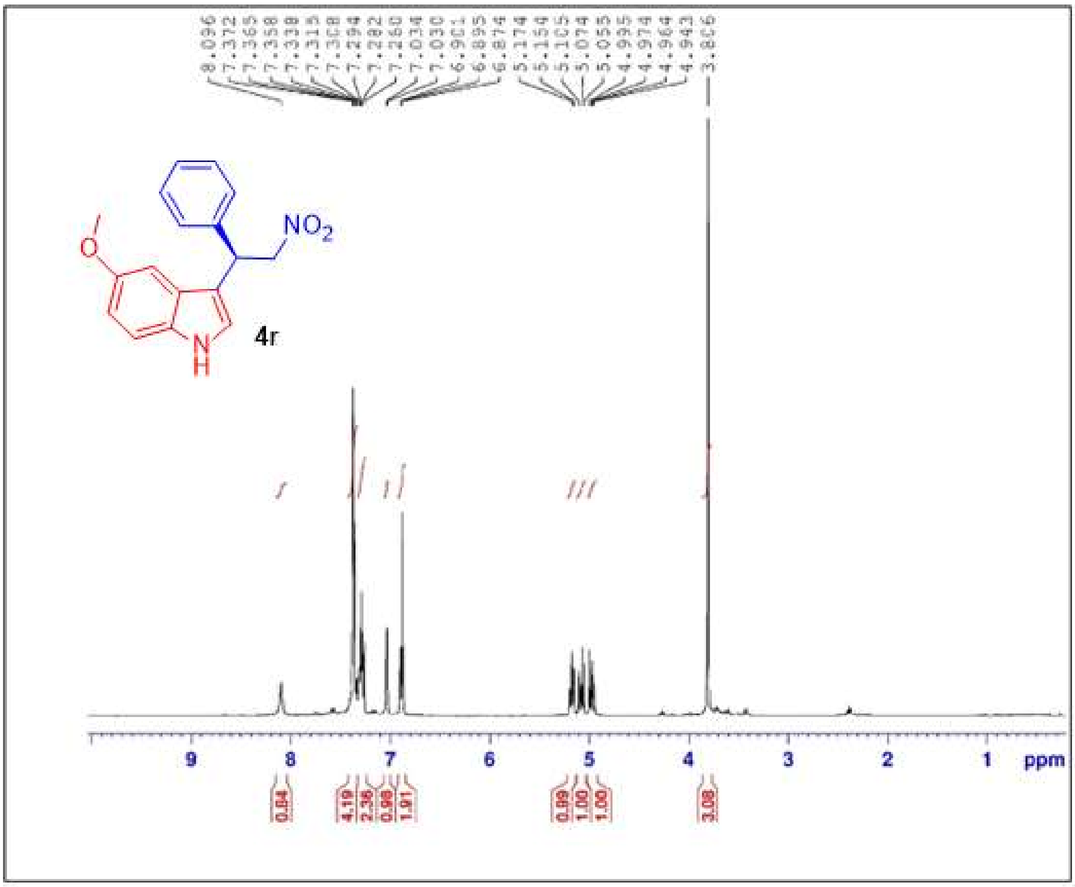

Figure S79: ^{1}HNMR spectrum of compound **4r**

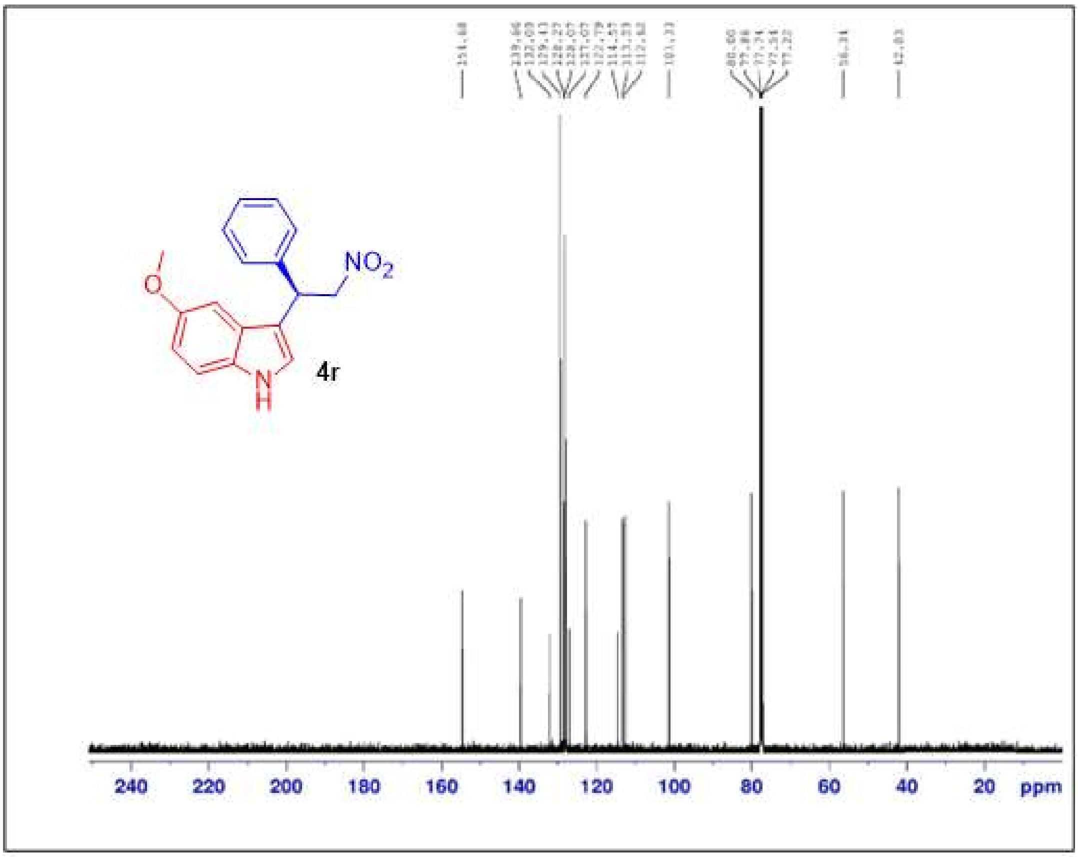

Figure S80: ^{13}CNMR spectrum of compound **4r**

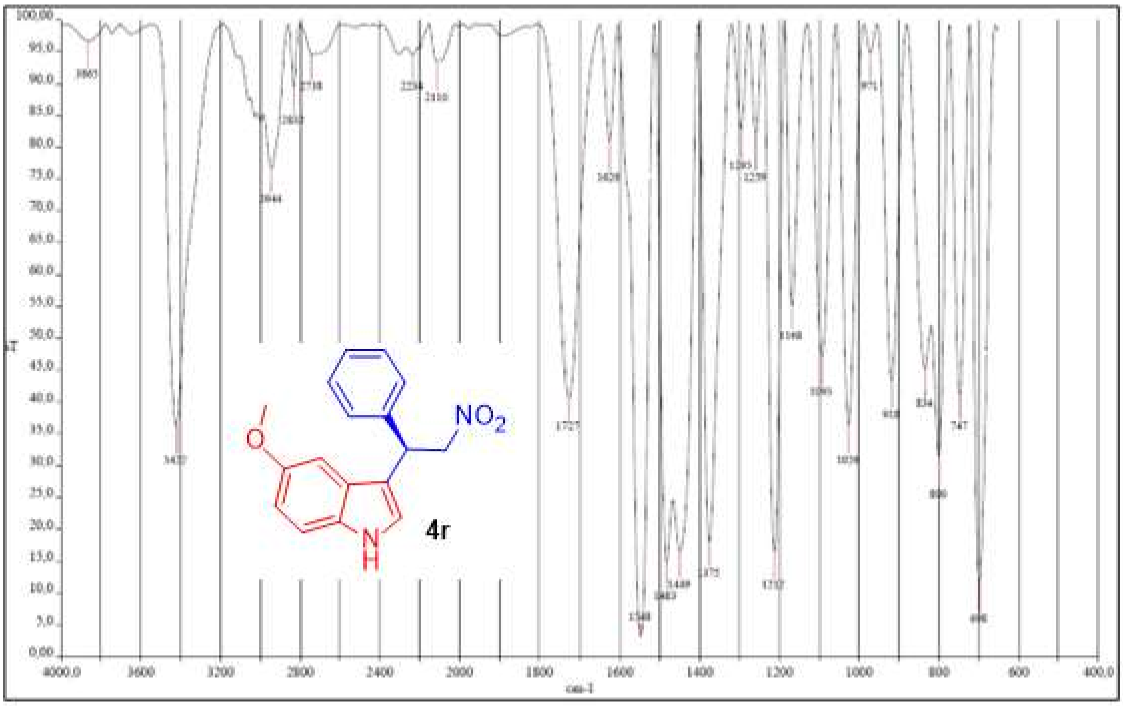

Figure S81: FTIR spectrum of compound **4r**

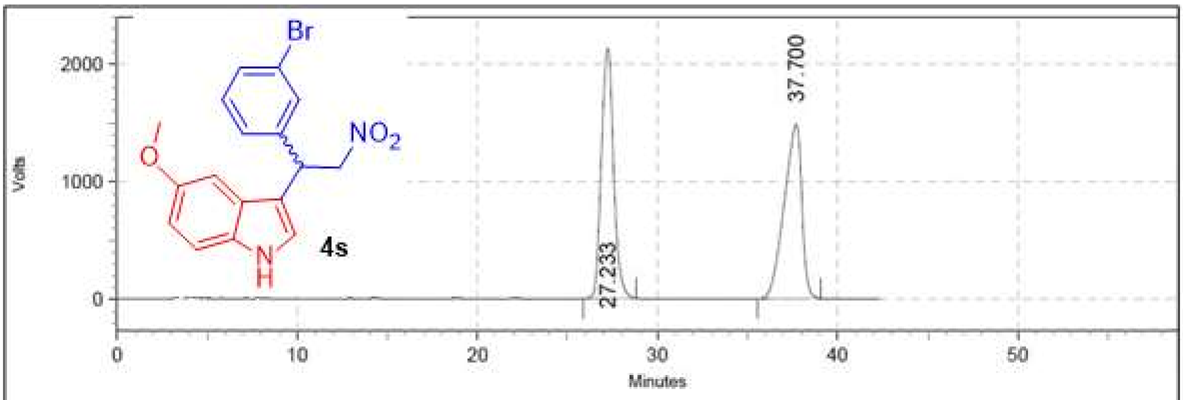

UV [1] Results

Retention Time	Area	Area %	Height	Height %
27.233	100787988	49.95	2128841	58.92
37.700	100995021	50.05	1484480	41.08
Totals	201783009	100.00	3613321	100.00

Figure S82: HPLC chromatogram for racemic mixture of **4s**

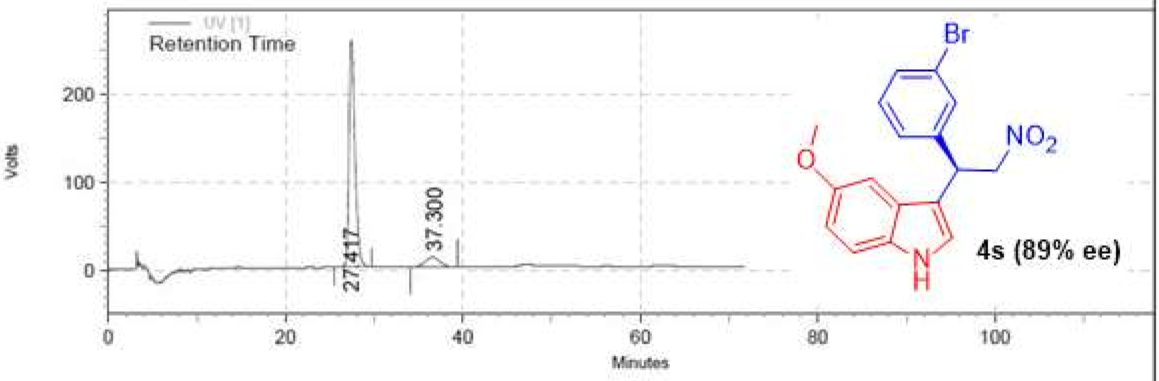

UV [1] Results

Retention Time	Area	Area %	Height	Height %
27.417	11944844	94.42	257661	95.44
37.300	706424	5.58	12311	4.56
Totals	12651268	100.00	269972	100.00

Figure S83: HPLC chromatogram for chiral **4s**

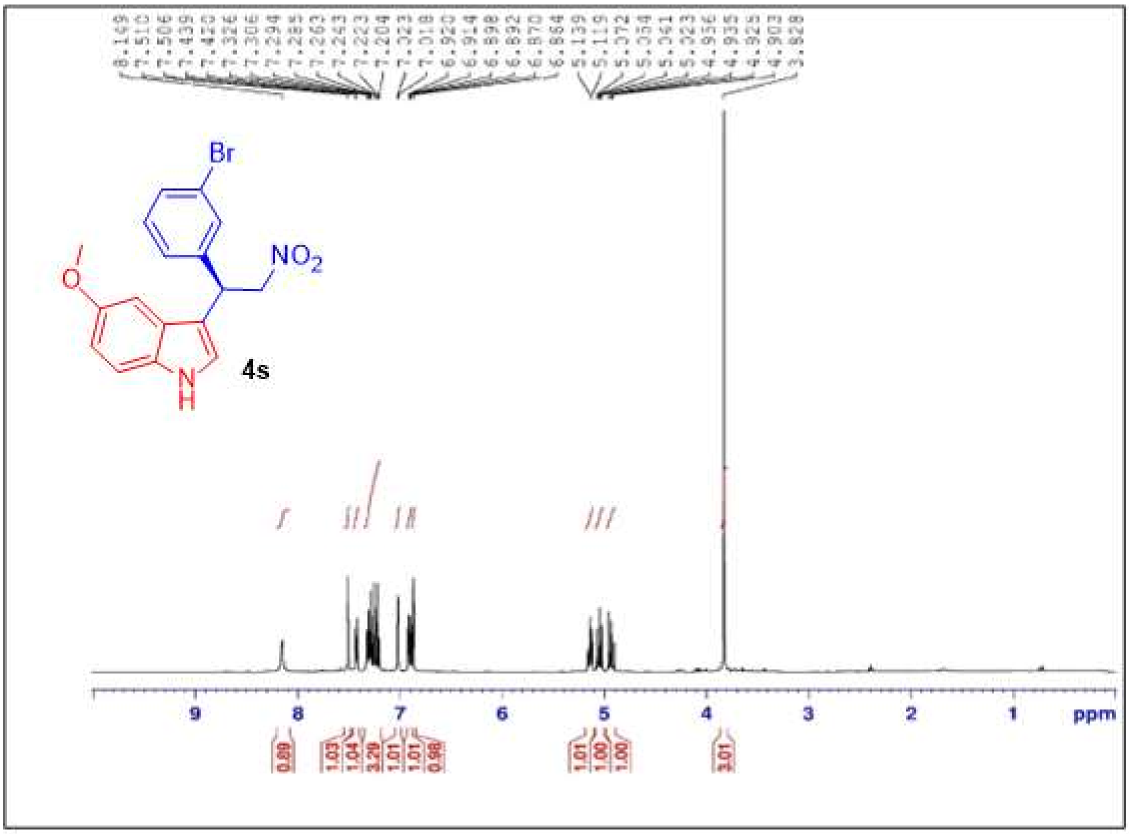

Figure S84: ^{1}HNMR spectrum of compound **4s**

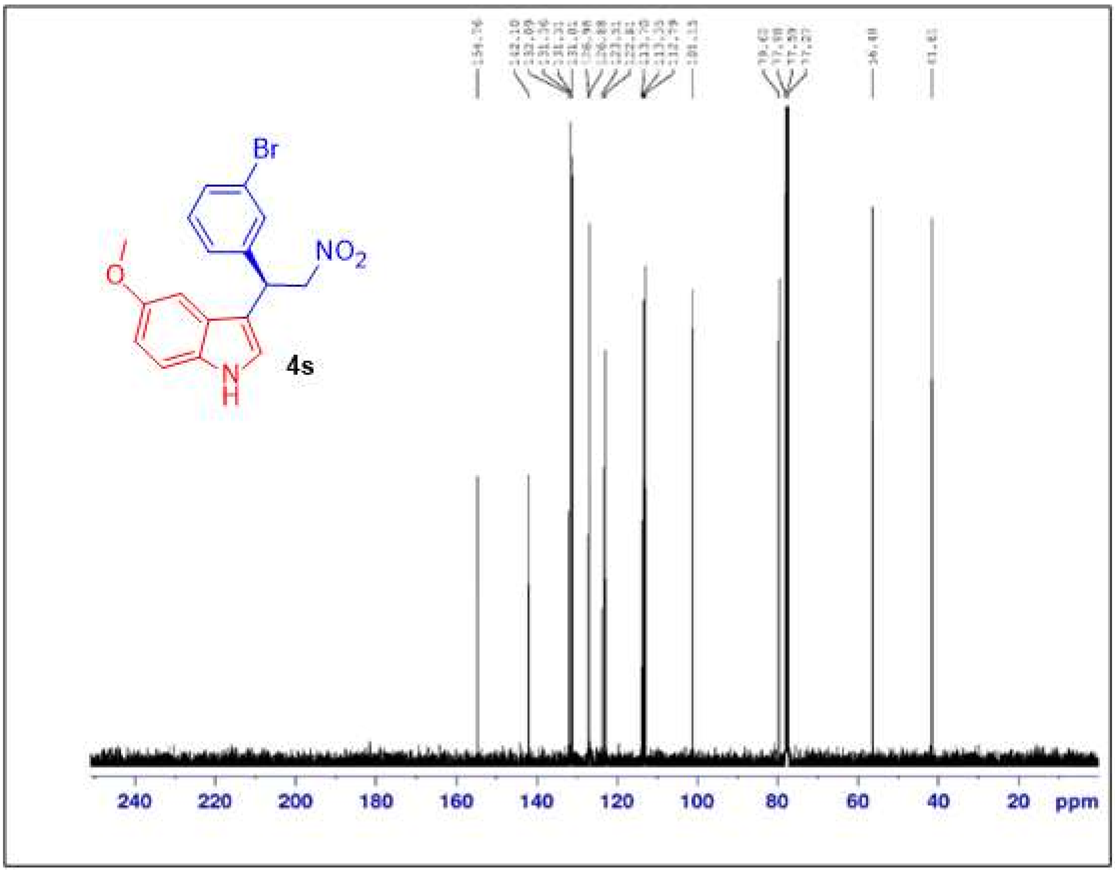

Figure S85: ^{13}CNMR spectrum of compound **4s**

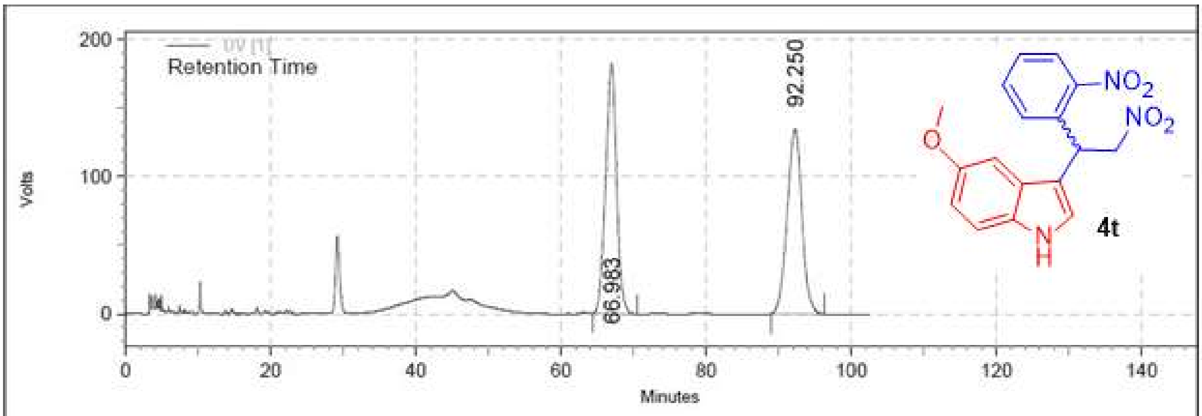

UV [1] Results

Retention Time	Area	Area %	Height	Height %
66.983	19212403	50.35	182555	57.58
92.250	18945566	49.65	134487	42.42
Totals	38157969	100.00	317042	100.00

Figure S86: HPLC chromatogram for racemic mixture of **4t**

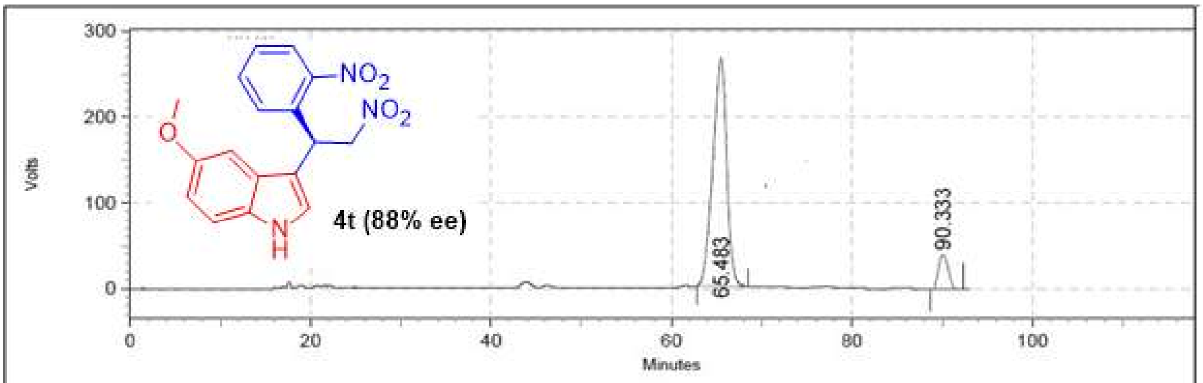

UV [1] Results

Retention Time	Area	Area %	Height	Height %
65.483	28428770	93.93	265920	94.96
90.333	1835731	6.07	14109	5.04
Totals	30264501	100.00	280029	100.00

Figure S87: HPLC chromatogram for chiral **4t**

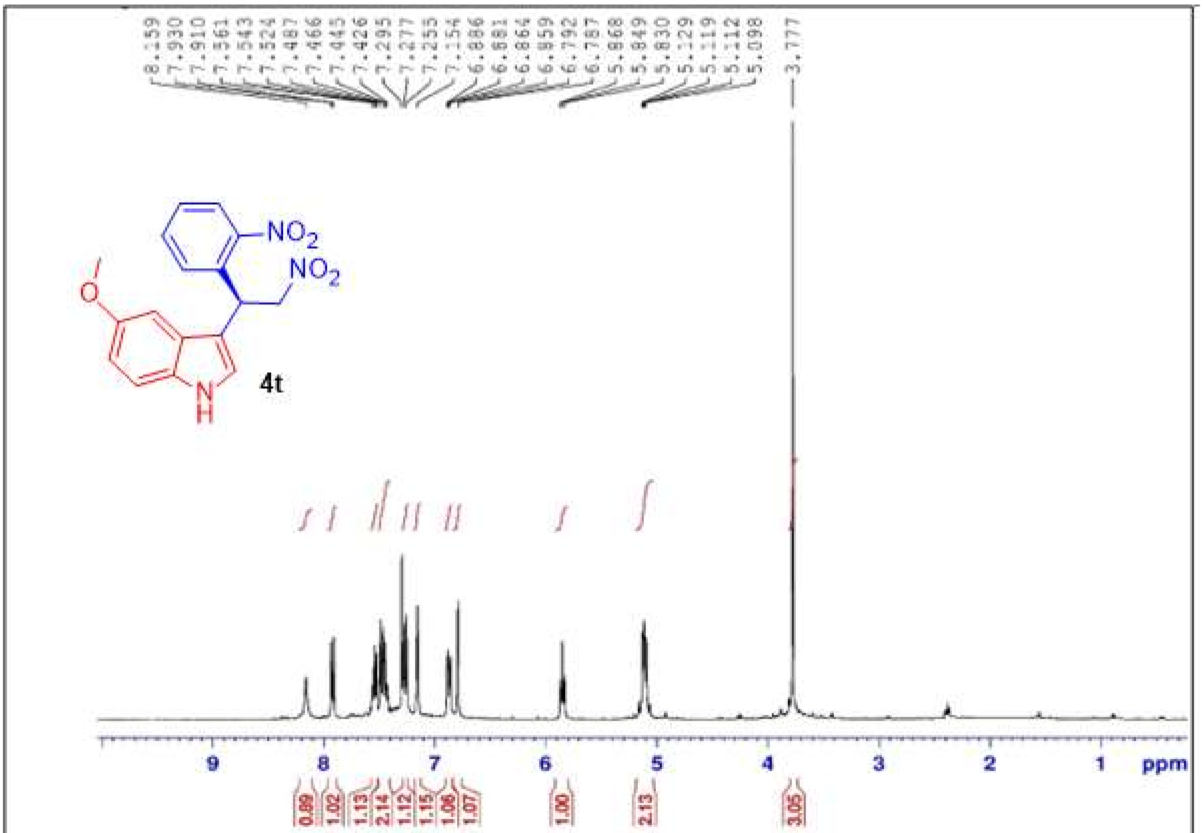

Figure S88: ^{1}HNMR spectrum of compound **4t**

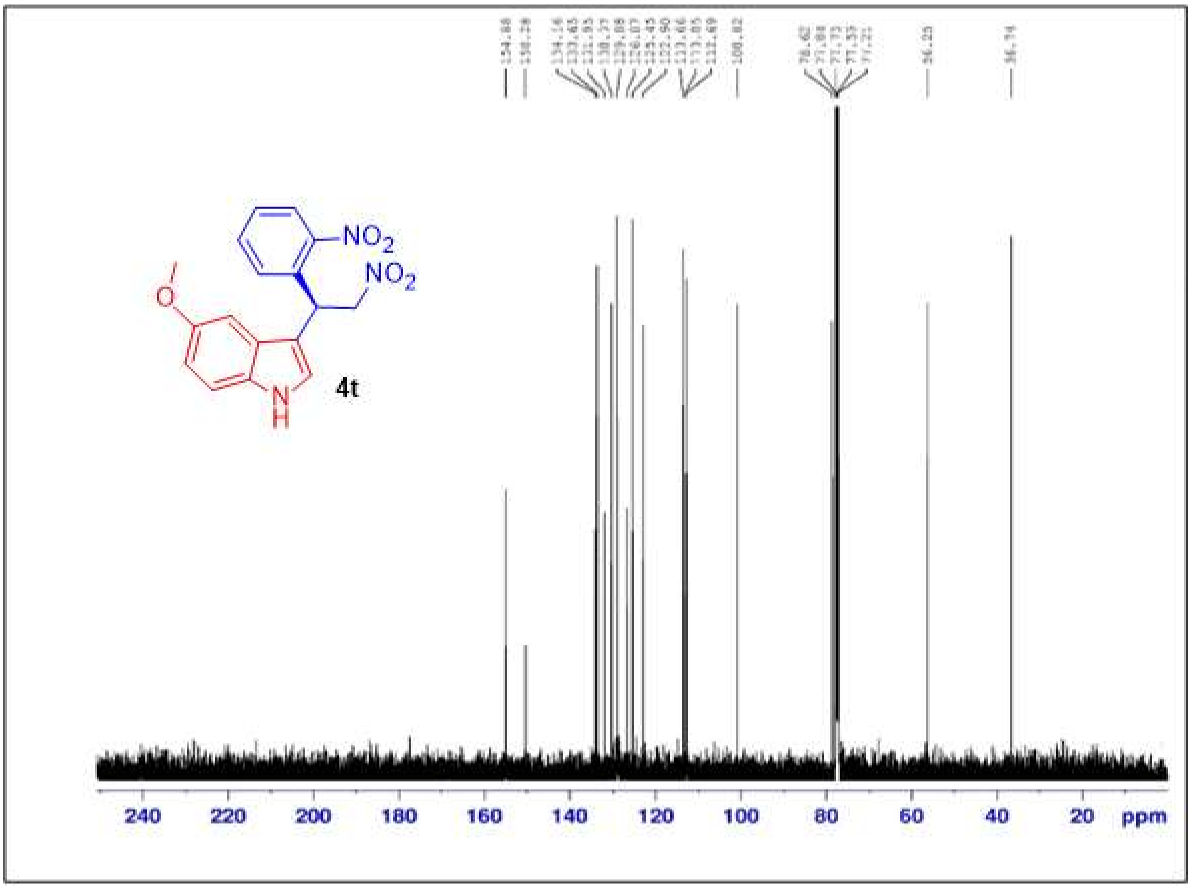

Figure S89: ^{13}CNMR spectrum of compound **4t**

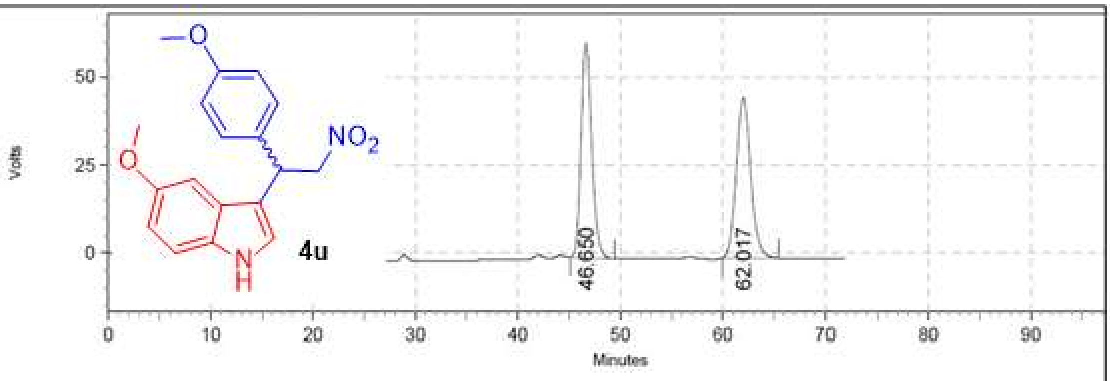

UV [1] Results

Retention Time	Area	Area %	Height	Height %
46.650	4453137	49.70	61076	57.26
62.017	4507103	50.30	45590	42.74
Totals	8960240	100.00	106666	100.00

Figure S90: HPLC chromatogram for racemic mixture of **4u**

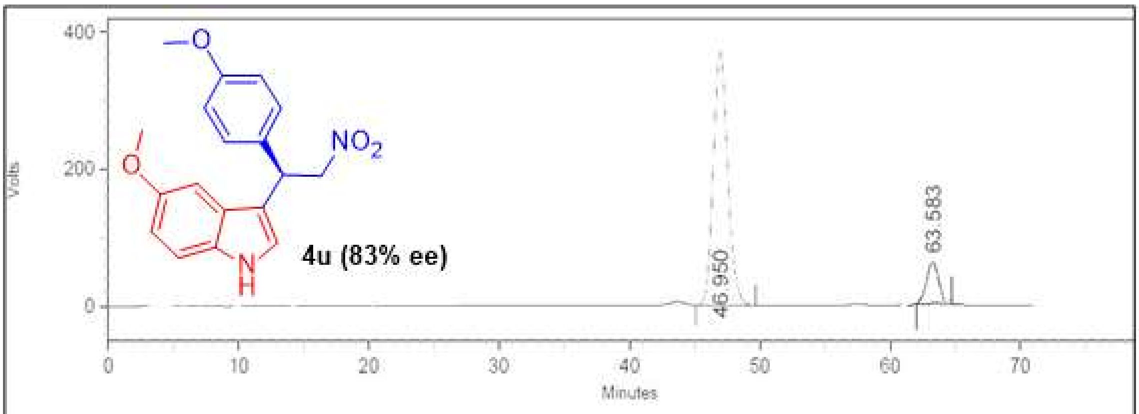

UV [1] Results

Retention Time	Area	Area %	Height	Height %
46.950	28512253	91.57	371469	92.19
63.583	2624337	8.43	31465	7.81
Totals	31136590	100.00	402934	100.00

Figure S91: HPLC chromatogram for chiral **4u**

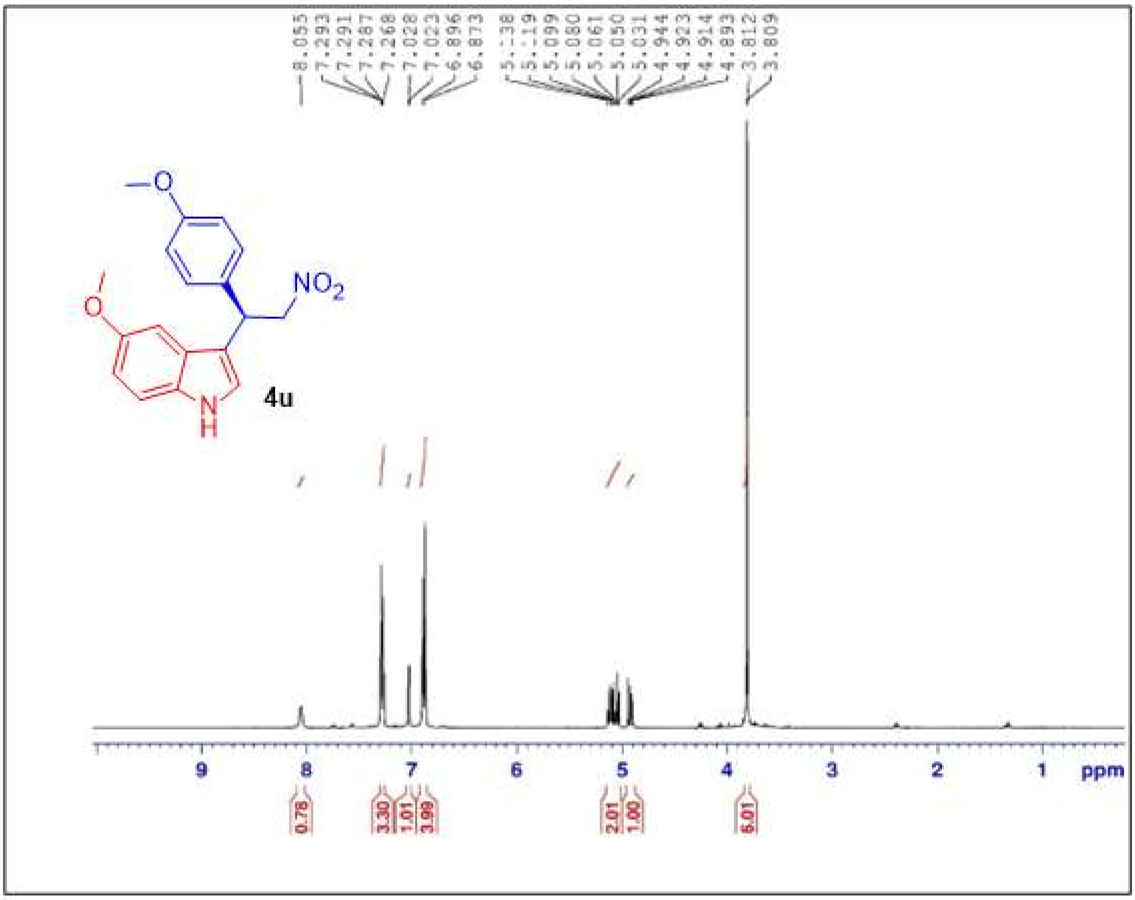

Figure S92: ^{1}HNMR spectrum of compound **4u**

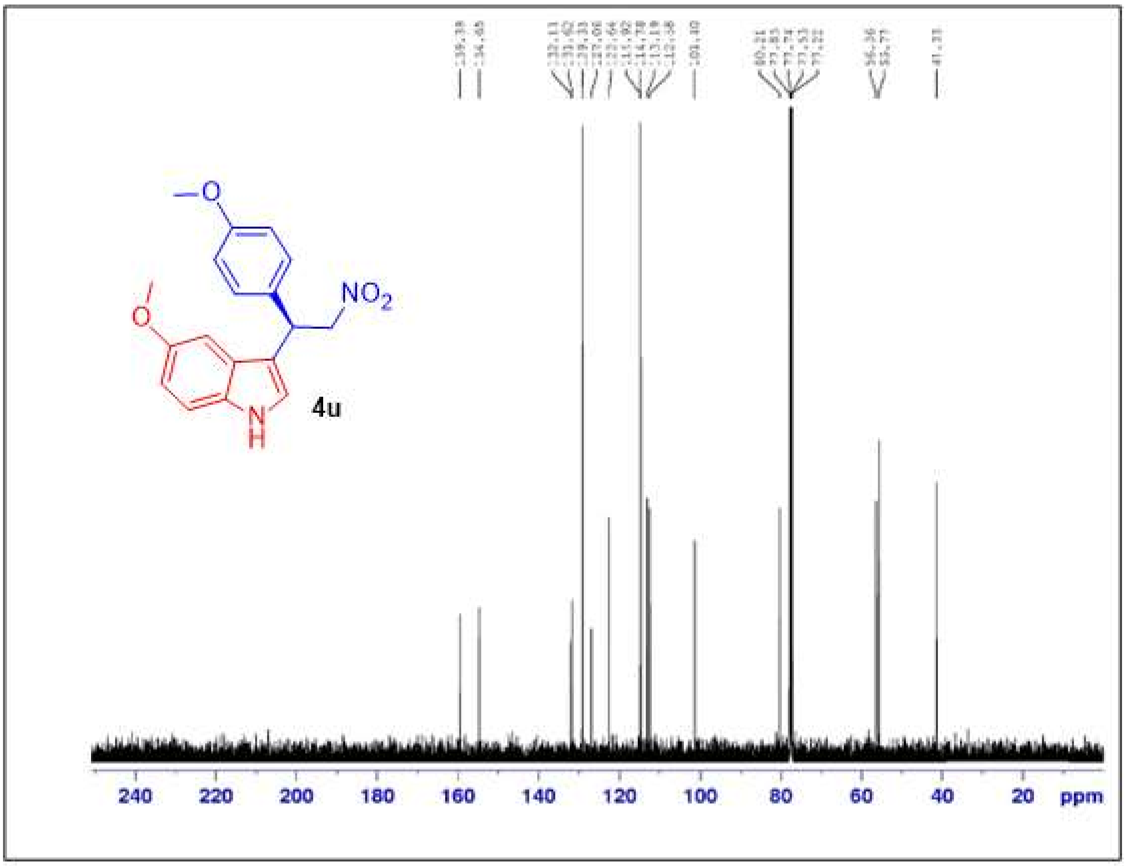

Figure S93: ^{13}CNMR spectrum of compound **4u**

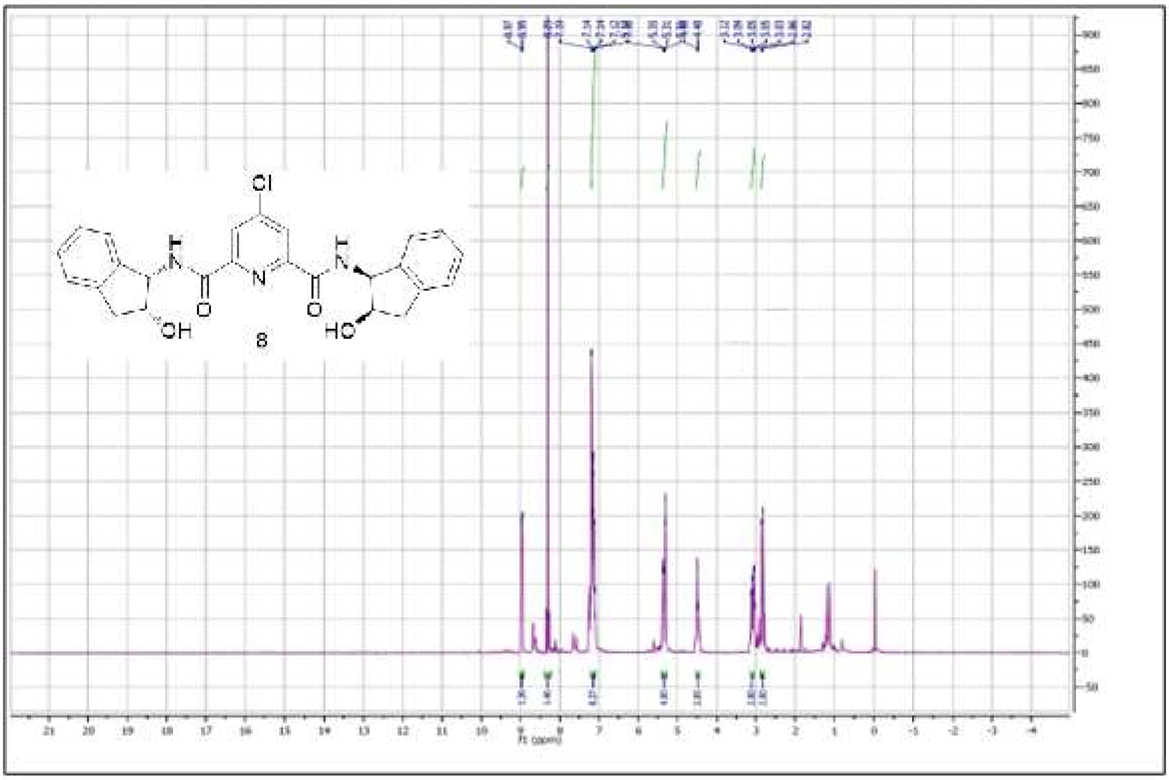

Figure S94: ^{1}HNMR spectrum of compound **8**

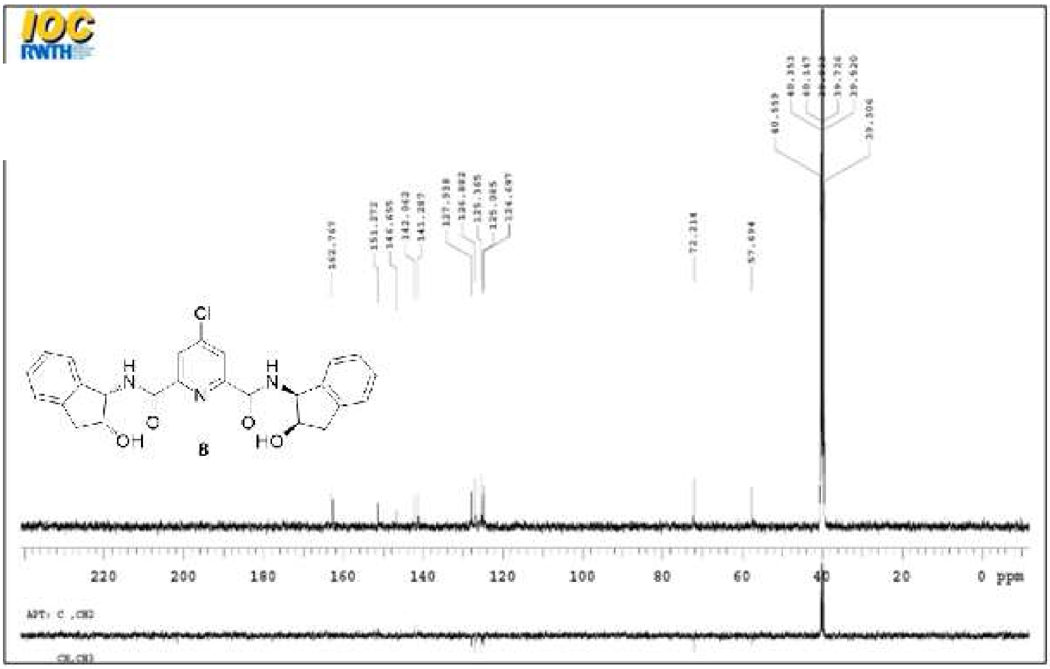

Figure S95: ^{13}CNMR spectrum of compound **8**

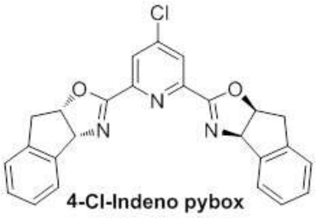

4-Cl-Indeno pybox
1g

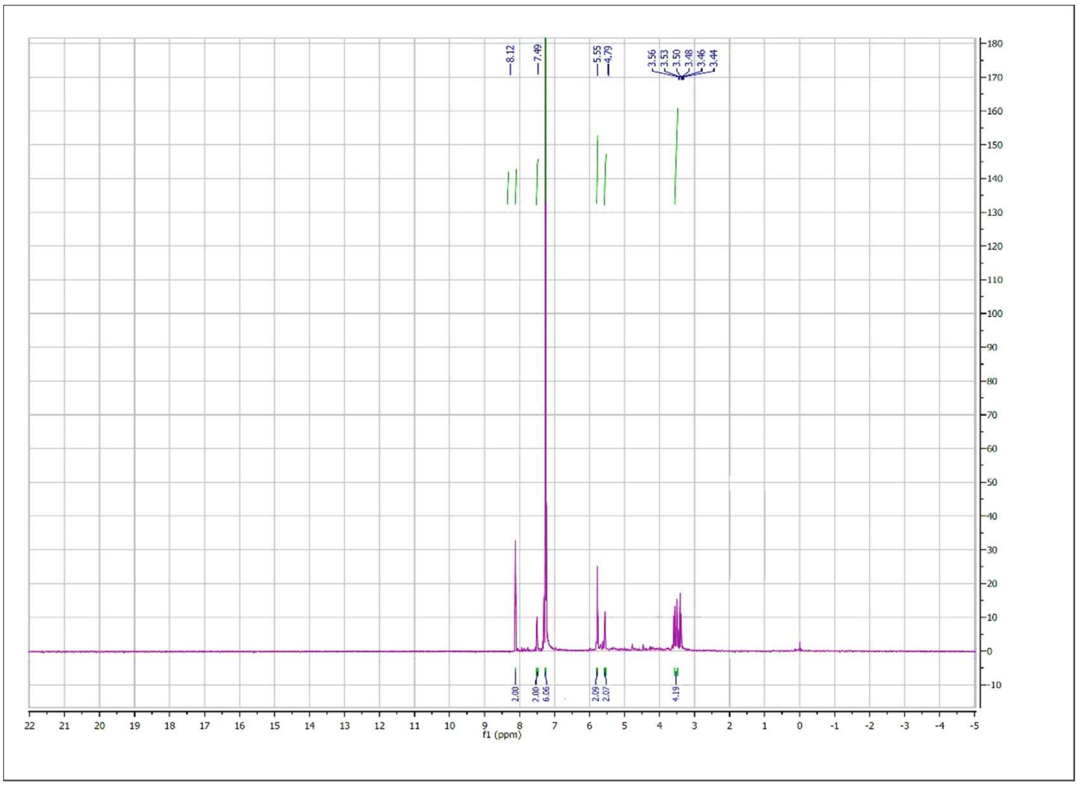

Figure S96: ^{1}HNMR spectrum of compound **4-Cl-Indeno pybox 1g**

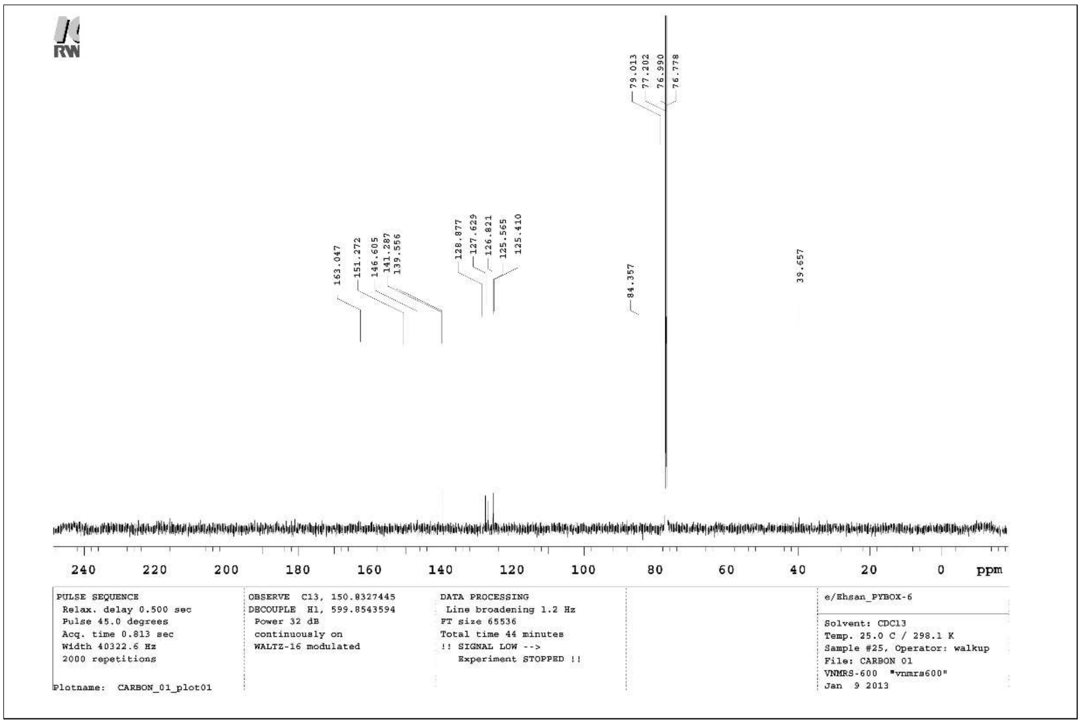

Figure S97: ^{13}CNMR spectrum of compound **4-Cl-Indeno pybox 1g**

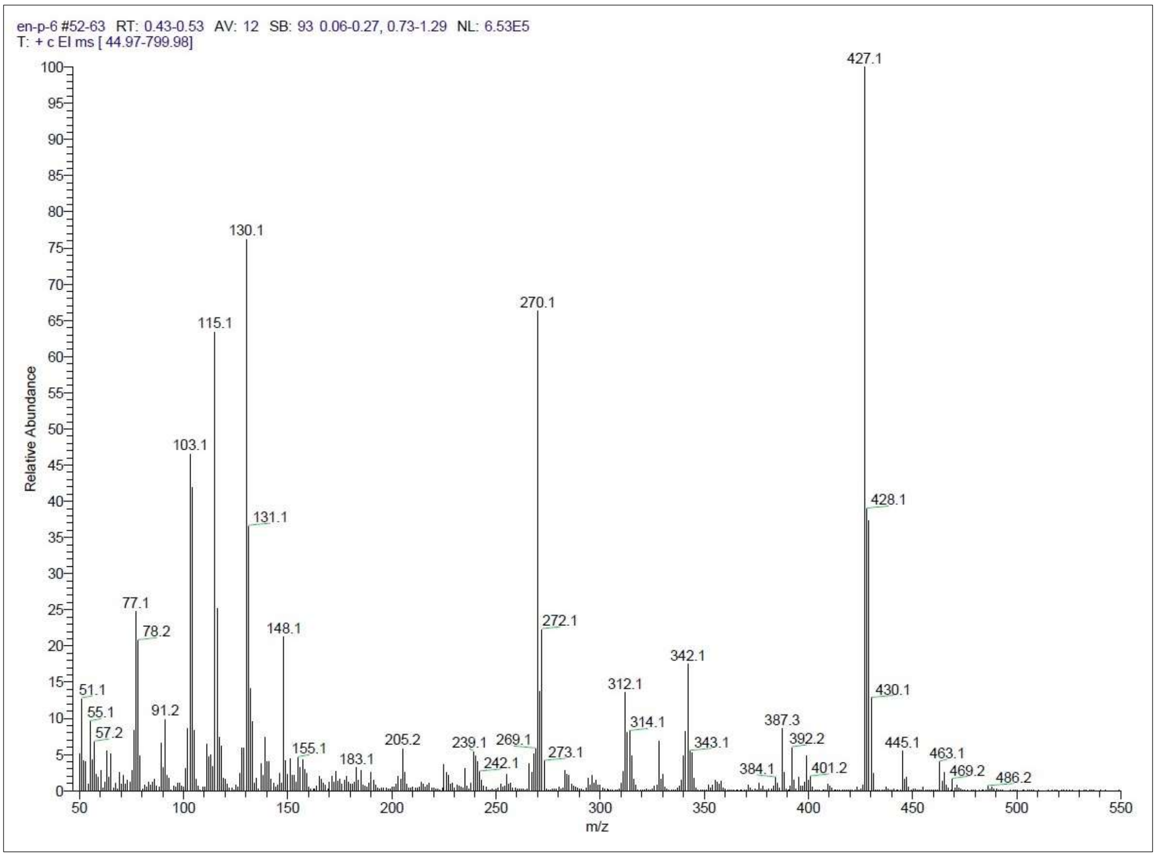

Figure S98: MS spectrum of compound **4-Cl-Indeno pybox 1g**

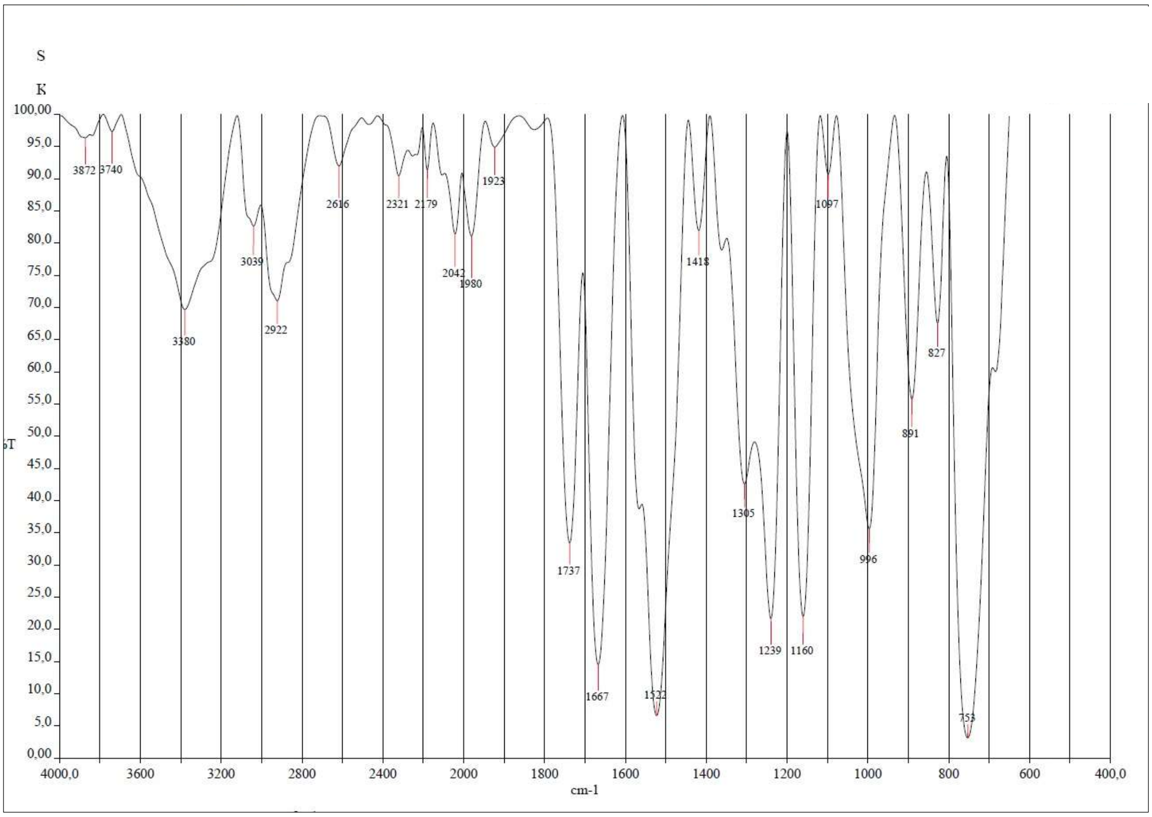

Figure S99: FTIR spectrum of compound **4-Cl-Indeno pybox 1g**
